# Intrinsically Multi‐Stable Spatial Linkages

**DOI:** 10.1002/advs.202402127

**Published:** 2024-09-16

**Authors:** Tong Zhou, Chong Huang, Zhuangzhi Miao, Yang Li

**Affiliations:** ^1^ The Institute of Technological Sciences Wuhan University Wuhan 430072 China; ^2^ Wuhan University Shenzhen Research Institute Shenzhen 518057 China

**Keywords:** compliant mechanisms, deployable tube, impulsive grippers, mechanical multistability, spatial linkages

## Abstract

Multi‐stable structures can be reconfigured with fewer, lightweight, and less accurate actuators. This is because the attraction domain in the multi‐stable energy landscape provides both reconfiguration guidance and shape accuracy. Additionally, such structures can generate impulsive motion due to structural instability. Most multi‐stable units are planar structures, while spatial linkages can generate complex 3D motion and hold a more promising potential for applications. This study proposes a generalized approach to design a type of intrinsically multi‐stable spatial (IMSS) linkages with multiple prescriptible configurations, which are structurally compatible, and naturally stable at these states. It reveals that all over‐constrained mechanisms can be transformed into multi‐stable structures with the same design method. Single‐loop bi‐stable 4R and quadra‐stable 6R spatial linkages modules with intrinsic non‐symmetric stable states, which are transformed from fundamental kinematic linkage mechanisms unit such as Bennett and Bricard linkages, are designed to illustrate the basic idea and the superiority over the ordinary methods. Multi‐loop assembly by these IMSS linkage modules shows potential for practical applications that are required for the deployability and impulsivity of reconfiguration. Two preliminary design cases of a deployable tube and an impulsive gripper are experimentally presented to validate this applicability. Further promisingly, this design method of IMSS linkages paves the way for morphing platforms with lightweight actuation, high shape accuracy, high stiffness, and prescribed impulsive 3D motion.

## Graphical abstract

This study introduces a general design approach for intrinsically multi‐stable spatial linkages based on structural multi‐compatibility. Compared to classic methods, this methodology offers an extended design space for individual closed‐loop linkage modules to generate multiple (>3) asymmetric spatial configurations. It suggests all over‐constrained mechanisms could be transformed into multi‐stable structures. Practical applications include deployable tubes and impulsive grippers.

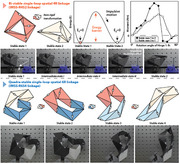

## 1 Introduction

Mechanical reconfigurability is a desirable property in robotics and metamaterials, which operate at different geometric configurations for different functions. Multi‐stable morphing structures, which can be self‐stable at several prescribed configurations,^[^ ^1^, ^2^ ^]^ have the following merits: 1) self‐locking at the operational states, which does not require constant actuation; 2) the domain of attraction in the energy landscape, which allows the employment of fewer inaccurate/lightweight actuators, such as smart materials; 3) the extended design space compared with over‐constrained mechanisms; 4) rapid shape transformation due to the structural instability, which is desired in some robotic applications.

**Figure 1:**
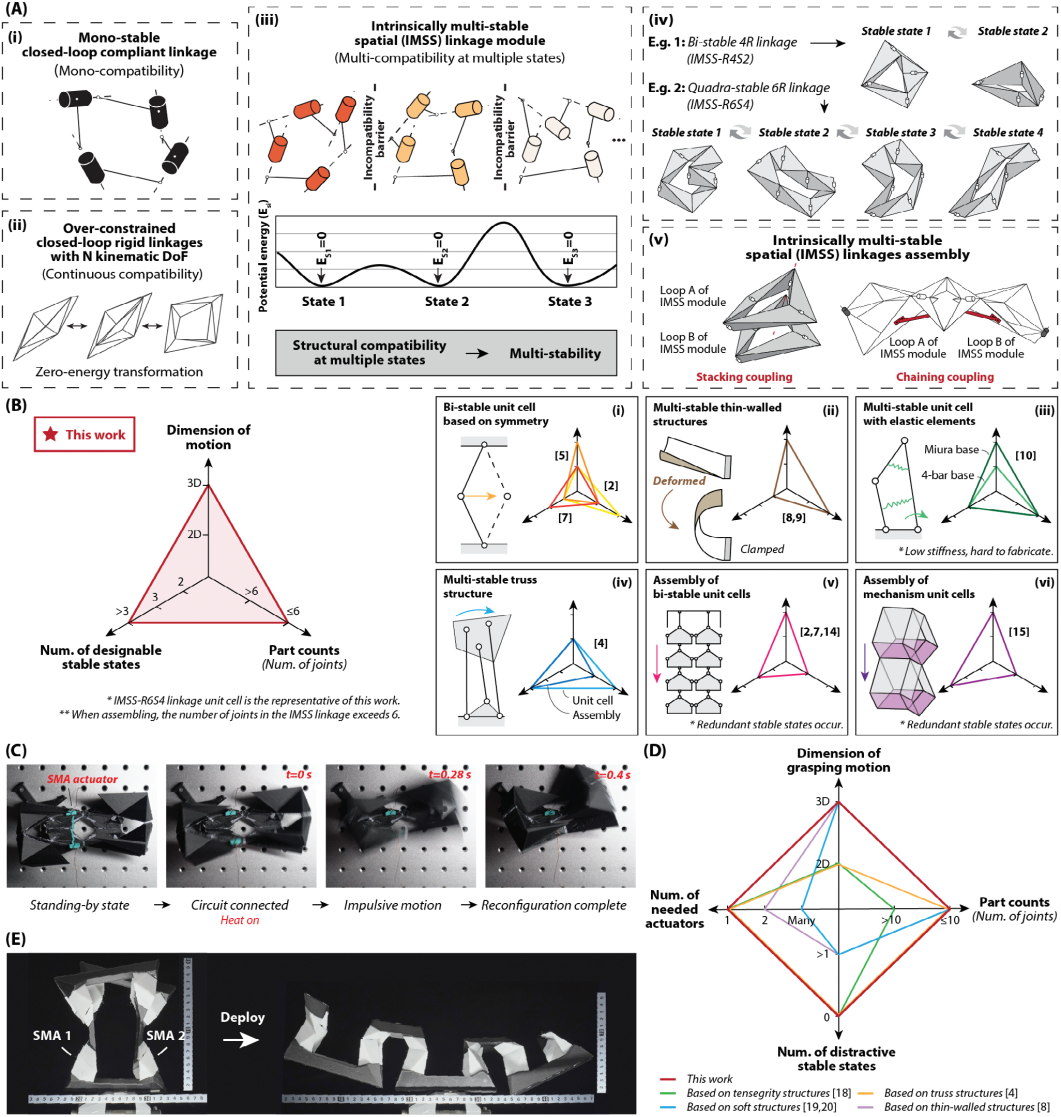
Design principle of intrinsically multi‐stable spatial (IMSS) linkages. A) Basic idea of IMSS linkage design based on multi‐compatibility principle. For a general mono‐stable linkage (i), its configuration is determined by the mono‐compatibility. However, if its compatibility is continuous, it turns out to be an over‐constrained linkage mechanism (ii). On the other hand, if the closure condition is satisfied only at the certain discrete configuration on the “kinematic path” (i.e., minimum energy path), the spatial linkage would be a multi‐compatible linkage (iii). Two example cases of a bi‐stable 4R linkage and a quadra‐stable 6R linkage is presented (iv). The constructive method by stacking and chaining strategies generates multi‐loop IMSS linkage assemblies (v) of a reconfigurable tube and an impulsive gripper. B) Comparison of IMSS linkages to other multi‐stable structures in the previous work.^[^ ^2^, ^4^, ^5^, ^7^, ^8^, ^9^, ^10^, ^14^, ^15^ ^]^ The schematic of each classic multi‐stable structures is presented aside as well. C) Demonstration of an impulsive gripper developed in this work. D) Comparison of bi‐stable grippers designed in this work to those in the existing literature.^[^ ^4^, ^8^, ^18^, ^19^, ^20^ ^]^ Bi‐stable grippers are inspired by the natural construction of flytraps, and with excellent merits of self‐locking and impulsive motion. E) Demonstration of a reconfigurable tube developed in this work.

The multi‐stability that could be derived from the structural multi‐compatibility is discovered by a type of multi‐compatible kirigami compliant structure design.^[^ ^3^ ^]^ As a preceding work, a type of 2D multi‐stable truss structures^[^ ^4^ ^]^ is generated by specifically designing this multi‐compatibility. In this paper, we proposed a generalized multi‐compatibility design method for spatial linkages with non‐symmetric and prescriptible stable configurations. We experimentally demonstrated that single‐loop spatial linkages can be transformed into intrinsically multi‐stable spatial (IMSS) linkage, and suggest that all over‐constrained mechanisms can be redesigned into multi‐stable structures. The basic idea is illustrated in **Figure** **1**A: for a general mono‐stable linkage (1), its configuration is determined by the mono‐compatibility. However, if its compatibility is continuous, it turns out to be an over‐constrained linkage mechanism (2) with certain kinematic DoFs, and thus its reconfiguration would be zero‐energy transformation. On the other hand, if the closure condition is satisfied only at the certain discrete configuration on the “kinematic path” (i.e., minimum energy path), the spatial linkage would be a multi‐compatible linkage (3), and these structurally compatible configurations are exactly the stable states of the IMSS linkage. The constructive method by stacking and chaining strategies generates multi‐loop IMSS linkage assemblies (4) of a reconfigurable tube and an impulsive gripper toward practical applications. The essential idea is presented visually in Movie S1 (Supporting Information).

There is a general comparison between IMSS linkages and other previous multi‐stable structures, in terms of the dimension of motion, the part count of links, the number of actuators for each module, the number of redundant stable states, the freedom of selecting the stable state, and the number of designable stable states, as shown in Figure 1(B). Bi‐stable module based on symmetry^[^ ^2^, ^5^ ^]^ is a basic formation of multi‐stable structures. The snap‐through instability^[^ ^6^ ^]^ could guide the reconfiguration between two stable states. However, there are only two symmetric configurations to be stable. Some designs^[^ ^7^ ^]^ based on this symmetry could reach up to tri‐stability through delicate assembling. Similarly, thin‐walled structures ^[^ ^8^, ^9^ ^]^ could maintain their shape in only two spatial configurations due to the within structural properties. Comparably in IMSS linkage modules, the multi‐stability is an intrinsic property, and more asymmetric stable states (>3) could be achieved by adjusting the designable variables itself. Another method is to introduce elastic springs into rigid mechanisms and transform them into multi‐stable structures.^[^ ^10^, ^11^, ^12^ ^]^ Due to the nonlinear kinematic behavior of mechanisms, the attached elastic springs can generate a multi‐stable energy variation during morphing. However, the requirement for the prescription of the prestress of springs makes it complicated to manufacture, increasing its structural complexity. More importantly, the incorporated structures often suffer from the lack of structural stiffness.^[^ ^13^ ^]^ Multi‐stable truss structures^[^ ^4^ ^]^ provide a design principle of multi‐compatibility toward 2D structure modules and assemblies. Another intuitive method to design multi‐stable structures with more complicated reconfiguration is to assemble the multi‐stable modules to multiple loops.^[^ ^2^, ^4^, ^7^, ^14^ ^]^ However, the design based on this method would be restricted by the base modules, and thus a higher complexity of assembling strategy would be involved for more complicated configuration. Moreover, it would result in numerous redundant stable states that distract the desired reconfiguration. Some multi‐stable origami patterns^[^ ^15^, ^16^, ^17^ ^]^ can be seen as an assembly of multiple mechanism modules, yet coupling with discrete structural compatibility. It can also result in a multi‐stability with various configurations, but also faces the issue of redundant stable states.

Specifically, we develop an intrinsically bi‐stable four‐revolute‐joint spatial (IMSS‐R4S2) linkage and an intrinsically quadra‐stable six‐revolute‐joint spatial linkages (IMSS‐R6S4), which are respectively comparable to the corresponding over‐constrained mechanisms of Bennett linkages and Bricard linkages. The advantages over over‐constrained mechanisms include the higher shape accuracy of target configuration, the higher stiffness to preserve the shape, and the impulsivity with fewer energy consumption. The detailed comparison of a Bennett linkage and an IMSS‐R4S2 linkage is elaborated in “4 Quantitative comparison of IMSS linkages and over‐constrained spatial linkages” of Supporting Information. Additionally, the relaxation of the closure condition (from being continuously satisfied to discretely satisfied) enlarges the design space. This allows IMSS linkages to be non‐symmetric, while Bennett linkages and Bricard linkages can only be rather symmetric. (For instance, the links that are diagonal to each other need to have the same length in Bennett linkages.) Then, we couple multiple IMSS linkage modules into a multi‐loop assembly following the coupling strategies of chaining and stacking to generate an impulsive gripper (in Figure 1C) and a reconfigurable tube (in Figure 1E). Only inaccurate linear SMA actuators are needed for these two robotic devices to rapidly reconfigure between the stable states. The comparison of the gripper to the previous robotics devices is presented in Figure 1D.

It should be noted that, the term “linkage” in this paper refers to bars/links connected by joints, and all components are elastically deformable, so multi‐compatibility can result in multi‐stability. The term “rigid mechanism,” or “mechanism” for short, refers to linkages with more than zero kinematic DoF. The term “deformable structure,” or “structure” for short, refers to linkages with no kinematic DoF, i.e., no zero‐energy deformation mode, while it can still morph because elastic deformation of components is permitted.

## 2 Results

This section contains: 1) the design of the single‐loop bi‐stable 4R (IMSS‐R4S2) linkage modules, which gives an example of the inverse design method based on the Denavit–Hartenberg (D‐H) parameterization^[^ ^21^ ^]^ and the multi‐compatibility constraints; 2) the design of single‐loop quadra‐stable 6R (IMSS‐R6S4) linkages, where the approach based on multi‐loop coupling by two IMSS‐R4S2 linkage modules is emphasized, and it suggests that complicated IMSS linkage construction with more linkages could be designed with this multi‐loop coupling approach; 3) two preliminary application cases of multi‐loop IMSS linkages to exhibit the potential for the deployability and impulsivity.

### 2.1 Bi‐Stable Single‐Loop Spatial 4R Linkages

**Figure 2:**
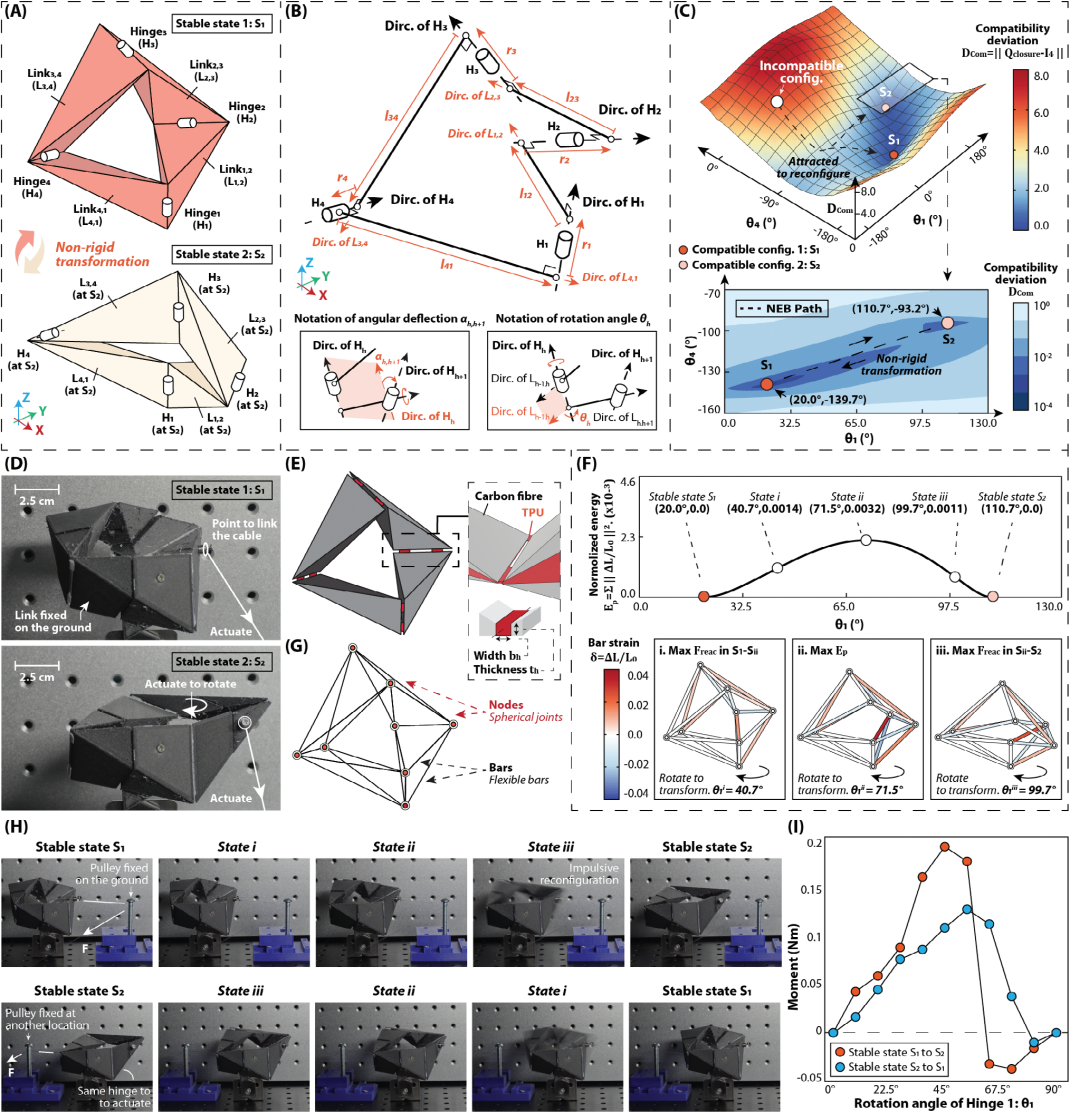
Demonstration of intrinsically bi‐stable 4R spatial (IMSS‐R4S2) linkages. A) Schematic of a bi‐stable 4R spatial linkage. Two stable states are shown in two colors. B) D‐H notation of 4R linkages. All designable parameters are shown in Figure S2C (Supporting Information). Specifically, the angular deflection angle α_*h*, *h* + 1_ and the rotation angles θ are noted. C) Incompatibility landscape of the bi‐stable 4R structure. Two compatible configurations (red and yellow balls) are found at two global minimums of the landscape, which indicates the bi‐stability of the IMSS‐R4S2 linkages. This landscape constructs a domain of attraction where other incompatible configurations would be attracted in the two configurations with the global minimum energy. The zoom‐in figure shows in the logarithmic formation. The path indicated by a dashed line is calculated by the nudged elastic band (NEB) method and shows the reconfiguration with the lowest energy variation. D) Physical model of the IMSS‐R4S2 linkage at two stable states. E) Composition of the physical model of the IMSS‐R4S2 linkage. The integrated approach of 3D printing is conducted to fabricate. The link is made of carbon fibre, while the hinge is made of TPU and inserted between two links. The thickness and the width of hinges are noted as *t*_*h*_ and *bh*. F) The simulated energy variation and deformation are generated by replacing tetrahedrons with pin‐jointed bars as shown in (G), and are calculated by the NEB method. At the intermediate states, the elongation of the bars is noted by colors. H) Process of the reconfiguration between two stable states. The reconfiguration is actuated by a cable attached to the hinge *H*_1_, as shown in (D). The whole process of reconfiguration is included in Movie S2 (Supporting Information). I) Moment‐to‐rotation‐angle curve. The experimental setup is given in Figure S7 (Supporting Information). Bi‐stability is observed based on the curve.

The basic idea is shown in this section through a single‐loop bi‐stable 4R linkage (IMSS‐R4S2) with two prescriptible stable states, as in **Figure** **2**A. For a closed‐loop linkage, the closure condition holds to preserve its geometric configuration. For instance, Bennett linkage is a particular over‐constrained type of 4R spatial linkages,^[^ ^22^ ^]^ where the closure condition is satisfied for any configurations in the potential configuration space of 4R linkages, and thus, one kinematic degree of freedom is obtained to make it a mechanism.

However, the multi‐compatibility principle^[^ ^4^ ^]^ of multi‐stable structures indicates that, if the closure condition is imposed merely on the discrete and finite configurations, the structural compatibility of the linkage mechanism would be satisfied only at these states, and thus, the linkage mechanism turns into a multi‐stable linkage structure with the prescribed stable states, rather than an over‐constrained mechanism.

(1) $$\begin{array}{ccc} Q_{\text{closure}} & = & T_{1,2}^{s}\left(\theta^{s}\right)\cdot T_{2,3}^{s}\left(\theta^{s}\right)\cdot T_{3,4}^{s}\left(\theta^{s}\right)\cdot T_{4,1}^{s}\left(\theta^{s}\right)=I_{4}.\\ & & \left\{\begin{array}{ccc} \forall s\in \Omega_{\mathrm{Config}.} & \to & \text{Over-constrained mechanisms}\\ s\in S_{1},\text{…},S_{n} & \to & \text{Multi-compatible structures} \end{array}\right. \end{array}$$

where $T_{i,j}^{s}\left(\theta^{s}\right)$ is the transformation matrix from hinge *h*_*i*_ to hinge *h*_*j*_ at the configuration *s*, while the potential configuration space of the 4R linkage is expressed as Ω_*Config*._. Multi‐compatible structures are equivalent to multi‐stable structures, due to the permission of elastic deformation within the linkage.

An inverse design approach for IMSS‐R4S2 linkages is, therefore, able to be set up by the constrained optimization solver, as indicated in Figure 6 and Section 3. The constraints are that the closure conditions should be satisfied at the prescribed multiple configurations, as the multi‐compatibility principle indicated. In this introductory example, the multi‐stability is assigned as ${\hat{\theta }}_{1}^{S_{1}}=110.0^{\circ },{\hat{\theta }}_{1}^{S_{2}}=20.0^{\circ }$, and the design result is shown in Figure 2A with the rotation angles of $\theta_{1}^{S_{1}}=110.7^{\circ },{\hat{\theta }}_{1}^{S_{2}}=20.0^{\circ }$. The design error in this case is computed as: $\delta_{\theta h}\left(\theta_{\theta h}^{\text{S1}},\text{…},\theta_{\theta h}^{\text{Sn}}\right)=1/n\cdot \sum |\theta_{h}^{s}-{\hat{\theta }}_{h}^{s}|=\delta_{\theta 1}^{\text{4R}}\left(110.7^{\circ },20.0^{\circ }\right)=0.35^{\circ }$ More computing details about the IMSS‐R4S2 linkage case could be found in “2.1 Design details of IMSS‐R4S2 linkage” of Supporting Information, and the code to generate the case is included in Data S1 (Supporting Information). It is noted that the stable configurations are not generally symmetric or planar, which suggests it expands to an new design space over other multi‐stable unit cells. More exploration regarding the design space of the inverse design approach is discussed in “5 Stiffness analysis for IMSS‐R4S2 linkage modules and alternative designs” of Supporting Information, where the design deviation and the structural stiffness are evaluated with various angular target inputs.

The multi‐compatibility principle for close‐loop linkages is further validated by incompatibility landscape as in shown in Figure 2C. When controlling two rotation angles θ_1_ and θ_4_, other two rotation angles θ_2_ and θ_3_ are calculated with the objectives of minimizing the incompatibility of this 4R linkage. The incompatibility is evaluated by the deviation of the closure matrix, D_Com_ = ‖**Q**_closure_ − **I**_4_‖. Intuitively from the contour, there are only two states with a global minimum of incompatibility in the whole range of the landscape, which suggests there are only two structurally compatible states, i.e., the stable states. It also shows the domain of attraction of multi‐stable structures. Other configurations away from the two stable states would be attracted by the structural incompatibility, and then be guided to “fall” toward into the prescribed stable states even with a simple actuator roughly directing to these two states. The code to conduct the analysis is in Data S2 (Supporting Information).

The physical model at two stable states is presented in Figure 2D, which is free from the issue of self‐interference by the hinges sliding strategy proposed in “5 Stiffness analysis for IMSS linkage modules and alternative designs” of Supporting Information, as shown in Figure 2E. Physically, the links are made of carbon fibre to provide the sufficient strength, and hinges are made of TPU to provide the smooth rotation and meanwhile the most of deformation. The properties of these two materials are presented in Table S1 (Supporting Information). The relation between the stiffness of the linkages and the connection strength of hinges (characterized by the thickness *t*_*h*_ and the width *b*_*h*_ of hinges, as defined in Figure 2E), is quantitatively compared as in Figure S9D (Supporting Information). The stiffness of the linkage is characterized by the maximum force in the pulling test. The video for the test is included in Movie S2 (Supporting Information). Further analysis of the relation between the stiffness and the connection strength is included in “2.1 Design details of IMSS‐R4S2 linkage” of Supporting Information.

The simulated energy variation in Figure 2F is conducted by the nudged elastic band (NEB) method,^[^ ^23^, ^24^ ^]^ which is explained in “1.3.1 Deformation analysis for IMSS linkages” of Supporting Information. Under the assumption of the truss model, link tetrahedrons are replaced with pin‐jointed bars theoretically, as shown in Figure 2G. Three intermediate states (State i, State ii, and State iii) are characterized specifically to show the deformation of each bar within the truss model. Physically, the actuation is implemented by rotating Hinge 1 with an offset pulling cable, which is through a fixed pulley and then attached on Link_1, 2_, as shown in Figure 2D. The actuating process from Stable state *S*_1_ to Stable state *S*_2_ and the reverse process from Stable state *S*_2_ to Stable state *S*_1_ are presented in Figure 2H respectively, with the different layout yet to actuate the same rotation angle θ_1_ of Hinge *H*_1_. It is necessary to reverse the actuating force. The impulsive motion is observed at ${\text{State}}_{iii}^{S1-S2}$ and ${\text{State}}_{i}^{S2-S1}$. It is further validated by the curve of moment variation on Hinge H_1_ along the reconfiguration, shown in Figure 2I. The bending experiment is set up as instructed in “1.3 Validation block” of Supporting Information. It is noted that it is possible to complete the reconfiguration by more compact actuators, such as shape memory alloy (SMA) actuators. An example of using an SMA actuator to reconfigure from Stable state *S*_1_ to Stable state *S*_2_ is presented in Movie S2 (Supporting Information).

### 2.2 Quadra‐Stable Single‐Loop Spatial 6R Linkages

As a general design approach, the inverse design framework is also applicable to generating intrinsically multi‐stable spatial linkages of single‐loop 6R, which is presented in “2.3 Tri‐stable 6R (IMSS‐R6S3) linkages” of Supporting Information. Another design approach is emphasized in this section for IMSS‐R6S4 linkages. It provides a more intuitive generative way by coupling two IMSS‐R4S2 linkages, and therefore it is able to generate a spatial 6R linkage with more intrinsic stability and less self‐interference, which is frequently appeared in IMSS‐R6S3 linkages designed by the inverse design framework.

**Figure 3:**
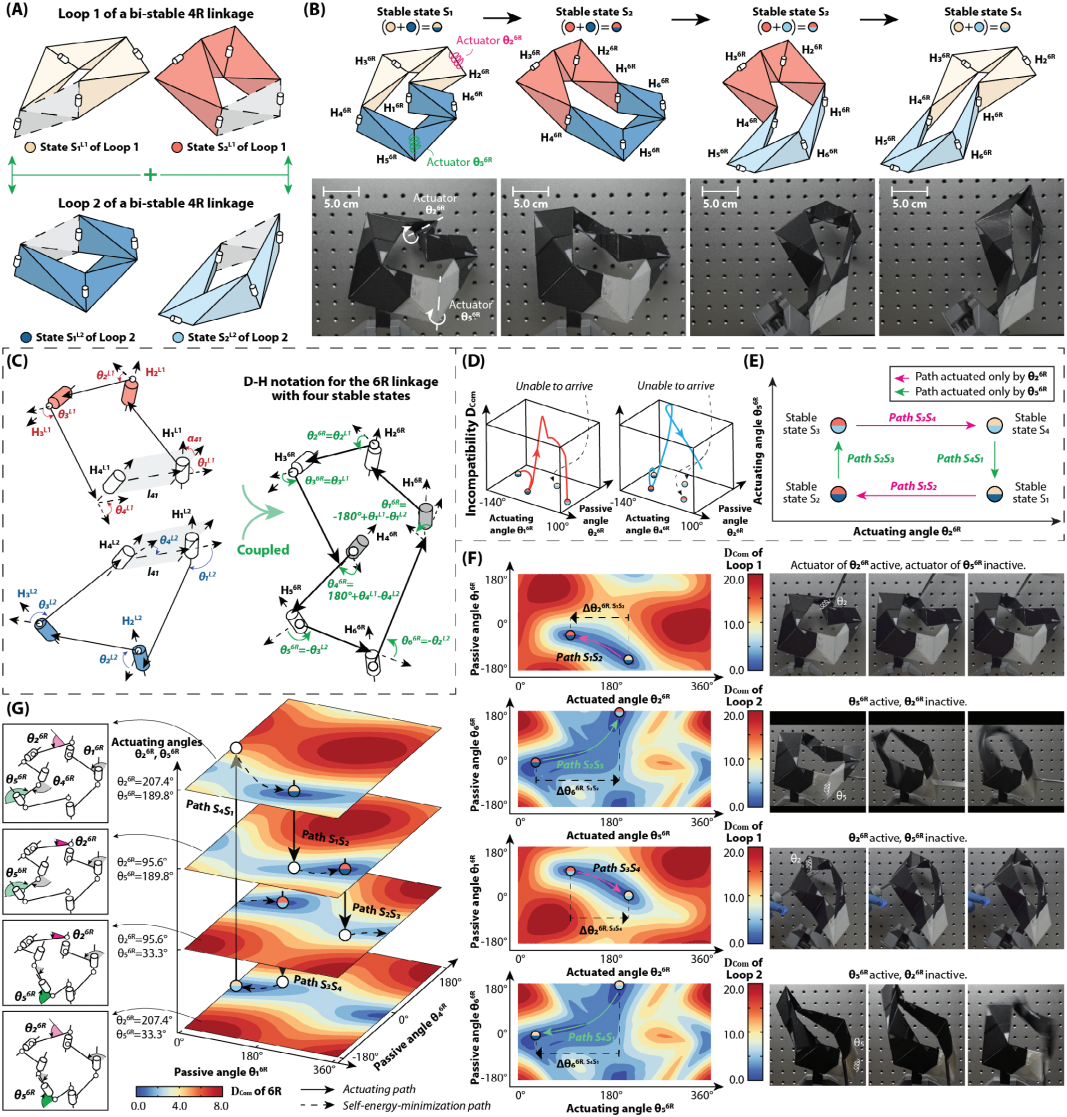
Demonstration of intrinsically quadra‐stable 6R spatial (IMSS‐R6S4) linkages. (A) Design approach based on multi‐loop coupling by two bi‐stable 4R spatial linkages. The links in gray are dropped as the two hinges at the end of the link are coupled. (B) Four stable states of IMSS‐R6S4. The reconfiguration of the upper part is inherited from Loop 1 IMSS‐R4S2 linkage, while the lower part is from Loop 2 IMSS‐R4S2 linkage. Two actuators are implemented to control the rotation angles of Hinge 2 (θ_2_) and Hinge 5 (θ_5_) in the IMSS‐R6S4 linkage. (C) D‐H notation of two IMSS‐R4S2 linkages and the coupled IMSS‐R6S4 linkage. (D) Equilibrium path when merely actuating one angle ($\theta_{1}^{\text{6R}}$ and $\theta_{4}^{\text{6R}}$ respectively). In the situation when actuating $\theta_{1}^{\text{6R}}$ (left), Stable state *S*_4_ is not available in the reconfiguration path, while Stable state *S*_3_ and Stable state *S*_4_ are not available when actuating $\theta_{4}^{\text{6R}}$ (right). Other paths are presented in Supplementary code S2, and they indicate a similar phenomenon that some stable states are not available. It suggests that it is impossible to complete the reconfiguration among all stable states with only one angular actuator. (E) Reconfiguration path among all stable states when two rotation angles θ_2_, θ_5_ are actuated. (F) Incompatibility landscape along each reconfiguration sub‐path (Path *S*_1_ *S*_2_, Path *S*_2_ *S*_3_, Path *S*_3_ *S*_4_, and Path *S*_4_ *S*_1_ respectively). It is under the hypothesis that the incompatibility of the IMSS‐R6S4 linkage only comes from the compatibility deviation of the sub‐loop of IMSS‐R4S2 where the actuator is active. For instance, θ_2_ is actuated to increase along Path *S*_1_ *S*_2_, and therefore the deviation of closure condition in the IMSS‐R4S2 Loop 1 linkage could be representative of the incompatibility of the IMSS‐R6S4 linkage. The processes could be actuated by SMA materials and cable, as shown in Section S4.3 (Supporting Information). The whole process of reconfiguration is included in Movie S3 (Supporting Information). (G) An overall reconfiguration path actuated by two rotation angles. From Stable state 1 to Stable state 2, the incompatibility landscape is changed due to the regulation of θ_2_ and θ_5_, which then makes the configuration at Stable state 1 no longer compatible after the regulation. By the effect of the attraction domain in the incompatibility landscape, it would then be automatically fell into the global minimum of this new landscape, which is exactly Stable state 2.

As shown in **Figure** **3**A, two loops of IMSS‐R4S2 linkages are presented at their two stable states respectively. Coupling them along the hinges that are connected by the gray link, and a single‐loop 6R with four stable states (IMSS‐R6S4 linkage) is generated as in Figure 3B, where each stable state in the IMSS‐R6S4 linkage is a combination by the stable states of two IMSS‐R4S2 linkages. The coupling condition is that the relative location of the coupled hinges should be consistent in two IMSS‐R4S2 linkages, as shown in Figure 3C:

(2) $$\left\{\begin{array}{c} l_{41}^{\text{Loop 1}}=l_{41}^{\text{Loop 2}}\\ \alpha_{41}^{\text{Loop 1}}=\alpha_{41}^{\text{Loop 2}} \end{array}\right.$$

The D‐H parameters in the IMSS‐R6S4 linkage are assigned with that are originally in each IMSS‐R4S2 linkage loop, as in Figure 3C. The rotation angles in the new 6R linkage are derived as follows. More design detail could be found in “2.3 Design details of IMSS‐R6S4 linkage” of Supporting Information. The code to design based on the multi‐loop coupling is included in Data S1 (Supporting Information).

(3) $$\left\{\begin{array}{c} \theta_{1}^{\text{6R}}=\theta_{1}^{\text{L1}}-\theta_{1}^{\text{L2}}-180^{\circ }\\ \theta_{2}^{\text{6R}}=\theta_{2}^{\text{L1}},\theta_{3}^{\text{6R}}=\theta_{3}^{\text{L1}}\\ \theta_{4}^{\text{6R}}=\theta_{4}^{\text{L1}}-\theta_{4}^{\text{L2}}+180^{\circ }\\ \theta_{5}^{\text{6R}}=-\theta_{3}^{\text{L2}},\theta_{6}^{\text{6R}}=-\theta_{2}^{\text{L2}} \end{array}\right.$$

There would be a reconfiguration issue for the IMSS‐R6S4 linkage when there is only one actuator. As shown in Figure 3D, when actuating one angle, $\theta_{1}^{\text{6R}}$, in the IMSS‐R6S4 linkage reconfiguring from Stable state *S*_1_ to other stable states, other angles are changed passively. It indicates that the reconfiguration path could pass through Stable state *S*_1_, *S*_2_, and *S*_3_, while Stable state *S*_4_ is available at the reconfiguration path. Therefore, there are only three stable states (*S*_1_, *S*_2_, and *S*_3_) if only actuating $\theta_{1}^{\text{6R}}$. A similar phenomenon is observed in another reconfiguration path when actuating $\theta_{4}^{\text{6R}}$, where there are only two stable states (*S*_1_, *S*_2_). All reconfiguration paths when actuating one rotation angle are included in Code S2 (Supporting Information). Collectively, it suggests it would be impossible to complete the reconfiguration among all stable states with only one angular actuator.

An intuitive actuating strategy is shown in Figure 3E that the reconfiguration could be completed by at least two actuators. Two actuators are implemented on Hinge 2 and Hinge 5 as in Figure 3B, which control the reconfiguration of the hinges inherited from the IMSS‐R4S2 Loop 1 linkage ($\theta_{2}^{\text{6R}}$) and Loop 2 linkage ($\theta_{5}^{\text{6R}}$), respectively. For instance, the reconfiguration from Stable state 1 to Stable state 2, which is equivalently the reconfiguration of the IMSS‐R4S2 Loop 1 linkage, could be achieved by only regulating $\theta_{2}^{\text{6R}}$, as Path *S*_1_ *S*_2_ in Figure 3E. Similarly, Path *S*_2_ *S*_3_, which is the reconfiguration of the IMSS‐R4S2 Loop 2 linkage, could be achieved by only regulating $\theta_{5}^{\text{6R}}$ with the corresponding actuator. The process of each path is physically presented in Figure 3F and Movie S3 (Supporting Information). The incompatibility landscape linkage along each path is shown aside to indicate the accessibility of the actuating strategy. Note that the analysis is under an assumption that the incompatibility in the IMSS‐R6S4 is only contributed by one loop of the IMSS‐R4S2 linkage component at each reconfiguration path, and there is no deformation in the other linkage component (such as Loop 2 in Path *S*_1_ *S*_2_).

An overall reconfiguration path of the IMSS‐R6S4 linkage actuated by the two rotation angles, $\theta_{2}^{\text{6R}}$ and $\theta_{5}^{\text{6R}}$, is shown in Figure 3G. By regulating these two angles, the incompatibility landscape would be changed at a different layer. The reconfiguration path between each layer is a equilibrium path where each configuration on the path is the local minimum of the incompatibility landscape in this layer. If there is a global minimum of the incompatibility landscape as $\theta_{2}^{\text{6R}}$ and $\theta_{5}^{\text{6R}}$ regulating, the IMSS‐R6S4 would then arrive at the next structurally compatible state, i.e., stable state.

For instance, at first, the IMSS‐R6S1 linkage is at Stable state *S*_1_, where $\theta_{2}^{\text{6R}}=207.4^{\circ },\theta_{5}^{\text{6R}}=189.8^{\circ }$, and the configuration of the linkage is structurally compatible $D_{\text{com}}^{S1}=0$. Then activate the actuator of $\theta_{2}^{\text{6R}}$, and decrease $\theta_{2}^{\text{6R}}$ from 207.4°, yet keep $\theta_{5}^{\text{6R}}$ unchanged (the actuator of $\theta_{5}^{\text{6R}}$ is inactive), the incompatibility landscape of the IMSS‐R6S4 would be changed from layer 1 to another layer. The configuration would turn out to be incompatible ($D_{\text{com}}^{s}>0$), and then be attracted to a new state that is a local minimum in the incompatibility landscape of this layer. Continue decreasing the angle to layer 2 where $\theta_{2}^{\text{6R}}=95.6^{\circ }$, and $\theta_{5}^{\text{6R}}$ still keeps the same ($\theta_{5}^{\text{6R}}=189.8^{\circ }$), and there would be a global minimum in this incompatibility landscape, and the IMSS‐R6S4 could arrive this structurally compatible configuration ($D_{\text{com}}^{S2}=0$), which suggests the linkage are reconfigured to another stable state. It is noted that the actuated angle would not necessary to be regulated exactly at $\theta_{2}^{\text{6R}}=95.6^{\circ },\theta_{5}^{\text{6R}}=189.8^{\circ }$. It is the right direction of increasing or decreasing the angle that matters. The domain of attraction that is constructed by the incompatibility would help find the next global minimum.

The code to analyze the incompatibility landscape is included in Data S2 (Supporting Information). Besides the method based on multi‐loop coupling, the inverse design method could also be capable of generating multi‐stable 6R linkages with prescriptable stable states. A design case of an IMSS‐R6S3 linkage is presented in “2.3 Design details of IMSS‐R6S3 linkage” of Supporting Information, which is a direct output of the inverse design method. It is validated that more flexible design space is achieved with more designable parameters involved, compared to that of IMSS‐R4S2 linkages, while a higher design error and lower stiffness is observed in the IMSS‐R6S3 linkages because of the dispersion of incompatibility on more configurations.

### 2.3 Preliminary Applications of Multi‐Loop IMSS‐R4 Linkage Assembly

Coupling single‐loop IMSS linkage modules into a multi‐loop assembly is an effective route to extend its application range. No additional DoF is introduced as assembling, and thus, all IMSS linkage modules in the multi‐stable assembly are able to reconfigure simultaneously and collaboratively. (See “1.2.5 Coupling strategy of IMSL linkages with multiple loops” in Supporting Information). The coupling conditions regarding the rotation angle variation of the coupled hinges are imposed mathematically as:

(4) $$\Delta \theta_{hj}^{L_{i}}={\left(-1\right)}^{C}\cdot \Delta \theta_{hk}^{L_{i+1}}$$

where $\Delta \theta_{hj}^{L_{i}}$ and $\Delta \theta_{hk}^{L_{i+1}}$ refer to the variation of the rotation angle of the coupled hinges *h*_*j*_ and *h*_*k*_ in the adjacent single‐loop modules L_*i*_ and L_*i* + 1_, and *C* characterizes the chirality of the coupled hinges, corresponding to the IMSS linkage assembly of stacking and of chaining respectively. The coupling conditions are implemented physically by coupling frames, which fasten the coupled modules with no relative movement.

**Figure 4:**
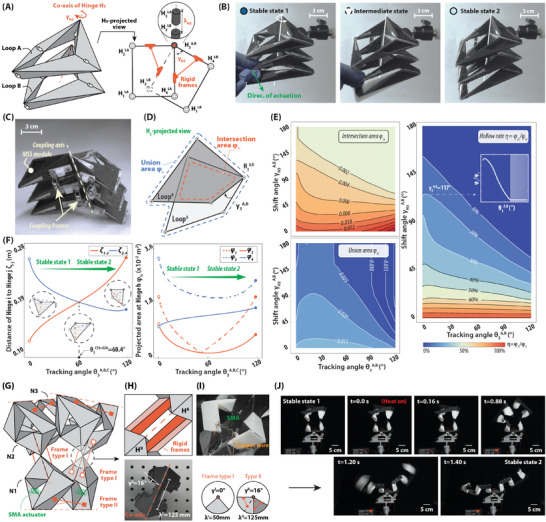
Demonstration of stacking coupling of IMSS linkage modules: a deployable tube. A) Stacking coupling strategy. Coupled hinges are placed co‐axis, while coupling frames (in orange) are fixed on the adjacent tetrahedrons. The deflection angle $\gamma_{3}^{A,B}$ and deflection interval $\lambda_{3}^{A,B}$ at coupled hinge H_3_ are defined. B) Snapshots of reconfiguration of the physical model of 3‐module IMSS assembly. Simultaneous and collaborative reconfiguration motion is observed. C) Details of coupling frames in the physical model. The coupled hinges of three modules are co‐axis and fixed by rigid frames with each others. D) Definition of the intersection area ϕ_∧_ and the union area ϕ_∨_ of this tube with the changeable section area. The hollow rate η(γ_3_) = ϕ_∧_/ϕ_∨_, is defined to characterize the ability to transmit the fluid through. E) Analysis of the hollow rate to the deflection angle γ_3_. When γ_3_ = 117°, the hollow rate rises slightly before the reduction at the reconfiguration, as shown in the minor panel. F) Projected shape analysis of the stacking IMSS linkage assembly. The arc length ζ_1 − 3_, ζ_2 − 4_ and the projected area ϕ_*h*_ to hinge H_*h*_ is variated with rotation angle $\theta_{3}^{A,B,C}$. G) Schematic of another deployable tube, with symmetrical 2 × *N* = 6 modules. There are two types of coupling frames. A pair of SMA actuators is placed at the hinge between two N1 modules to provide sufficient force to actuate the rotation angle. H) Physical model of Type II coupled hinges. I) Physical circuit of one SMA actuator. J) The process of reconfiguration is actuated by an SMA actuator. The whole process of reconfiguration is included in Movie S4 (Supporting Information). The simulation of the construction with a large number of loops is in “3.2 Deployable tube with designable deployment ratio” of Supporting Information.

An assembly made of three identical stacking single‐loop IMSS linkage modules Loop^A^, Loop^B^, Loop^C^ is presented in **Figure** **4**A for the basic idea of stacking coupling assembly. The coupling condition for stacking is therefore expressed as:

(5) $$\Delta \theta_{3}^{LA}=\Delta \theta_{3}^{LB}=\Delta \theta_{3}^{LC}$$

which is implemented by the co‐axis aligned modules and the rigid coupling frames between them. The designable parameters of the deflection angle γ_3_ and the deflection interval λ_3_ of coupled hinge H_3_ are chosen based on the practical requirements, where it is set as $\gamma_{3}^{A,B}=\gamma_{3}^{B,C}=0$ and $\lambda_{3}^{A,B}=\lambda_{3}^{B,C}=0.3$ m in this case in Figure 4B. The stability is preserved the same as the IMSS linkage modules, and it is observed as well that the reconfiguration could be transmitted from one module to the whole assembly simultaneously and collaboratively. The physical model of the stacking tube is shown in Figure 4C. It is indicated in Figure 4F that the arc length ζ_1 − 3_, ζ_2 − 4_ of hinges H_1_ − H_3_ and H_2_ − H_4_ expresses the opposite trend, which suggests the shape of the section comes from wide to narrow. The deflection angle γ_3_ of the adjacent modules is utilized to customize the hollow rate η(γ_3_), as in Figure 4D, which is defined as:

(6) $$\eta \left(\gamma_{3}\right)=\frac{\phi_{\wedge }}{\phi_{\vee }}$$

where ϕ_∧_ is the intersection area of the projected section area of the stacking modules and ϕ_∨_ is the union area, as shown in the Figure 4D. Considering a tube with two identical IMSS linkage modules Loop^A^, Loop^B^, the variation of the hollow rate along the transformation of the tube is shown in Figure 4E. Design details are included in “3.1 Three‐module reconfigurable tube with tunable section area” of Supporting Information.

Based on this previous validation, a multi‐loop strategy to design another deployable tube with more IMSS linkage modules is shown in Figure 4F. There are 2 × *N* = 6 modules placed symmetrically in the tube, and two types of coupling frames to construct the whole structure, where γ^I^ = 0°, λ^I^ = 50 mm in Type I, and γ^II^ = 16°, λ^II^ = 125 mm in Type II, as shown in Figure 4G.

An SMA actuator is implemented on the hinge between two N1 modules as indicated in Figure 4H. In practice, there are two sets of actuators placed at the hinge to provide sufficient contraction force to close this single hinge, and then actuate the whole tube to deploy. The process of the reconfiguration is shown in Figure 4I and Movie S4 (Supporting Information). It should be noted that the layout of actuators should be deliberately designed for better transmissibility of local reconfiguration in IMSS linkage assemblies, especially if there are many more modules to be coupled. It would be feasible to place the actuators close to the center of the entire device as in this case. For the worst scenario, there could be one actuator for each IMSS module in the assembly. More details about the target configuration of this N3‐module deployable tube is included in “3.2 Deployable tube with designable configurations” of Supporting Information.

**Figure 5:**
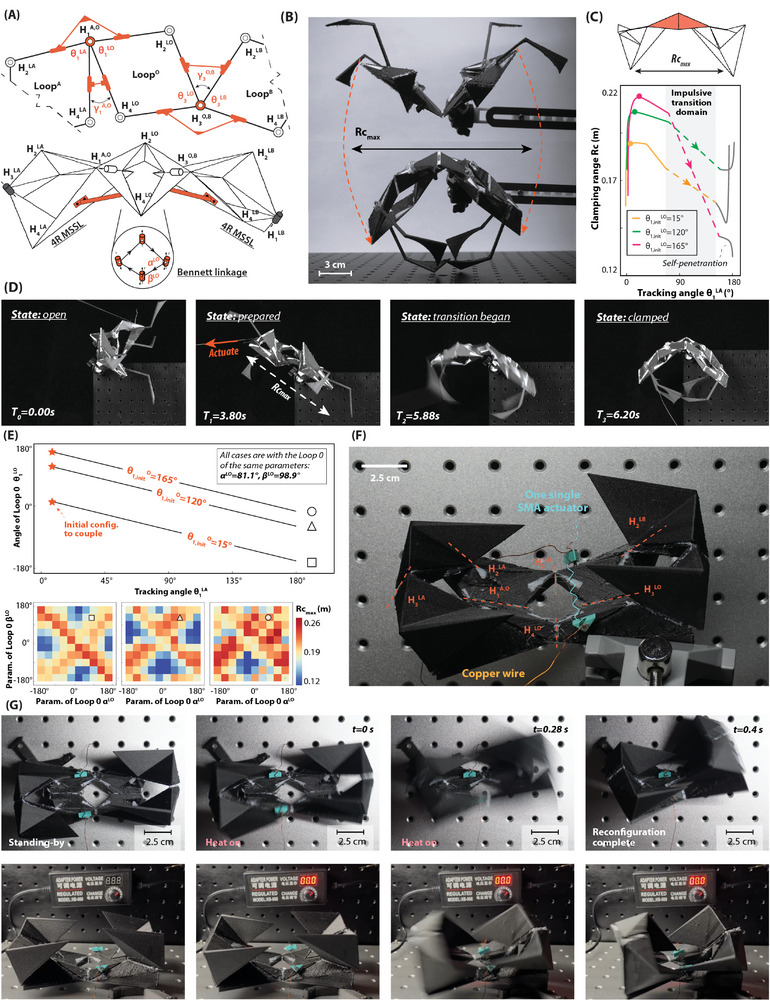
Demonstration of chaining coupling of IMSS linkage modules: an impulsive gripper. A) Chaining coupling strategy. The deflection parameters $\gamma_{1}^{A,O}$, $\gamma_{1}^{O,B}$ and $\lambda_{3}^{A,O}$, $\lambda_{3}^{O,B}$ are defined following the stacking coupling. The initial installation angle $\theta_{1,\text{init}}^{LO}$ of Loop^*O*^ shows the starting angle at the installation state. B) Clamping trajectory of the gripper. The maximum of the clamping range Rc_*max*_ is defined by its trajectory as the reconfiguration when actuating. C) Clamping simulation of the gripper designs with different initial installation angles $\theta_{\text{init}}^{LO}=15^{\circ },120^{\circ },165^{\circ }$. D) Actuating experiment by cable. The reconfiguration from the state of preparing (T_1_) to the state of transition beginning (T_2_) takes 2.08 s, and it takes 0.32 s to jump to the clamped state (T_3_). The whole process is shown in Movie S5 (Supporting Information). E) Analysis of the clamping range to the initial installation angle and the structural parameters. F) An more compact gripper with three module coupled. One single SMA actuator is placed at two links of the center Bennett linkage for better actuation. G) Snapshots of the impulsive clamping motion. The rapid reconfiguration lasts for less than 0.16 s. The whole process of reconfiguration is included in Movie S5 (Supporting Information).

An impulsive gripper with three single‐loop modules is shown in **Figure** **5**A to demonstrate the basic idea of chaining coupling. A type of Bennett linkage (${\text{Loop}}^{O}$) is placed at the center and two identical IMSS linkage modules (Loop^A^ and Loop^B^) are at the end with the merged coupled hinges $H_{1}^{A,O}$ and $H_{3}^{O,B}$ marked in the figure. The coupling condition in chaining coupling is thus denoted by:

(7) $$\Delta \theta_{1}^{LO}=-\Delta \theta_{1}^{LA},\Delta \theta_{3}^{LO}=-\Delta \theta_{3}^{LB}$$

The Bennett linkage of ${\text{Loop}}^{O}$, which is parameterized by the D‐H notation and the structural parameters of the deflection angle of hinges α^L*O*^, β^L*O*^ are defined in the minor panel of Figure 5A, provides two advantages in this design of grippers: 1) better transmission of the reconfiguration from IMSS linkage modules. The continuous kinematic path of Bennett linkages promises that the non‐rigid transformation of IMSS linkage modules can pass through it to another end and trigger the overall reconfiguration; 2) more flexible configuration space to be compatible with IMSS linkage modules, which suggests there would be more choices of configurations of the Bennett linkage to couple with other two IMSS linkage modules under the coupling conditions.

The designable variables of coupling, deflection angle γ_H_ and interval λ_H_.(H ∈ {1, 3}), in the chaining coupling, are similarly defined as those in the stacking coupling. Yet, there is an additional group of variables, the initial installation rotation angle $\theta_{1,\text{init}}^{LO}$, $\theta_{3,\text{init}}^{LO}$, to determine the initial configuration of the Bennett linkage as coupling with IMSS linkage modules. The initial installation rotation angle is associated with the maximum clamping range of grippers, Rc_*max*_, which is defined in Figure 5B. Three gripper design with different $\theta_{1,\text{init}}^{LO}=15^{\circ },120^{\circ },165^{\circ }$ are compared in Figure 5C. As the reconfiguration of grasping continues, the clamping range *Rc* would be increased rapidly, and then decreased impulsively. It is visually validated by the snapshots of the experiments as in Figure 5D. The movie of the whole reconfiguration is included in Movie S5 (Supporting Information). Parametric study to the analysis of the clamping range as tuning the initial installation angles $\theta_{1,\text{init}}^{LO}=\theta_{3,\text{init}}^{LO}=\theta_{\text{init}}^{LO}$ and the structural parameters α^L*O*^ and β^L*O*^ of ${\text{Loop}}^{O}$ is indicated in Figure 5E. When selecting different installation angles $\theta_{\text{init}}^{LO}=15^{\circ },120^{\circ },165^{\circ }$, the Bennett linkage is consistently able to couple with the IMSS linkage modules in the cases at a different range of angular variation. Yet, the angular variation of the coupled hinges:

(8) $$\Delta \theta^{LO}=\theta_{S2}^{LO}-\theta_{S1}^{LO}=\theta_{S2}^{LO}-\theta_{\text{init}}^{LO}$$

stays the same and is obliged to be equal to the angular variation of the coupled IMSS linkage modules, θ^L*O*^ = θ^L*A*^ = θ^L*B*^, as required by the coupling condition. It is observed as well that Rc_max_ is increasing with the initial installation angle due to the larger accessible domain at its initial state of standing by. When selecting different structural parameters additionally, the variation of maximum clamping range is presented respectively at the right. As the increase of the initial rotation angle, the maximum clamping range Rc_max_ increases on average among the cases with the different designable parameters α^L*O*^ and β^L*O*^ of Bennett linkage due to the increase of flipping. The clamping range reaches a higher value in the neighbor where α^L*O*^ = β^L*O*^ or α^L*O*^ = −β^L*O*^, which suggests the wider clamping range is induced by the greater interval of the structural rotated angles in the Bennett linkage. The code of analysis is in Data S2 (Supporting Information).

An alternative model with more compact construction is shown in Figure 5F. The layout is three modules is consistent with that in Figure 5A, yet the applicability is enhanced by its compactness. An SMA actuator on two links of the center ${\text{Loop}}^{O}$ Bennett linkage is retracted to actuate the reconfiguration of the gripper from the open state to the clamp state impulsively. The snapshots of clamping are presented in Figure 5G, where the rapid reconfiguration lasts for less than 0.16 s. The whole process of reconfiguration is included in Movie S5 (Supporting Information). The comparison between two grippers consisting of two IMSS linkages and one Bennett linkage (such as in this case), and consisting of three Bennett linkages (which is thus with 1‐DoF) is discussed in “4 Quantitative comparison of IMSS linkages and over‐constrained spatial linkages” of Supporting Information.

## 3 Methods

**Figure 6:**
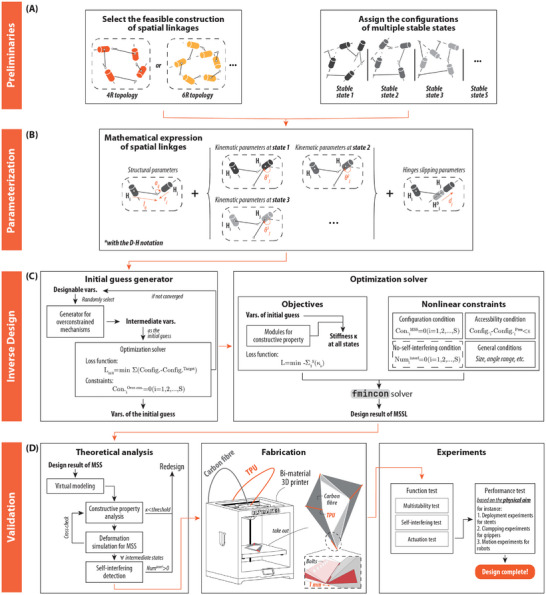
Design framework for intrinsically multi‐stable spatial linkages. A) Assignment block. The feasible formation and the prescribed methods are specified in this block. B) Parameterization block. The spatial linkage is expressed with the D‐H notation and three types of designable parameters. C) Inverse design block. The initial guess of the over‐constrained mechanism closed to the prescribed states is generated. The core optimization solver is constructed with the objectives and constraints. D) Validation block. The design is validated theoretically and experimentally. The physical model is fabricated by the bi‐material 3D printer of UltiMaker S5.

The inverse design framework for intrinsically multi‐stable spatial linkages is presented and discussed in this section. As shown in the **Figure** **6**, the design framework for intrinsically multi‐stable spatial (IMSS) linkages is divided into four main parts: the assignment block, the parameterization block, the inverse design block, and the validation block.

The physical requirement is abstracted as a type of IMSS linkage of a single loop or its assembling and then the topology and the stable configurations are determined, as shown in Figure 6A, which are parameterized mathematically at the next step in Figure 6B by the D‐H notation as a series of independent parameters of structure, kinematic and the hinges sliding. These groups of parameters act as the designable variables for the inverse design solver in Figure 6C, which includes the initial guess generator and the optimization solver, to generate a feasible solution of IMSS linkages then. The initial guess generator is a program to provide the suitable solution of over‐constrained mechanism that is as closed to all the prescribed stable states as much. The optimization solver is based on the “fmincon” function in MATLAB with the objectives defined as to maximum the constructive property and the constrained including the configuration condition, the accessibility condition and no‐self‐interference condition and others.

As in Figure 6D, the solution after this optimization unit is checked for its stiffness and the parts of self‐interference before the fabrication. Note that the deformation simulation is placed at the step after the inverse design out of the consideration to the computing efficiency, or otherwise it ought to be in the constraints in the last optimization solver for the direct solution free from the self‐interference problems. Thus, the redesign occurs if the post‐check fails, or moves to the fabrication if passes. It is implemented by a bi‐material 3D printer of Ultimaker with the materials of carbon fiber (PolyMide^*TM*^ PA12‐CF) and TPU (PolyFlex^*TM*^TPU95), the essential properties of which are listed in Table S1 (Supporting Information). To construct a reliable hinge with flexibility yet high strength, a sandwich‐like structure is proposed where the rotation hinge is made of TPU and fastened to the carbon fiber with the bolts on the both sides as shown.

The final design is validated by the experiments of function tests, which contain the check for the property of the multi‐stability and the interference, and the performance test based on the original physical aims. Each blocks would be elaborated in “1 Details of each blocks in the inverse design framework of IMSS linkages” of Supporting Information.

## 4 Discussion

In summary, we have presented a fundamental design method for intrinsically multi‐stable spatial linkages with prescriptible stable configurations. It is derived from over‐constrained linkages with relaxed compatibility constraints, which is satisfying the closure conditions only at prescribed/discrete states. This accordingly results in a larger design space with more achievable configurations due to the reduction of constraints. As a series of publications that are aimed to explicitly explain the generalization of the multi‐stable structures design method based on the multi‐compatibility principle,^[^ ^4^, ^25^ ^]^ this paper focuses on the design based on the spatial linkages, and therefore presents the transformation of a Bennett linkage into a bi‐stable 4R linkage, a tri‐stable 6R linkage transformed from a Bricard linkage (briefly), and a quadra‐stable 6R linkages. Moreover, this design method is potentially applicable for other formation of over‐constrained construction, such as origami and kirigami,^[^ ^12^, ^17^, ^26^ ^]^ and other highly over‐constrained construction like flexible frame structures^[^ ^27^ ^]^ (which could be seen as a continuous structure of no specific hinges, yet possibly with multiple symmetric structural compatibility, like hair clips), or recently‐discovered over‐constrained mechanisms,^[^ ^28^ ^]^ to be transformed into multi‐stable structures with larger design space. The multi‐loop coupling of IMSS linkages illustrated in the paper lays the foundation for practical applications. Stacking and chaining couplings of IMSS‐R4S2 linkages are demonstrated for conceptual and preliminary applications like reconfigurable tubes, and impulsive grippers. It illustrates a way to couple single‐loop IMSS linkages into a multi‐loop assembly with internally coordinated multi‐stability. Other than the single‐to‐multi‐loop construction strategy, it is also feasible to design the multi‐loop IMSS linkage directly, which can be by parameterizing a multi‐loop and generically immovable linkage and feeding the design parameters to the objective function and multi‐compatibility constraints, but significantly more computational time is expected.

There are several limitations in the paper that need to be noted. The actuating issue would be more challenging if more loops of modules coupling into an assembly. There are two aspects of future work to deal with it: 1) the stiffness distribution of each module in the assembly could be deliberately designed, such as the gradient distribution for better transmission of local reconfiguration, and 2) the layout and the quantities of actuators could be correspondingly designed to keep the simpleness and concision. Meanwhile, self‐interference becomes worse in complicated topologies like multi‐loop assemblies. Adding self‐interference avoidance as an additional constraint (instead of post‐processing) is needed but at the cost of computational time. The analytical relationship of mechanical properties, like the stiffness and energy barrier, to the design parameters, is to be provided by assuming a simple deformation path. The influence of fabrication and the torsional stiffness of compliant joints are not thoroughly considered, and the mechanical properties are only studied in a rather unsystematic and unqualitative manner by employing a few experiments and numerical simulations with a simplified truss model. Large‐scale tessellation involving many IMSS linkages can be also explored in the future, such as deployable spatial surfaces and robotic platforms with impulsive motion, which can be actuated by only a few lightweight shape‐memory‐alloy springs. Meanwhile, it would be possible to be fabricated on a small scale, because it is easy to manufacture due to the absence of prestress.

## Conflict of Interest

The authors declare no conflict of interest.

## Author Contributions

T.Z. carried out the parametric design, numerical analysis, prototypes manufacturing, experiments, and the writing of the first draft. C.H. carried out the prototypes manufacturing of IMSS‐R6S3 linkages, and the quantitative testing of IMSS‐R4S2 with SMA actuators. Z.M. carried out the prototypes manufacturing of IMSS‐R4S2 linkages and the valuable exploration of potential fabrication methods. Y.L. provided funding acquisition, research conceptualization, supervision, and paper review and editing. All authors read and approved the final manuscript.

## Supporting information

Supporting Information

Supporting Movie S1

Supporting Movie S2

Supporting Movie S3

Supporting Movie S4

Supporting Movie S5

Supporting Movie S6

Supporting Movie S7

Supporting Information

## Data Availability

The data that support the findings of this study are available in the supplementary material of this article.
