# Supplementary material for: Intrinsically Multi‐Stable Spatial Linkages
Source: Adv Sci (Weinh). 2024 Sep 16;11(41):2402127. doi: 10.1002/advs.202402127 (PMC11538711; doi:10.1002/advs.202402127)
Supplement: Supplementary file 1 — Supporting Information [file ADVS-11-2402127-s008.pdf]

ADVANCED SCIENCE
Open Access

# Supporting Information

for *Adv. Sci.*, DOI 10.1002/advs.202402127

Intrinsically Multi-Stable Spatial Linkages

*Tong Zhou, Chong Huang, Zhuangzhi Miao and Yang Li**

Supplementary Materials of

# Intrinsically multi-stable spatial linkages

*Tong Zhou, Chong Huang, Zhuangzhi Miao, Yang Li**

---

## Contents in this PDF file:


- **Supplementary text**
  1. Details of each blocks in the inverse design framework of IMSS linkages
     - 1.1. Parameterization block
     - 1.2. Inverse design block
     - 1.3. Validation block
  2. Details of three IMSS linkage module designs
     - 2.1. Bi-stable 4R (IMSS-R4S2) linkage
     - 2.2. Quadra-stable 6R (IMSS-R6S4) linkage
     - 2.3. Tri-stable 6R (IMSS-R6S3) linkage
  3. Additional design of multi-loop IMSS linkage assemblies
     - 3.1. Three-module reconfigurable tube with tunable section area
     - 3.2. Deployable tube with designable configurations
     - 3.3. Reconfigurable robotics platform with circular coupling
  4. Validation of shape accuracy, high stiffness, and lightweight design in IMSS linkages
     - 4.1. Demonstration of the shape accuracy and high stiffness in IMSS linkages
     - 4.2. Demonstration of the lightweight design in IMSS linkages
     - 4.3. Demonstration of the reversible reconfiguration of IMSS linkages with simple actuators
  5. Stiffness analysis for IMSS-R4S2 linkage modules and alternative designs
- **Supplementary figures**
  - **S1.** Parameterization block with D-H notation
  - **S2.** Truss model for IMSS-R4S2 linkages
  - **S3.** Coupling strategies for revolute hinges
  - **S4.** Unwrapping the IMSS-R4S2 linkage
  - **S5.** Detailed physical model design for bi-materials printing
  - **S6.** Experiments preparation
  - **S7.** Pure bending machine
  - **S8.** Force-displacement curve of the IMSS-R4S2 linkage
  - **S9.** Stiffness analysis of a single TPU hinge
  - **S10.** Design of a IMSS-R6S3 linkage
  - **S11.** Demonstration of stacking coupling of IMSS linkage modules: a deployable tube


*Corresponding author: yang.li@whu.edu.cn

**S12.** Demonstration of the deployable tube with tunable spatial configurations

**S13.** Demonstration of circular coupling of IMSS linkage modules: a swimming robot

**S14.** Quantitative comparison of an IMSS linkage and a Bennett linkage

**S15.** Demonstrations of the reversible reconfiguration of bi-stable linkages

**S16.** Demonstrations of the reversible reconfiguration of the IMSS-R6S4 linkage

**S17.** Parametric analysis for IMSS-R4S2 linkages and their alternative designs.

- **Supplementary table**

**S1.** Essential properties of the materials using in the 3D printing

**S2.** Stiffness of the equivalent spring of each sample of IMSS-R4S2 linkage

- **Description of additional supplementary materials**

# 1 Details of each blocks in the inverse design framework of IMSS linkages

## 1.1 Parameterization block

The D-H notation gives an effective tool to parameterize spatial linkages[21], which is shown as Fig. S1. For a general 4R spatial linkage of four revolute hinges $H_1 - H_4$ shown in Fig. S1(A), its configuration can be expressed with one single group of D-H parameters shown in Fig. S1(B∼C). In Fig. S1(B), the direction of the axes of four revolute hinges are fixed spatially to characterize the configuration of the linkage. The connecting linkages vertical to the adjacent hinges is marked in orange, and then the new 4R spatial linkage is updated by the hinges sliding to the intersection points with the displacement $d$.

In Fig. S1(C), the direction of the updated linkages and the hinges (which is invariant) is defined. The other four D-H parameters are defined as well in Fig. S1(C), which are, 1) $l_{i,j}$, the displacement of the linkages from Hinge $H_i$ to $H_j$. 2) $r_i$, the displacement of the offset from the vertical point of $l_{i-1,i}$ to that of $l_{i,i+1}$. 3) $\alpha_{i,j}$, the angular deflection from the direction of Hinge $H_i$ to $H_j$ following the right-hand rule. 4) $\theta_i$, the rotation angle from $l_{i-1,i}$ to $l_{i,i+1}$ following the right-hand rule.

It is assumed in the multi-compatibility principle similar as in the over-constrained mechanism, that $d_i, l_{i,j}, r_i, \alpha_{i,j}$ are invariant and defined as the structural parameters while the kinematic parameter $\theta_i$ is changeable to express the distinct configurations, as shown orange and blue in Fig. S1(D). In other words, in the two structurally compatible configurations of a 4R spatial linkage, for instance, there are only one group structural parameters, yet with two different groups of kinematic parameters to characterize the two stable configurations.

## 1.2 Inverse design block

In this section, the complete design block of IMSS linkages is presented detailly from the introduction of the underlying techniques, including: 1) assessment of stiffness, 2) definition of accessibility, 3) configuration conditions, 4) detection of self-interference, and 5) coupling conditions for multiple loops of IMSS linkage, to the elaboration of the core computation units, including: 1) initial guess generator, and 2) optimization solver.

### 1.2.1 Assessment of stiffness with the truss model

The truss model[35] used in this work follows the assumption that the elastic reconfiguration of structure is a combined result of in-plane deformation, the bending, and the folding of the panels. All parts are discretized as triangular facets or revolute joints as shown in Fig. S2. The mechanical behavior is described by the equilibrium equation, the compatibility equation, and the material properties respectively as:

$$\begin{cases} \boldsymbol{A} \cdot \boldsymbol{t} = \boldsymbol{f} \\ \boldsymbol{C} \cdot \boldsymbol{d} = \boldsymbol{e} \\ \boldsymbol{G} \cdot \boldsymbol{e} = \boldsymbol{t} \end{cases} \tag{1}$$

where $\boldsymbol{A}$ is the equilibrium matrix relating the internal bar tensions $\boldsymbol{t}$ to the nodal forces $\boldsymbol{f}$, $\boldsymbol{C}$ is the compatibility matrix that translates the nodal displacement vector $\boldsymbol{d}$ to the bar extensions $\boldsymbol{e}$, and axial bar stiffness is described by the diagonal matrix $\boldsymbol{G}$. The linear-elastic properties of the 4R structure are characterized by eigenvalue decomposing its compatibility matrix and analyzing the vector space[30]. However, of the primary interest here is the stiffness matrix, which provides a relation from the external forces $\boldsymbol{f}$ to the nodal displacement $\boldsymbol{d}$, and thus:

$$\boldsymbol{K} \cdot \boldsymbol{d} = \boldsymbol{f} \rightarrow \boldsymbol{K} = \boldsymbol{C}^{\mathrm{T}} \cdot \boldsymbol{G} \cdot \boldsymbol{C} \rightarrow \boldsymbol{K}\boldsymbol{v}_i = \lambda_i \boldsymbol{v}_i \tag{2}$$

The eigenvalues $\lambda_i$ of the stiffness matrix represent the elastic energy at each reconfiguration mode $\boldsymbol{v}_i$ of the corresponding eigenmode[31]. Considering the deformation of structures, the energy is required, and thus the lowest eigenvalue is able to act as a metric to evaluate the minimum energy as deformation. The computation code of the above analysis adopted to the case in this work is found in Data S3.

### 1.2.2 Definition of target accessibility

The accessibility of the constrained optimization solver is characterized by the difference between the prescribed stable states and the reconfiguration curve, which, in other words, indicates how far the design solution deflects from the target.

$$L_{\text{init}} = \min \sum_{1}^{S} (\text{Config.} - \text{Config.}^{\text{Targ}})$$

In this work, we take the rotation angles as the expression of the target configurations $L_{\text{init}} = \min \sum_{s=1,r=R}^{S} (\theta_r^s - \theta_r^{s,\text{Targ}})$, yet it is also feasible to describe it in other forms based on the practical aims, like the arc length or the section area, for instance.

### 1.2.3 Further analysis of configuration constraints

The configuration constraints $\text{Con}^{\text{Config.}}$ refer to that the closure conditions are satisfied at the discrete states of spatial linkages. For a 4R linkage, the constraints at one of the states can be expressed with the D-H notation as below (which is derived by Wolfram language and shown step by step in Data S4).

$$\begin{aligned}
\mathbf{Con}^{\text{Config.}} &:= \mathbf{D}_{\text{Com}} = \mathbf{Q}_{\text{closure}} - \mathbf{I}_4 = \mathbf{0} \\
&:= \begin{pmatrix} \cos(\theta_4) & \sin(\theta_4) & 0 & -l_{41} \\ -\cos(\alpha_{41})\sin(\theta_4) & \cos(\alpha_{41})\cos(\theta_4) & \sin(\alpha_{41}) & -r_4\sin(\alpha_{41}) \\ \sin(\alpha_{41})\sin(\theta_4) & \sin(\alpha_{41})(-\cos(\theta_4)) & \cos(\alpha_{41}) & -r_4\cos(\alpha_{41}) \\ 0 & 0 & 0 & 1 \end{pmatrix} \cdot \\
&\quad \begin{pmatrix} \cos(\theta_3) & \sin(\theta_3) & 0 & -l_{34} \\ -\cos(\alpha_{34})\sin(\theta_3) & \cos(\alpha_{34})\cos(\theta_3) & \sin(\alpha_{34}) & -r_3\sin(\alpha_{34}) \\ \sin(\alpha_{34})\sin(\theta_3) & \sin(\alpha_{34})(-\cos(\theta_3)) & \cos(\alpha_{34}) & -r_3\cos(\alpha_{34}) \\ 0 & 0 & 0 & 1 \end{pmatrix} \cdot \\
&\quad \begin{pmatrix} \cos(\theta_2) & \sin(\theta_2) & 0 & -l_{23} \\ -\cos(\alpha_{23})\sin(\theta_2) & \cos(\alpha_{23})\cos(\theta_2) & \sin(\alpha_{23}) & -r_2\sin(\alpha_{23}) \\ \sin(\alpha_{23})\sin(\theta_2) & \sin(\alpha_{23})(-\cos(\theta_2)) & \cos(\alpha_{23}) & -r_2\cos(\alpha_{23}) \\ 0 & 0 & 0 & 1 \end{pmatrix} \cdot \\
&\quad \begin{pmatrix} \cos(\theta_1) & \sin(\theta_1) & 0 & -l_{12} \\ -\cos(\alpha_{12})\sin(\theta_1) & \cos(\alpha_{12})\cos(\theta_1) & \sin(\alpha_{12}) & -r_1\sin(\alpha_{12}) \\ \sin(\alpha_{12})\sin(\theta_1) & \sin(\alpha_{12})(-\cos(\theta_1)) & \cos(\alpha_{12}) & -r_1\cos(\alpha_{12}) \\ 0 & 0 & 0 & 1 \end{pmatrix} - \mathbf{I}_4 = 0 \\
&:= \mathbf{T}_{41}(l_{41}, \alpha_{41}, r_4, \theta_4)\mathbf{T}_{34}(l_{34}, \alpha_{34}, r_3, \theta_3)\mathbf{T}_{23}(l_{23}, \alpha_{23}, r_2, \theta_2)\mathbf{T}_{12}(l_{12}, \alpha_{12}, r_1, \theta_1) - \mathbf{I}_4 = 0
\end{aligned}$$

In this work, the configurations are prescribed with the different groups of the rotation angle $\boldsymbol{\theta}$ of linkage as in over-constrained linkages. The compatibility deviation $|\mathbf{D}_{\text{Com}}|$ is characterized to measure the incompatibility that is difference of the current state from the global minimum state (i.e., the stable states). The detail of the derivation is presented in Data S4 with Wolfram language.

### 1.2.4 Self-interference detection for theoretical models of IMSS linkage modules

The configuration conditions of spatial linkages $\text{Con}^{\text{Config.}}(\boldsymbol{l}, \boldsymbol{r}, \boldsymbol{\alpha}, \boldsymbol{\theta})$is actually not related to the displacement $\boldsymbol{d}$ of the vertical points at the revolute hinge axis. It suggests that the multi-stability is invariant whether the hinge slides along the axis as shown in Fig. 7(C), and thus the actual location changes, which is called the stability invariance principle of hinges sliding in IMSS linkages.

Though the hinges sliding has no influence to the multi-stability, the other effect on the stiffness of the current stiffness, for example, is analyzed in the main content. But the primary concern lays on the bypassing strategy of the self-interference described above. The first thing is to detect the self-interference. G-J-K

algorithm[32,33] is used here for the tetrahedron-shaped IMSS linkages (which happens to be a convex shape) in this work. The core is included in Data S5.

The detection stops as any two of the tetrahedrons have no self-interference at the current configuration. Ideally, the detection would then proceed to the next configuration, and if there is no interference at all states along the deformation, the detection would be done with the no-self-interference conclusion. However, considering the computing efficiency, the detection is put in the validation block instead of the optimization solver acting as a strict non-linear constraint. Empirically for IMSS-R4 linkages, it is found that there is no self-interference along the overall reconfiguration, if it is free from the interference just at the stable states.

#### 1.2.5 Coupling strategy of IMSS linkages with multiple loops

When assembling IMSS linkage modules into a multi-loop IMSS linkage assembly, the principle is not to introduce any additional degree of freedom such that the multi-loop assemblage is still a multi-stable structure as the name suggests. Previous works propose an effective coupling strategy for multi-loop mechanisms to form the deployable arc or the reconfigurable surface. The basic coupling idea is shown in Fig. S3 that two revolute hinges have to be aligned to the identical hinge axis direction, while the respective leaves are fixed with two pairs of coupling frames. The reconfiguration of the starting module is transmitted through the coupled hinge, and its remaining single degree of freedom makes the reconfiguration continue to the next.

In this work, we follow this strategy to construct multi-loop IMSS linkages. Obviously, there are two different types of chirality for stacking coupling and chaining coupling, which are regulated mathematically with different coupling conditions:

$$\Delta\theta_i = (-1)^C \Delta\theta_{i+1}$$

where $\Delta\theta_i$ and $\Delta\theta_{i+1}$ refer to the variation of the rotation angle of the coupled hinges $\mathrm{H}_i$ and $\mathrm{H}_{i+1}$ in the adjacent modules and $C$ characterizes the chirality of the coupling strategy adopted.

#### 1.2.6 Computation unit: initial guess generator

It is greatly known that "Fmincon" solver in MATLAB is sensitive to the initial guess fed into[34], especially for the case with lots of non-linear constraints like in this work. This generator is appointed to feed the right initial guess to the main optimization solver.

Due to the underlying connection of the multi-stable structure and the corresponding over-constrained mechanism, it comes naturally that the over-constrained mechanism is suitable for the initial guess. There are in fact several types of over-constrained mechanism for linkages. In this work, Bennett linkage and general line-symmetric Bricard linkage are adopted for 4R and 6R linkages respectively. After determining its formation, on the other hand, the configuration constraints for over-constrained mechanisms are imposed then. In other words, the initial guess generator is also a minor constrained optimization solver with the non-linear constraints of configuration, expressed as:

$$\mathrm{Con}^{\mathrm{Config.}}_{\mathrm{Bennett}} = \begin{cases} l_{12} = l_{34} = l_1 \\ l_{23} = l_{41} = l_2 \\ \alpha_{12} = \alpha_{34} = \alpha_1 \\ \alpha_{23} = \alpha_{41} = \alpha_2 \\ \frac{\sin\alpha_1}{l_1} = \frac{\sin\alpha_2}{l_2} \\ r_1 = r_2 = r_3 = r_4 = 0 \end{cases}, \mathrm{Con}^{\mathrm{Config.}}_{\mathrm{Bricard}} = \begin{cases} l_{12} = l_{45}, l_{23} = l_{56}, l_{34} = l_{61}, \\ \alpha_{12} = \alpha_{45}, \alpha_{23} = \alpha_{56}, \alpha_{34} = \alpha_{61}, \\ r_1 = r_4, r_2 = r_5, r_3 = r_6 \end{cases}$$

As for the objective function of the minor solver, it is selected to minimize the distance between the target configurations and the kinematic curve of the output over-constrained mechanism. The whole point of the initial guess generator is to get an over-constrained mechanism with the same formation of 4R or 6R, and being closed to the target configurations as much as possible. The non-linear constrained optimization problem is solved by "Fmincon" function in MATLAB. It is not necessarily feasible if the initial guess in this

minor solver is randomly picked, and thus the feedback loop needs to be involved in the inverse design framework.

#### 1.2.7 Computation unit: optimization solver

After the optimal initial guess is output from the last unit, it is then put into the optimization solver to generate the IMSS linkage, which includes the objective function to maximum the overall stiffness at the target configurations, and the constraints of target accessibility, configuration conditions, and no-self-interference conditions. All of these are described in the previous content. It is still feasible to add some new constraints based on the practical needs, such as the regulation of size, or the coupling conditions for multi-loop IMSS linkages, as long as not too strict.

It should be noted that the no-self-interference conditions are optional in this unit, out of the consideration of computing efficiency. It could be conducted afterwards in the validation block.

### 1.3 Validation block

There are three units in this block: 1) Theorical analysis unit consists of stiffness assessment to avoid pseudo-mechanisms and deformation simulation for self-interference detection; 2) Fabrication unit aims to bring the theoretical model to the reality. 3) Experiments unit then assesses the design and the pre-set target.

#### 1.3.1 Deformation analysis for IMSS linkages

In this work, we adopt the nudged elastic band (NEB) method[23,24] to find the deformation path with the minimum energy from one configuration to another. Visually, our aim is to find a series of continuous states $\mathbf{Y} = \{\mathrm{Y}_1, \mathrm{Y}_2, ..., \mathrm{Y}_k\}$ between the two stable states $\{\mathrm{Y}_0, \mathrm{Y}_n\}$, where any configuration point at the formed reconfiguration path is a local minimum of the overall energy landscape.

There are in fact many algorithms[35,36] available for accomplishing the simulation, yet at the cost of computing complexity or specific appointment of actuation. NEB method otherwise provides a direct and feasible algorithm for the path finding by introducing springs with stiffness between the adjacent configurations $\{\mathrm{Y}_i, \mathrm{Y}_{i+1}\}$ and minimize the overall energy, which is expressed as:

$$W_{all}(\mathbf{Y}) = W(\mathbf{Y}) + \frac{1}{2}\Sigma_{i=1}^{n}(e_i|\mathrm{Y}_i - \mathrm{Y}_{i-1}|^2)$$

where $W(\mathbf{Y})$ denotes the total elastic energy as the reconfiguration, $e_i$ is the elastic constant for the imaginary springs between the configuration points, and the elongation of the spring is characterized by the L2-norm of the adjacent configurations.

The direction to minimize the energy can be selected respectively based on the current configurations, where the elastic energy would be projected perpendicularly and the energy of springs would be projected tangentially. The current configurations chain $\mathbf{Y}^k$ would follow this direction NEB and relocated by the forces (i.e., the projection of energy) to find the optimal configurations chain $\mathbf{Y}^{opt}$, which indicated the minimum-energy path.

#### 1.3.2 Fabrication routes for IMSS linkages

The formation of tetrahedral is used in this work to link the spatial revolute hinges with the designed relative location, out of the consideration of intuitiveness and simpleness. It provides two ways to fabricate, by either paper models, which is fast but fragile, or by 3D printing, which is deliberate but durable.

The balanced property of paper between the tensile strength to carry in-plane deformation, and the flexibility of smooth rotation makes it perfect for revolute hinges. So, the left challenge is to unwrap the tetrahedral links into a plane with preserving the relative location of the spatial hinges. There are lots of classic UV unwrapping algorithms for this problem[37], but it is easy to unwrap intuitively in this case due to the tetrahedral shapes (codes provided in Data S6) as in Fig. S4(A). Fig. S4(B) provides a plane pattern of bistable

4R linkage designs. Other patterns with the various angular targets are provided in Data S7. Following the instructions in the figure, the paper models would be folded physically and then exhibits the slight bi-stability as designed. We recommend to fold these by one's self to get a direct understanding.

Paper models provide a fast tool for the validation to the no-self-interference and target accessibility, yet the great issue is that it is fragile and lack of accuracy, which comes from the negative consequence of accumulated folding error. One option is to use bi-material 3D printing to fabricate the all-in-one physical model[38], where the material with lower Young's modulus is chosen to form hinges, while the higher and tough one is to form links, as shown in Fig. S5(A). It is free from the error of folding or handcrafting and thus reaches to a higher accuracy. To preserve the shape rotation of hinges, the gap of soft material ought to be as narrow as possible, yet it should also penetrate into another material to be integrated. One of the challenges is to interlock the distinct two materials. In this work, we have two techniques to ensure the effectiveness of interlocking. The first is to use carbon fibers (PolyMide™ PA12-CF) and TPU (PolyFlex™TPU95) as two materials, which prefer sticking together as we observed. The essential property of them is provided in Table S1. The second is to use random micro bolt-shape structures at the interface to lock the parts with different materials as shown in Fig. S5(B).

In this work, an upgraded so-called segmented fabrication method is proposed to firstly deal with each hinge individually with bi-material printing, and then to connect each corresponding two hinges to form a complete spatial IMSS linkage. It is good for fabricate a more high-quality hinges than direct fabricating to the whole linkage.

### 1.3.3 Experiments units

There are three main parts of function tests, and the design would not be complete until all the extra tests based on the practical finished.

The basic function test of multi-stability can be validated by the intuitive check of paper models or 3D-printing models (which is recommended). The self-interference test can be conducted by paper models before 3D printing out of the consideration of time saving.

The actuation test is a core function test for the validation of its stiffness. In this work, two approaches are developed to test it. The set-up of the actuation test through cable is shown in Fig. S6. When the actuation follows at the direction of the rail track, the component movement of the actuation imposed on the 4R IMSS linkage module can be calculated by the geometric relation in the figure. Another actuation test is conducted through a pure bending machine, which was developed in this literature[39], and the schematic of the layout is referred and shown in Fig. S7. It is noted that other performance tests like the experiments of deploying or clamping can be also completed. It is not mentioned here for simplicity.

## 2 Details of three IMSS linkage module designs

### 2.1 Bi-stable 4R (IMSS-R4S2) linkages

### 2.1.1 Presentation of all designable parameters

The IMSS-R4S2 linkage in the main text is expressed by the D-H notation as:

$$\begin{array}{llll} l_{12} = 1.70460587694834 & l_{23} = 1.33814287369465 & l_{34} = 3.02172916271496 & l_{41} = 2.96823559654025 \\ \alpha_{12} = 66.58793357715^\circ & \alpha_{23} = 141.334495994^\circ & \alpha_{34} = 66.08475699290^\circ & \alpha_{41} = 135.582329572^\circ \\ r_1 = 1.31329337369676 & r_2 = 0.409349062959366 & r_3 = 2.30246453117182 & r_4 = 0.00124368465101332 \\ d_1 = -0.4 & d_2 = 0.0 & d_3 = 1.0 & d_4 = -1.0 \end{array}$$

and the kinematic parameters of rotation angle of hinges at two stable states:

$\theta_1^{\mathrm{S1}} = 19.999999971680648°$ $\theta_2^{\mathrm{S1}} = 207.4244243182846°$ $\theta_3^{\mathrm{S1}} = 358.0600977225914°$
$\theta_4^{\mathrm{S1}} = 220.2648323466420e°$
$\theta_1^{\mathrm{S2}} = 110.7077863459363°$ $\theta_2^{\mathrm{S2}} = 95.599566062606780°$ $\theta_3^{\mathrm{S2}} = 242.9392121692375°$
$\theta_4^{\mathrm{S2}} = 266.8420377898646°$

The objectives of the inverse design solver are prescribed as: $\theta_1^{\mathrm{S1}} = 20°$ and $\theta_2^{\mathrm{S1}} = 110°$. After the sliding of the hinges, which is the parameters **d**, the self-interference is avoided, and the end points of the spatial linkages can be calculated by the function provided in Data S1. The comparison between the theoretical model and the physical model (which are included in Data S9) can be conducted in the CAD software. The complete design results can be found in Data S9 in the supplementary data file, where the information of the end points is stored in the variable named "*MATXPointsS*".

### 2.1.2 Theoretical validation of the constraints

The compatibility deviation $|\mathbf{D}_{\mathrm{com}}|$ at the two stable states can be calculated by the above designed parameters, and examined if it is equal to zero respectively:

$$
\begin{aligned}
\mathbf{D}_{\mathrm{com}}^{\mathrm{S1}} &= \mathbf{T}_{41}\mathbf{T}_{34}\mathbf{T}_{23}\mathbf{T}_{12} - \mathbf{I}_4 \\
&= \begin{pmatrix} -0.763065 & -0.646322 & 0 & -2.96824 \\ -0.46164 & 0.545025 & 0.699884 & -0.000870435 \\ -0.45235 & 0.534057 & -0.714257 & 0.00088831 \\ 0 & 0 & 0 & 1 \end{pmatrix} \cdot \\
&\begin{pmatrix} 0.999427 & -0.0338512 & 0 & -3.02173 \\ 0.0137228 & 0.405152 & 0.914146 & -2.10479 \\ -0.030945 & -0.913622 & 0.405385 & -0.933384 \\ 0 & 0 & 0 & 1 \end{pmatrix} \cdot \\
&\begin{pmatrix} -0.887619 & -0.460578 & 0 & -1.33814 \\ -0.359623 & 0.693059 & 0.624773 & -0.25575 \\ -0.287757 & 0.55456 & -0.780807 & 0.319622 \\ 0 & 0 & 0 & 1 \end{pmatrix} \cdot \\
&\begin{pmatrix} 0.939693 & 0.34202 & 0 & -1.70461 \\ -0.135899 & 0.373379 & 0.917671 & -1.20517 \\ 0.313862 & -0.862329 & 0.397341 & -0.521826 \\ 0 & 0 & 0 & 1 \end{pmatrix} - \mathbf{I}_4 = \mathbf{0}
\end{aligned}
$$

, and

$$
\begin{aligned}
\mathbf{D}_{\mathrm{com}}^{\mathrm{S2}} &= \mathbf{T}_{41}\mathbf{T}_{34}\mathbf{T}_{23}\mathbf{T}_{12} - \mathbf{I}_4 \\
&= \begin{pmatrix} -0.0550889 & -0.998481 & 0 & -2.96824 \\ -0.713172 & 0.0393477 & 0.699884 & -0.000870435 \\ -0.698821 & 0.0385558 & -0.714257 & 0.00088831 \\ 0 & 0 & 0 & 1 \end{pmatrix} \cdot \\
&\begin{pmatrix} -0.454936 & -0.890524 & 0 & -3.02173 \\ 0.361005 & -0.184424 & 0.914146 & -2.10479 \\ -0.814069 & 0.415878 & 0.405385 & -0.933384 \\ 0 & 0 & 0 & 1 \end{pmatrix} \cdot \\
&\begin{pmatrix} -0.0975754 & 0.995228 & 0 & -1.33814 \\ 0.777081 & 0.0761875 & 0.624773 & -0.25575 \\ 0.621791 & 0.0609624 & -0.780807 & 0.319622 \\ 0 & 0 & 0 & 1 \end{pmatrix} \cdot \\
&\begin{pmatrix} -0.353602 & 0.935396 & 0 & -1.70461 \\ -0.371671 & -0.140501 & 0.917671 & -1.20517 \\ 0.858386 & 0.32449 & 0.397341 & -0.521826 \\ 0 & 0 & 0 & 1 \end{pmatrix} - \mathbf{I}_4 = \mathbf{0}
\end{aligned}
$$

The stiffness at two stable states is calculated as: $\kappa^{\mathrm{S1}} = 0.0267$, $\kappa^{\mathrm{S2}} = 0.0062$, which suggests that it would be more hard to reconfigure from State state $S_1$ to other states, yet it might be effected by the fabrication error.

### 2.1.3 Quantitative analysis of physical models

In this section, the stiffness error of the physical model are investigated and quantitatively compared to the theoretical model.

The stiffness is characterized by measuring the maximum traction force $F^{\mathrm{S_1S_2}}, F^{\mathrm{S_2S_1}}$ of two processes from one stable state to another state. The experiments for a IMSS-R4S2 linkage is conducted with the actuating equipment shown in Fig. S6 and Fig. S7. The force-displacement curve of two process is compared in Fig. S8, where the maximum actuating force of the process from Stable state $S_1$ to Stable state $S_2$, $F^{\mathrm{S_1}}_{\mathrm{max}} = 2.51$ N, and the maximum traction force of the process from Stable state $S_2$ to Stable state $S_1$, $F^{\mathrm{S_2}}_{\mathrm{max}} = 1.63$ N. The difference between two forces $F^{\mathrm{S_1}}/F^{\mathrm{S_2}} = 1.540$ is not exactly as much as indicated by the theoretical values $\kappa^{\mathrm{S1}}/\kappa^{\mathrm{S2}} = 4.28$, which is mostly effected by the fabrication error and imperfection, such as the different property of hinges in the ideal model and in the physical model.

Further investigation of the relation between the property of hinges and the stiffness of the physical model is conducted by comparing five IMSS-R4S2 physical samples with different sizes of hinges. These samples are derived from one IMSS linkage design, thus with the same theoretical stiffness. However, there is an obvious divergence of stiffness among these physical samples as indicated in Fig. 2(I). That is because of the different property of each hinge, and thus the additional potential engy introduced by the hinges is different.

As show in Fig. S9(A), an ideal TPU-made hinge can be parameterized by three distance: the axis distance $a_h$, the width $b_h$, and the thickness $t_h$. The axis distance is prescribed by the configuration design of the model and unified into a set distance of 5 mm, while the specific value of width and thickness would determine the property of the hinge. In the non zero-energy reconfiguration of the IMSS linkage, the energy barrier is constructed by the deformation of each hinge in the linkage. There are two parts of deformation, which are the bending and the stretching. Therefore, the hinge is equivalent to two springs, a torsional spring and a translational spring, respectively. The stiffness of these two springs are calculated as:

$$\begin{cases} \kappa_{tr} = & \frac{E \cdot a_h \cdot (t_h)^3}{12 b_h} \\ \kappa_{ts} = & \frac{E \cdot a_h \cdot t_h}{b_h} \end{cases}$$

where $E$ is the Young's modulus of TPU materials and equal to 9.4 MPa according to Table S1. Thus, the stiffness of two equivalent springs in the hinge of each sample can be calculated as listed in Table S2. For the equivalent translational spring, a higher stiffness $\kappa'_{tr} > \kappa_{tr}$ would increase the energy barrier $\Delta E$ between two stable states, because there is more resistance force as the non-zero-energy reconfiguration from one stable state to another one, as indicated in Fig. S9(B). However, it would not significantly change the potential energy $E_s$ at each stable state, because there is no deformation at each stable state. Mathematically, it is expressed as:

$$\begin{cases} \Delta E(\kappa_{tr}) < \Delta E(\kappa'_{tr}) \\ E_s(\kappa_{tr}) = E_s(\kappa'_{tr}) = 0, \quad \forall s \in \{1, 2, ...\} \end{cases}$$

For the torsional spring equivalent, the potential energy $E_s, s \in \{1, 2, ...\}$ at local stable states is affected by its spring stiffness. Specifically, when the spring stiffness increases $\kappa_{\mathrm{to}} \to \kappa'_{\mathrm{to}}$, the potential energy $E_a$ at each stable state would all increase. This would decrease the energy barrier $\Delta E(\kappa_{\mathrm{to}})$ as indicated in Fig. S9(C). Mathematically, it is expressed as:

$$\begin{cases} \Delta E(\kappa_{to}) > \Delta E(\kappa'_{to}) \\ E_s(\kappa_{to}) < E_s(\kappa'_{to}), \quad s \in \{1, 2, ...\} \end{cases}$$

The result of the actuating experiment is shown in Fig. S9(D), where Sample 1 has the lowest actuating force, which suggests a lowest energy barrier, and Sample 3 has the highest due to the highest $\kappa_{tr}$ among all

samples. The video of testing is included in Movie S2 of Supplementary materials. There is no bi-stability observed in Sample 5 as indicated in Movie S2 of Supplementary materials due to its high $\kappa_{to}$, even its $\kappa_{tr}$ is equal to that of Sample 3.

### 2.2 Quadra-stable 6R (IMSS-R6S4) linkages

#### 2.2.1 Derivation of D-H parameters from two IMSS-R4S2 linkage modules

The coupling of the two modules ($L_1$ and $L_2$) into a 6R linkage is depicted in Fig. 3(C). According to the coupling condition, $l_{41}^{\mathrm{L1}} = l_{41}^{\mathrm{L1}}, \alpha_{41}^{\mathrm{L1}} = \alpha_{41}^{\mathrm{L1}}$, the designable parameters of the 6R linkage are derived from two IMSS-R4S2 linkage modules and thus expressed as:

$$\begin{array}{llllll}
l_{12} = l_{12}^{\mathrm{L1}} & l_{23} = l_{23}^{\mathrm{L1}} & l_{34} = l_{34}^{\mathrm{L1}} & l_{45} = l_{34}^{\mathrm{L1}} & l_{56} = l_{23}^{\mathrm{L1}} & l_{61} = l_{12}^{\mathrm{L1}} \\
\alpha_{12} = \alpha_{12}^{\mathrm{L1}} & \alpha_{23} = \alpha_{23}^{\mathrm{L1}} & \alpha_{34} = \alpha_{34}^{\mathrm{L1}} & \alpha_{45} = \alpha_{34}^{\mathrm{L1}} & \alpha_{56} = \alpha_{23}^{\mathrm{L1}} & \alpha_{61} = \alpha_{12}^{\mathrm{L1}} \\
r_1 = r_1^{\mathrm{L1}} - r_1^{\mathrm{L1}} & r_2 = r_2^{\mathrm{L1}} & r_3 = r_3^{\mathrm{L1}} & r_4 = r_4^{\mathrm{L1}} - r_4^{\mathrm{L1}} & r_5 = -r_3^{\mathrm{L1}} & r_6 = -r_2^{\mathrm{L1}} \\
\theta_1 = \theta_1^{\mathrm{L1}} - \theta_1^{\mathrm{L2}} - 180^\circ & \theta_2 = \theta_2^{\mathrm{L1}} & \theta_3 = \theta_3^{\mathrm{L1}} & \theta_4 = \theta_4^{\mathrm{L1}} - \theta_4^{\mathrm{L2}} + 180^\circ & \theta_5 = -\theta_3^{\mathrm{L2}} & \theta_6 = -\theta_2^{\mathrm{L2}}
\end{array}$$

#### 2.2.2 Presentation of all designable parameters and theoretical validation

While the D-H parameters of the two IMSS-R4S2 linkage module are listed as:

$$\begin{array}{llll}
l_{12}^{\mathrm{L1}} = 1.7046 & l_{23}^{\mathrm{L1}} = 1.3381 & l_{34}^{\mathrm{L1}} = 3.0217 & l_{41}^{\mathrm{L1}} = 2.9682 \\
\alpha_{12}^{\mathrm{L1}} = 66.595^\circ & \alpha_{23}^{\mathrm{L1}} = 141.33^\circ & \alpha_{34}^{\mathrm{L1}} = 66.08^\circ & \alpha_{41}^{\mathrm{L1}} = 135.58^\circ \\
r_1^{\mathrm{L1}} = 1.3133 & r_2^{\mathrm{L1}} = 0.4093 & r_3^{\mathrm{L1}} = 2.3025 & r_4^{\mathrm{L1}} = 0.0012 \\
\theta_1^{\mathrm{L1,S1}} = 20.00^\circ & \theta_2^{\mathrm{L1,S1}} = 207.42^\circ & \theta_3^{\mathrm{L1,S1}} = 358.06^\circ & \theta_4^{\mathrm{L1,S1}} = 220.26^\circ \\
\theta_1^{\mathrm{L1,S2}} = 110.71^\circ & \theta_2^{\mathrm{L1,S2}} = 95.60^\circ & \theta_3^{\mathrm{L1,S2}} = 242.94^\circ & \theta_4^{\mathrm{L1,S2}} = 266.84^\circ
\end{array}$$

and

$$\begin{array}{llll}
l_{12}^{\mathrm{L2}} = 3.0927 & l_{23}^{\mathrm{L2}} = 2.6431 & l_{34}^{\mathrm{L2}} = 3.2219 & l_{41}^{\mathrm{L2}} = 2.9682 \\
\alpha_{12}^{\mathrm{L2}} = 134.05^\circ & \alpha_{23}^{\mathrm{L2}} = 141.41^\circ & \alpha_{34}^{\mathrm{L2}} = 129.85^\circ & \alpha_{41}^{\mathrm{L2}} = 135.58^\circ \\
r_1^{\mathrm{L2}} = 0.1209 & r_2^{\mathrm{L2}} = 0.7131 & r_3^{\mathrm{L2}} = 0.1280 & r_4^{\mathrm{L2}} = 0.7061 \\
\theta_1^{\mathrm{L2,S1}} = -20.01^\circ & \theta_2^{\mathrm{L2,S1}} = -189.83^\circ & \theta_3^{\mathrm{L2,S1}} = 5.25^\circ & \theta_4^{\mathrm{L2,S1}} = 169.03^\circ \\
\theta_1^{\mathrm{L2,S2}} = 200.71^\circ & \theta_2^{\mathrm{L2,S2}} = -33.34^\circ & \theta_3^{\mathrm{L2,S2}} = -166.84^\circ & \theta_4^{\mathrm{L2,S2}} = 57.50^\circ
\end{array}$$

The D-H parameters of the coupled IMSS-R6S4 linkage are therefore calculated as follows:

$$\begin{array}{llllll}
l_{12} = 1.7046 & l_{23} = 1.3381 & l_{34} = 3.0217 & l_{45} = 3.2219 & l_{56} = 2.6431 & l_{61} = 3.09272 \\
\alpha_{12} = 66.59^\circ & \alpha_{23} = 141.33^\circ & \alpha_{34} = 66.08^\circ & \alpha_{45} = 129.85^\circ & \alpha_{56} = 141.41^\circ & \alpha_{61} = 134.05^\circ \\
r_1 = 1.1923 & r_2 = 0.40936 & r_3 = 2.3025 & r_4 = -0.7049 & r_5 = -0.1280 & r_6 = -0.7131
\end{array}$$

and the kinematic parameters at four stable states:

$$\begin{array}{llllll}
\theta_1^{\mathrm{S1}} = -139.99^\circ & \theta_2^{\mathrm{S1}} = 207.42^\circ & \theta_3^{\mathrm{S1}} = 358.06^\circ & \theta_4^{\mathrm{S1}} = 231.23^\circ & \theta_5^{\mathrm{S1}} = -5.25^\circ & \theta_6^{\mathrm{S1}} = 189.83^\circ \\
\theta_1^{\mathrm{S2}} = -49.28^\circ & \theta_2^{\mathrm{S2}} = 95.60^\circ & \theta_3^{\mathrm{S2}} = 242.94^\circ & \theta_4^{\mathrm{S2}} = 277.81^\circ & \theta_5^{\mathrm{S2}} = -5.25^\circ & \theta_6^{\mathrm{S2}} = 189.83^\circ \\
\theta_1^{\mathrm{S3}} = -270.00^\circ & \theta_2^{\mathrm{S3}} = 95.60^\circ & \theta_3^{\mathrm{S3}} = 242.94^\circ & \theta_4^{\mathrm{S3}} = 29.34^\circ & \theta_5^{\mathrm{S3}} = 166.84^\circ & \theta_6^{\mathrm{S3}} = 33.35^\circ \\
\theta_1^{\mathrm{S4}} = -0.71^\circ & \theta_2^{\mathrm{S4}} = 207.42^\circ & \theta_3^{\mathrm{S4}} = 358.06^\circ & \theta_4^{\mathrm{S4}} = 342.76^\circ & \theta_5^{\mathrm{S4}} = 166.84^\circ & \theta_6^{\mathrm{S4}} = 33.35^\circ
\end{array}$$

The compatibility deviation $|\mathbf{D}_{\mathrm{com}}|$ at the four stable states could be calculated by the above designed parameters, and examined if it is equal to zero respectively, which is not presented here for simplicity. The comparison between the theoretical model and the physical model (which are included in Data S9) can be conducted in the CAD software. The code of calculation could be found in Data S1 of Supplementary materials.

### 2.3 Tri-stable 6R (IMSS-R6S3) linkages

Besides the method based on multi-loop coupling, the inverse design method could also be capable of generating multi-stable 6R linkages with prescriptable stable states. As more designable parameters (six digits for each type of parameters) are needed to parameterize the six hinges in a 6R linkage, it results a IMSS-R6S3 linkages with three prescribed stable states are generated, while the maximum quantity of stable states of IMSS-R4 linkages is empirically two. The multi-stability is assigned as $\hat{\theta}_1^{\text{S1}} = -30°, \hat{\theta}_1^{\text{S2}} = 60°, \hat{\theta}_1^{\text{S3}} = 120°$ for the tri-stable 6R linkage design in Fig. S10(A). The D-H parameters of the IMSS-R6S3 linkage are listed as:

| | | | | | |
|---|---|---|---|---|---|
| $l_{12} = 1.7084$ | $l_{23} = 3.0496$ | $l_{34} = 0.9401$ | $l_{45} = 1.4568$ | $l_{56} = 2.8710$ | $l_{61} = 4.2119$ |
| $\alpha_{12} = 103.37°$ | $\alpha_{23} = 114.32°$ | $\alpha_{34} = -55.41°$ | $\alpha_{45} = 116.65°$ | $\alpha_{56} = 121.10°$ | $\alpha_{61} = 54.29°$ |
| $r_1 = 1.0158$ | $r_2 = -3.3884$ | $r_3 = -4.0746$ | $r_4 = 4.4358$ | $r_5 = 5.9605$ | $r_6 = -0.3926$ |
| $\theta_1^{\text{S1}} = -30.00°$ | $\theta_2^{\text{S1}} = 116.61°$ | $\theta_3^{\text{S1}} = -51.43°$ | $\theta_4^{\text{S1}} = -156.59°$ | $\theta_5^{\text{S1}} = -74.44°$ | $\theta_6^{\text{S1}} = -65.00°$ |
| $\theta_1^{\text{S2}} = 68.93°$ | $\theta_2^{\text{S2}} = 89.01°$ | $\theta_3^{\text{S2}} = -148.41°$ | $\theta_4^{\text{S2}} = 69.92°$ | $\theta_5^{\text{S2}} = 226.52°$ | $\theta_6^{\text{S2}} = -13.44°$ |
| $\theta_1^{\text{S3}} = 119.92°$ | $\theta_2^{\text{S3}} = 119.59°$ | $\theta_3^{\text{S3}} = 13.00°$ | $\theta_4^{\text{S3}} = 303.56°$ | $\theta_5^{\text{S3}} = 148.22°$ | $\theta_6^{\text{S3}} = -91.95°$ |

The examination of closure conditions is conducted as follow:

$$
\begin{aligned}
\mathbf{D}_{\text{com}}^{\text{S1}} &= \mathbf{T}_{61}\mathbf{T}_{56}\mathbf{T}_{45}\mathbf{T}_{34}\mathbf{T}_{23}\mathbf{T}_{12} - \mathbf{I}_4 \\
&= \begin{pmatrix} 0.866 & -0.116 & -0.487 & 1.480 \\ -0.500 & -0.200 & -0.843 & -0.854 \\ 0 & 0.973 & -0.231 & 1.016 \\ 0 & 0 & 0 & 1 \end{pmatrix} \cdot \\
&\begin{pmatrix} -0.448 & 0.368 & 0.815 & -1.366 \\ 0.894 & 0.184 & 0.408 & 2.727 \\ 0 & 0.911 & -0.412 & -3.388 \\ 0 & 0 & 0 & 1 \end{pmatrix} \cdot \\
&\begin{pmatrix} 0.624 & 0.444 & 0.644 & 0.586 \\ -0.782 & 0.354 & 0.513 & -0.735 \\ 0 & -0.823 & 0.568 & -4.075 \\ 0 & 0 & 0 & 1 \end{pmatrix} \cdot \\
&\begin{pmatrix} -0.918 & -0.178 & -0.355 & -1.337 \\ -0.397 & 0.412 & 0.820 & -0.579 \\ 0 & 0.894 & -0.449 & 4.436 \\ 0 & 0 & 0 & 1 \end{pmatrix} \cdot \\
&\begin{pmatrix} 0.268 & -0.498 & -0.825 & 0.770 \\ -0.963 & -0.139 & -0.230 & -2.766 \\ 0 & 0.856 & -0.517 & 5.961 \\ 0 & 0 & 0 & 1 \end{pmatrix} \cdot \\
&\begin{pmatrix} 0.423 & 0.529 & -0.736 & 1.780 \\ -0.906 & 0.247 & -0.343 & -3.817 \\ 0 & 0.812 & 0.584 & -0.393 \\ 0 & 0 & 0 & 1 \end{pmatrix} - \mathbf{I}_4 = \mathbf{0}
\end{aligned}
$$

,

$$
\begin{aligned}
\mathbf{D}_{\text{com}}^{\text{S2}} &= \mathbf{T}_{61}\mathbf{T}_{56}\mathbf{T}_{45}\mathbf{T}_{34}\mathbf{T}_{23}\mathbf{T}_{12} - \mathbf{I}_4 \\
&= \begin{pmatrix} 0.360 & 0.216 & 0.908 & 0.614 \\ -0.933 & -0.083 & -0.350 & 1.594 \\ 0 & 0.973 & -0.231 & 1.016 \\ 0 & 0 & 0 & 1 \end{pmatrix} \cdot \\
&\begin{pmatrix} 0.017 & 0.412 & 0.911 & 0.052 \\ 1.000 & -0.007 & -0.016 & 3.049 \\ 0 & 0.911 & -0.412 & -3.388 \\ 0 & 0 & 0 & 1 \end{pmatrix} \cdot \\
&\begin{pmatrix} -0.852 & 0.297 & 0.431 & -0.801 \\ -0.524 & -0.484 & -0.701 & -0.492 \\ 0 & -0.823 & 0.568 & -4.075 \\ 0 & 0 & 0 & 1 \end{pmatrix} \cdot \\
&\begin{pmatrix} 0.343 & 0.421 & 0.839 & 0.500 \\ 0.939 & -0.154 & -0.307 & 1.368 \\ 0 & 0.894 & -0.449 & 4.436 \\ 0 & 0 & 0 & 1 \end{pmatrix} \cdot \\
&\begin{pmatrix} -0.688 & -0.375 & -0.621 & -1.975 \\ -0.726 & 0.355 & 0.589 & -2.083 \\ 0 & 0.856 & -0.517 & 5.961 \\ 0 & 0 & 0 & 1 \end{pmatrix} \cdot \\
&\begin{pmatrix} 0.973 & 0.136 & -0.189 & 4.097 \\ -0.232 & 0.568 & -0.790 & -0.979 \\ 0 & 0.812 & 0.584 & -0.393 \\ 0 & 0 & 0 & 1 \end{pmatrix} - \mathbf{I}_4 = \mathbf{0}
\end{aligned}
$$

,

and

$$
\begin{aligned}
\mathbf{D}_{\text{com}}^{\text{S3}} &= \mathbf{T}_{61}\mathbf{T}_{56}\mathbf{T}_{45}\mathbf{T}_{34}\mathbf{T}_{23}\mathbf{T}_{12} - \mathbf{I}_4 \\
&= \begin{pmatrix} -0.499 & 0.200 & 0.843 & -0.852 \\ 0.867 & 0.115 & 0.485 & 1.481 \\ 0 & 0.973 & -0.231 & 1.016 \\ 0 & 0 & 0 & 1 \end{pmatrix} \cdot \\
&\begin{pmatrix} -0.494 & 0.358 & 0.792 & -1.506 \\ 0.870 & 0.203 & 0.450 & 2.652 \\ 0 & 0.911 & -0.412 & -3.388 \\ 0 & 0 & 0 & 1 \end{pmatrix} \cdot \\
&\begin{pmatrix} 0.974 & -0.128 & -0.185 & 0.916 \\ 0.225 & 0.553 & 0.802 & 0.212 \\ 0 & -0.823 & 0.568 & -4.075 \\ 0 & 0 & 0 & 1 \end{pmatrix} \cdot \\
&\begin{pmatrix} 0.553 & -0.374 & -0.745 & 0.805 \\ -0.833 & -0.248 & -0.494 & -1.214 \\ 0 & 0.894 & -0.449 & 4.436 \\ 0 & 0 & 0 & 1 \end{pmatrix} \cdot \\
&\begin{pmatrix} -0.850 & 0.272 & 0.451 & -2.441 \\ 0.527 & 0.439 & 0.728 & 1.512 \\ 0 & 0.856 & -0.517 & 5.961 \\ 0 & 0 & 0 & 1 \end{pmatrix} \cdot \\
&\begin{pmatrix} 0.140 & 0.578 & -0.804 & 0.590 \\ -0.990 & 0.082 & -0.114 & -4.170 \\ 0 & 0.812 & 0.584 & -0.393 \\ 0 & 0 & 0 & 1 \end{pmatrix} - \mathbf{I}_4 = \mathbf{0}
\end{aligned}
$$

A higher design error is observed in the tri-stable 6R linkage,

$$\delta_{\theta \mathrm{h}}(\theta_{\theta \mathrm{h}}^{\mathrm{S1}}, ..., \theta_{\theta \mathrm{h}}^{\mathrm{Sn}}) = 1/n \cdot \sum |\theta_h^s - \hat{\theta}_h^s| = \delta_{\theta 1}^{6\mathrm{R}}(-30°, 68.93°, 119.92°) = 3.001°$$

compared to that in the IMSS-R4S2 linkage, due to the dispersion of incompatibility on more configurations. The comparison between the theoretical model and the physical model (which are included in Data S9) can be conducted in the CAD software. The reconfiguration among the stable states is shown in Fig. S10(B). The whole process is shown in Movie S6 of Supplementary Materials.

## 3 Additional design of multi-loop IMSS linkage assemblies

In this section, we are presenting two additional preliminary designs of IMSS linkage assembly with more details, including a reconfiguration tube with tunable section area, and a reconfigurable robotics platform with circular coupling.

### 3.1 Three-module reconfigurable tube with tunable section area

A bi-stable deployable tube with three identical stacking single-loop IMSS linkage modules $\mathrm{Loop}^{\mathrm{A}}, \mathrm{Loop}^{\mathrm{B}}, \mathrm{Loop}^{\mathrm{C}}$ is presented in Fig. S11(A$\sim$B) for the conceptual application of medical stents to demonstrate the basic idea. The coupling condition for stacking is $\Delta\theta_3^{\mathrm{L}A} = \Delta\theta_3^{\mathrm{L}B} = \Delta\theta_3^{\mathrm{L}C}$, which is implemented by the co-axis aligned modules and the rigid coupling frames between them. The deflection angle $\gamma_3$ and the deflection interval $\lambda_3$ of coupled hinge $\mathrm{H}_3$ are chosen based on the practical requirements for the local section shape, where it is set as $\gamma_3^{\mathrm{A,B}} = \gamma_3^{\mathrm{B,C}} = 0$ and $\lambda_3^{\mathrm{A,B}} = \lambda_3^{\mathrm{B,C}} = 0.3$m in this case. The physical model of the stacking tube is shown in Fig. S11(C). The stability is preserved as the IMSS linkage modules. The variation of its section shape is consistent with that of an IMSS linkage module as the deflection angles among the modules in the tube are zero. It is indicated in Fig. S11(D) that the arc length $\zeta_{1-3}$, $\zeta_{2-4}$ of hinges $\mathrm{H}_1 - \mathrm{H}_3$ and $\mathrm{H}_2 - \mathrm{H}_4$ expresses the opposite trend, which suggests the shape of the section comes from wide to narrow. The variation of the area projected at the direction of the axis of hinges $\phi_1, \phi_2, \phi_3$ follows the "U" type curve due to the wide range of angular variation, except that $\phi_4$ keeps increasing along the reconfiguration. To accomplish a more challenging reconfiguration of the section shape, one method is to redesign the IMSS linkage modules individually and ensure the coupling condition is satisfied as well. Yet, a simpler method is to utilize the deflection angle $\gamma_3$ of the adjacent modules as in Fig. S11(E) to customize the hollow rate $\eta(\gamma_3)$ and to adjust the fluid capacity of the tube. The hollow rate is defined as:

$$\eta(\gamma_3) = \phi_\wedge / \phi_\vee$$

where $\phi_\wedge$ is the intersection area of the projected section area of the stacking modules and $\phi_\vee$ is the union area, as shown in the figure. Considering a tube with two identical IMSS linkage modules $\mathrm{Loop}^{\mathrm{A}}, \mathrm{Loop}^{\mathrm{B}}$, the variation of the hollow rate along the transformation of the tube is shown in Fig. S11(F). The intersection area decreases with the deflection angle while the union area shows more complexity. The hollow rate decreases with the transformation in general, yet rises slightly at a certain range of deflection angle as the minor panel shows, where $\gamma_3 = 117°$.

### 3.2 Deployable tube with designable configurations

The deployment ratio of the N3-module deployable tube is roughly measured by defining the transverse contraction distance $\Delta\epsilon_1 = \epsilon_1^{\mathrm{S2}} - \epsilon_1^{\mathrm{S1}}$, and the longitudinal extension distance $\Delta\epsilon_2 = \epsilon_2^{\mathrm{S2}} - \epsilon_2^{\mathrm{S1}}$. Then the deployment ratio $\nu$ can be expressed as:

$$\nu = -\frac{\Delta\epsilon_1}{\Delta\epsilon_2}$$

In this design case, the deployment ratio is $\nu = 0.268$, while it could be designed by adjusting the parameters of the deflection angle $\gamma$ and the deflection interval $\lambda$ in the coupling frame. Fig. S12(A) plots a contour of the deployment ratio regarding these two designable parameters of Type II coupling frames, where a wide range of deployment ratio could be achieved. Further investigation about the effect of more coupled IMSS linkage modules is presented in Fig. S12(B). There is some sort of periodicity regarding deployment ratio because the tube would be deployed in a spatial spiral shape when coupling a large number of loops, as shown in Fig. S12(C). The code of analysis is included in Data S2 of Supplementary materials.

### 3.3 Reconfigurable robotics platform with circular coupling

Apart from the sequential geometric formation, some complicated applications such as robotics devices match advantageously with the circular coupling by multiple modules. Besides the closure conditions and coupling conditions of coupling hinges imposed in the similarity with the sequential coupling of stacking and chaining, one additional closure condition is derived from the hypothetical loop $\text{Loop}^{\text{O}}$ marked dashed lines in Fig. S13(A), which is generated by the relative location of the hinges in the surrounding IMSS linkage modules. In other words, it is practically unnecessary for the hypothetical loop to be fabricated as long as the relative location of the coupled-to-be hinges in the surrounding modules is subject to the closure condition. Instead, they are connected by the coupling frames (marked in green) directly for the aim of structural compactness.

To demonstrate more specifically, it is proposed a swimming robot in Fig. S13(B∼C). Inspired by the formation of frogs, four identical basic building blocks of IMSS linkage modules are coupled in the circular formation with a type of Bennett linkage at the center, by which the closure condition is naturally satisfied. Similarly, the center Bennett linkage provides the symmetric relative motion to four surrounded IMSS linkage modules. One diagonal pair of the modules acts as forelimbs to swing the fluid downwards and another pair is hindlimbs to push backward, which comes from the transformation from "stable state 2" to "stable state 1" of the 4R IMSS linkage modules. The reconfiguring direction angle of hinge $h$ of the Bennett linkage $\rho_h^{\text{L0}}$ is defined as the deflection angle between the local movement direction of hinges and the axis direction of the robot, as shown in Fig. S13(C), to characterize the thrust efficiency of the robot. Thus, the IMSS linkage modules coupled around would experience not only the non-rigid reconfiguration through the structural deformation of itself, but also the spatial transformation driven by the hinges of the Bennett linkage, as shown schematically in Fig. S13(B). The experimental snapshots are shown in Fig. S13(D).

Compared to the sequential coupling strategies of stacking and chaining, the Bennett linkage in the circular coupling is obviously obliged to satisfy more constraints and its design space is thus relatively narrow. The relation between the initial installation angles to the two sides of IMSS linkage modules and the designable parameters of the Bennett linkage is shown in Fig. S13(E). When one of the initial installation angles $\theta_{1,\text{init}}^{\text{L0}}$ of the Bennett linkage is selected, other parameter of $\theta_{2,\text{init}}^{\text{L0}}$ are determined, and thus the one-to-one mapping relation of the structural parameters $\alpha^{\text{L0}}$ and $\beta^{\text{L0}}$ is plotted as the bottom panel in Fig. S13(E). The influence on the reconfigured angle by the geometric properties of Bennett linkage is analyzed with its design space in Fig. S13(F), where each dot represents the reconfiguration process tracking by the rotation angle $\theta_1^{\text{L0}}$ of the center Bennett linkage with different structural parameters $\alpha^{\text{L0}}$. It is divided into four zones regarding the comparison of the direction angle in the forelimb IMSS linkage modules ($\text{Loop}^1$ and $\text{Loop}^3$) or the hindlimb modules ($\text{Loop}^2$ and $\text{Loop}^4$), which suggests the contribution difference of thrust providing by the modules. For the design with $0° < \alpha^{\text{L0}} \leq 90°$, the direction angle of the forelimb modules $\rho_1^{\text{L0}}$ is relatively lower than $\rho_2^{\text{L0}}$ of the hindlimb modules. The gap is slightly reduced at the range of $90° < \alpha^{\text{L0}} \leq 180°$, where $\rho_1^{\text{L0}}$ increases and $\rho_2^{\text{L0}}$ decreases correspondingly. The thrust provided by the forelimb modules is dominated at the range of $-90° < \alpha^{\text{L0}} \leq 0°$ as $\rho_1^{\text{L0}} > \rho_2^{\text{L0}}$, while the difference is diminished when $-180° < \alpha^{\text{L0}} \leq -90°$. The variation of the direction angle in the forelimb modules and the hindlimb modules of the design where the structural parameter $\alpha^{\text{L0}} = 73.1°$ is plotted at the bottom panel. As the reconfiguration proceeds, the direction angle $\rho_1^{\text{L0}}$ and $\rho_2^{\text{L0}}$ are both less than $90°$ at $\theta_1^{\text{L0}} = 109.3°$, which suggests the thrust would turn reversely after this point. Further analysis is conducted in Fig. S13(G∼H) with the variation of the projected area of the four IMSS linkage modules on the thrusting direction $\phi_1$ and $\phi_2$ (which are indicated to the projected area of the forelimb modules and the hindlimb modules respectively), and the sum of the volume

$\nu_1$ and $\nu_2$ displaced by the projected area in the transformation from the stable state 1 to the stable state 2. The neutral-contribution edge $\phi = 0$ is marked in Fig. S13(G). The projected area $\phi_2 = 0$ flips at $\hat{\theta}_1^{\mathrm{L0}} = 109.3°$ of the reconfiguration due to the reversed direction angle, where the displaced volume $\nu_2$ reach the maximum. This suggests that there is the process of acceleration (before $\hat{\theta}_1^{\mathrm{L0}}$) followed by deceleration (after $\hat{\theta}_1^{\mathrm{L0}}$) when the robot reconfigures. The whole reconfiguration is included in Movie S7.

# 4 Validation of shape accuracy, high stiffness, and lightweight design in IMSS linkages

## 4.1 Demonstration of the shape accuracy and high stiffness in IMSS linkages

In this section, quantitative comparison between an IMSS-R4 linkage and an over-constrained linkage (Bennett linkage) is conducted to emphasize the benefits of high shape accuracy, high stiffness, and high impulsivity with a lightweight actuator (SMA) and direct controlling strategies.

The two linkages are shown in Fig. S14(A). The IMSS-R4 linkage is same as in Fig. 2. At its two stable states $S_1$, $S_2$, the rotation angle of Hinge $H_1$ is prescribed as $\theta_1^{\mathrm{S1}} = 20°, \theta_1^{\mathrm{S2}} = 110°$. The angular variation of this hinge is thus: $\Delta\theta_1 = 110° - 20° = 90°$. Design parameters of the Bennett linkage are:

$$
\begin{array}{llll}
l_{12} = 4 & l_{23} = 4 & l_{34} = 4 & l_{41} = 4 \\
\alpha_{12} = 45° & \alpha_{23} = 120° & \alpha_{34} = 45° & \alpha_{41} = 120° \\
r_1 = 0 & r_2 = 0 & r_3 = 0 & r_4 = 0
\end{array}
$$

It could be verified that its DoF is equal to one, because it meets the structural condition of Bennett linkages[22]. The continuous configurations from $\theta_1 = 0°$ to $90°$ is schematically shown in Fig. S14(B).

Two SMA springs with the same stiffness is mounted at Hinge $H_1$ in the two linkages respectively, as shown in the Fig. S14(A). When imposing current in the circuit, SMA would retract the actuated hinge $H_1$ from the beginning angle $\theta_1^{\mathrm{BEG}}$ to the end angle $\theta_1^{\mathrm{END}}$. Three different amount of current, $I_a = 1.0\mathrm{A}, 1.5\mathrm{A}, 2.5\mathrm{A}$, are used for the actuation of the Bennett linkage, while the amount of current imposed on the IMSS linkage is equal to $I_a = 2.5\mathrm{A}$ at three independent tests, as shown in Fig. S14(C). The whole process of the comparison is included in Movie S2.

The impulse motion is observed in all three tests for the IMSS linkage. The angular increase could all complete in 2.0s. The angular increment at each time step in Fig. S14(D) indicates that the unchanged impulsivity in three tests. This is because the impulsivity of IMSS linkages comes from the intrinsic structural instability, and it is not affected by the property of the actuator, as long as the reaction force of the SMA spring is large enough to actuate the hinge. Correspondingly, a high speed of reconfiguration for the Bennett linkage is also observed when the amount of current is 1.5A and 2.5A. The speed of these two reconfiguration exceeds that of IMSS linkages. However, this impulsivity in the Bennett linkage highly depends on its actuator. It suggests that impulsivity could also be realized in Bennett linkages, only if the current could increase to a rather large amount. Otherwise, the reconfiguration would be much slower than the IMSS linkage, like the case with the current of 0.5A. It is noted that, the increased stiffness of the SMA actuator with a larger reacting or retracting force could also enhance the impulsivity for the similar reason, which would not elaborated here.

The result of shape accuracy is characterized by the rotation angle of Hinge $H_1$, which is listed in Fig. S14(E). The shape accuracy is characterized by the target deviation of the rotation angle at the end state, $\delta_t = \Delta\theta_1^{\mathrm{END}}/\theta_1^{\mathrm{END}} = \|\theta_1^{\mathrm{END}\prime} - \theta_1^{\mathrm{END}}\|/\theta_1^{\mathrm{END}}$, where the target $\theta_1^{\mathrm{END}} = 90°$ in this case, and $\theta_1^{\mathrm{END}\prime}$ is the real value of the angle after actuation. A higher accuracy of the rotation angle is observed in these three experiments, which is all below 10%. Comparably, the target deviation is much higher in the Bennett linkage, where $\theta_1$ remains at $15°$ when $I_a = 0.5\mathrm{A}$.

The shape accuracy of the Bennett linkage could be enhanced by increasing the complexity of controlling strategy. As shown in Stage I in Fig. S14(F), the angular target of the reconfiguration is to actuate Hinge $H_1$ rotating from $0°$ to $90°$ by one SMA actuator. As the circuit is connected at the beginning, $\theta_1$ would be

increased from $\theta_1^{\text{BEG}} = 0°$ due to heating. When it is observed to be equal to the target angle, $\theta_1^{\text{END}} = 90°$, the circuit is manually switched off at Stage II, which is equivalent to a close-loop controlling method. However, the hinge would not be preserved at this angle at this stage, because the shape of SMA springs is sensitive to the temperature, which would be slowly decreased if stopping heating. The SMA spring would finally stop at a random length, where the heat transfer with air stops, and $\theta_1^{\text{END}}$ is thus undetermined as well. Therefore, the temperature control of SMA actuators is crucial to the enhancement of the shape accuracy. If without this closed-loop control of its temperature as in Stage III, $\theta_1$ would continue to increase until the spring is fully retracted, and then stop at the undesired state with a rather low shape accuracy. The whole process is included in Movie S2.

The stiffness of the two linkages is compared quantitatively by pulling tests on the same hinge $H_1$ with cable. The component pulling force along the cable is recorded as in Fig. S14(G). It indicates that higher force is needed to actuate the IMSS linkage to the prescribed configuration, which suggests there is a higher stiffness for IMSS linkages to preserve the configuration, comparing to over-constrained linkages.

Collectively, the result in the series of experiments validates that: 1) Impulsivity could be achieved in both IMSS linkages and over-constrained linkages, yet the impulsivity in IMSS linkages is intrinsic and not affected by the property of actuators, and could be possible be realized with lower power consumption. 2) Higher shape accuracy is observed in the reconfiguration of IMSS linkages, while the shape accuracy in over-constrained linkages is highly associated with the accuracy of the actuator. 3) More external force of perturbation is needed to make the shape deviation of IMSS linkages, which suggests a higher stiffness to preserve its current configuration.

### 4.2 Demonstration of the lightweight design in IMSS linkages

In this section, IMSS linkages are compared with flexible mono-stable structures, 1-DoF mechanisms, n-DoF mechanisms (n>1), to demonstrate its merits of lightweight design.

Flexible mono-stable structures could be used to construct two available states in some special applications such as deployable solar panel design, as shown in Fig. R1 [40], where it could be deformed and folded in the limited space of the payload fairing of the rocket, with tied by cables, and then released and deployed when arriving at the setting orbit in space. In this case, there needs only one actuator to release the cable, and then the deployment would be conducted automatically by the potential energy (in some cases by motors to avoid the vibration). However, it would be tricky when requiring the retractment of the deployable device as in the retractable solar array [41]. More actuators are needed in this retracting mission. As a comparison, if adopting bi-stable structures in this case, such as IMSS-R4S2, it could complete the forward and reverse reconfiguration with only two actuators, and thus the claimed advantage of lightweight over the mono-stable structure is validated.

Some 1-DoF mechanisms are used to design this retractable solar array. For example in the spacecraft Space Flyer Unit launched in 1995 [41,42], the Miura-ori pattern is used in its solar array design, as shown in Fig. R2(A), and theoretically, it could be accurately actuated forward and reversely by one close-loop motor due to its single DoF [43], as shown in Fig. R2(B). In other words, the reconfiguration among its configuration space could be accurately controlled by one accurate actuator, such as the encoded close-loop motor. Therefore in this case, multi-stable systems, such as IMSS linkages, would not pose a great advantage of lightweight actuation to 1-DoF mechanisms, though the formation of the actuators in IMSS linkages could be simple and inaccurate as SMA, and its design space of IMSS linkages is generally expanded, no more talking that there needs more sets of actuators in IMSS-R6S4 linkages and other complex IMSS assemblies.

Besides the Miura-ori pattern, some folding patterns of n-DoF (n>1) mechanisms are also used in deployable device designs. As shown in Fig. R3, the Resch-like pattern [44], which has multiple DoF (84-DoF for the presenting tessellation), is proposed to construct a reconfigurable surface. Each DoF of the mechanism needs to be controlled by one close-loop motor, to accurately drive the reconfiguration among the target states, thus 84 actuators in total, compared to IMSS-R6S4 linkages, where only four simple actuators are needed in the four-target reconfiguration. Therefore, IMSS linkages would pose the advantage of lightweight over this n-DoF mechanism.

Here is a series of works to express this idea systematically. As the reconfigurable antenna proposed in this work [45], four shape configurations are required to be accessible when the antenna is operating in space. As the constrained design space of 1-DoF mechanisms, it would be difficult to design inversely a 1-DoF mechanism that is able to cover all four target shapes, i.e. the target configurations are generally not included in its configuration space. A 12-DoF reconfigurable origami pattern is proposed in [36]. It is accessible to these four configurations, yet it is facing the issue of higher quantities of actuators. The solution is to design a multi-stable surface. A surface based on bi-stable element assembling [39,46] is proposed to fulfill the requirement of reconfiguration. It only needs three actuators (two cables and one slider) to drive the reconfiguration among the target surfaces, whereas there are many redundant intermediate stable states in this bi-stable-unit assembly as discussed in Sec. 1 of the main content (which validates the benefits of being intrinsically multi-stable over being assembled by bi-stable units).

Collectively, IMSS linkages hold the advantage of lightweight design only when it is in a specific situation, where it is required that the quantity of the accessible configurations is finite, and also they are not all included in the configuration space of a 1-DoF mechanism (but it is generally hard to know whether they are included or not).

| **Type** | **Kinematic DoF** | **Disadvantages compared to IMSS linkages** | **Advantages compared to IMSS linkages** |
|---|---|---|---|
| Flexible mono-stable structures | =0 | More actuators for the accurate reversible reconfiguration. | One-direction reconfiguration driven by one actuator. |
| 1-DoF mechanisms | =1 | Constrained design space. (Targets are generally inaccessible.) | One actuator for the accurate reversible reconfiguration. |
| n-DoF mechanisms (n>1) | >1 | More actuators for the accurate reversible reconfiguration. | Expanded design space. |

### 4.3 Demonstration of the reversible reconfiguration of IMSS linkages with simple actuators

In this section, reversibility in the reconfiguration of IMSS linkages is validated by engaging the least quantities of actuators (two sets of actuators for bi-stable linkages, four sets for quadra-stable linkages).

For bi-stable linkages, including the IMSS-R4S2 linkage and the impulsive gripper, there only needs two sets of angular actuators to complete the forward and reverse reconfiguration respectively. As shown in Fig. S15(A), two SMA actuators, SMA A and SMA B, are implemented in two hinges, Hinge 1 and Hinge 4, to actuate $\theta_1$ and $\theta_4$. Heating SMA materials could increase the corresponding angles. Specifically, as shown in the upon right, the deformation of SMA A engages the forward reconfiguration from Stable state $S_1$ to Stable state $S_2$, while as in the below right, the deformation of SMA B engages the reverse reconfiguration from Stable state $S_2$ to Stable state $S_1$. The complete process could be found in Supplementary Movie S2. Similarly in the impulsive gripper, two sets of actuators are implemented as shown in Fig. S15(B), where the SMA spring in the above row would retract to decrease the angle when heating, and the SMA flake in the below row would elongate to increase the angle when heating. The two actuators could control the forward and reverse reconfiguration respectively. The complete process could be found in Supplementary Movie S5. It should be noted that, though the least quantity of actuators for the reversible reconfiguration of bi-stable linkages is two, there needs only one active actuator in each one-direction reconfiguration.

For quadra-stable IMSS-R6S4 linkage, as discussed in "2.2 Quadra-stable single-loop spatial 6R linkages" and Fig. 3 of the manuscript, there are two angles to be individually controlled to complete the reverse reconfiguration of "$S_1$-$S_2$-$S_3$-$S_4$-$S_1$". Therefore, there needs two sets of actuators for each hinge control, and four sets of actuators in total. As shown in Fig. S16, two sets of SMA actuators are implemented at Hinge 2 to increase and decrease $\theta_2$, while $\theta_5$ is actuated forward and reversely by a cable linked on Hinge 5 with the fixed direction. Specifically, in the sub-process from Stable state $S_1$ to Stable state $S_2$ as shown in Fig. S16(A), the SMA actuator in the Hinge 2 is active by heating, while keeping $\theta_5$ unchanged by inactivating the actuator on Hinge 5. In the sub-process from Stable state $S_2$ to Stable state $S_3$ as shown in Fig. S16(B), $\theta_5$ is decreased as pulling the cable linked on Hinge 5, and actuators in Hinge 2 are inactive. Similarly in

the sub-process from Stable state $S_3$ to Stable state $S_4$ in Fig. S16(C), another SMA actuator is implemented at Hinge 2 and heated to actuate $\theta_2$, and then Hinge 5 is pulled back with another fixed direction in the sub-process from Stable state $S_4$ to Stable state $S_1$ shown in Fig. S16(D), which is the stable state at the beginning. The complete process could be found in Supplementary Movie S3. Therefore, the reversibility of the reconfiguration with four actuators is validated.

## 5 Stiffness analysis for IMSS-R4S2 linkage modules and alternative designs

This section is divided into two main contents, which are the target proximity analysis for the constrained optimization solver of IMSS-R4S2 linkages and the alternative design based on the invariance principle of the hinges sliding strategy.

As shown in Fig. 17(A), two stable states are prescribed with the rotation angle $\theta^{S_1}, \theta^{S_2}$, which acts as the target input fed into the constrained optimization solver. The limit of the average design error $\mathrm{e}(\delta_{\theta 1})_{\text{max}} = 1/2 \cdot (|\theta^{S_1} - \theta^{\hat{S}_1}| + |\theta^{S_2} - \theta^{\hat{S}_2}|)$ is specified in the target proximity constraint of the solver. As the limit increases, the bi-stable 4R structure generated by the solver, naturally, suffers the higher proximity error, and yet correspondingly, as shown in Fig. 17(B), possesses the higher overall stiffness property due to the fewer constraints on the proximity. It shows the compensation mechanism between the proximity and the stiffness property. The inclination of regression on the proximity error of bi-stable 4R structures is observed when feeding the opposite target rotation angle ($\theta^{S_1} + \theta^{S_2} = 0$), where a type of symmetric bi-stable structure based on the snap-through phenomenon is requested to be generated as prescribed. The proximity error of opposite targets is conspicuously higher than in other cases when the error limit $\mathrm{e}(\delta_{\theta 1})_{\text{max}}$ is lower than 4%, otherwise, it is decreasing to an obviously low level compared to others. A similar tendency is presented in the stiffness analysis, except that it comes to an equivalent position when the error limit is high. It suggests that it is more inclined to fall into the inherent solution of the symmetric bi-stable structure within this highly nonlinear design space.

Not all the direct solution of IMSS linkage modules is physically feasible. The self-interference problem is the obstruction to fabrication as shown in Fig. 17(C), yet it is possible to be avoided by the hinges sliding strategy based on the invariance principle (See "1.2.4 Self-interference detection for theoretical models of IMSS linkage module" of Supplementary text). The configuration of a linkage, which is expressed as the relative position of its hinge axes, is unchanged after sliding in the view of the D-H notation, because the sliding direction is consistent with the hinge axes. However, the variation is introduced in the shape of the connected tetrahedrons. The shape and the stiffness property of the whole IMSS linkage module are consequently changed as shown in Fig. 17(D). With three different groups of the given sliding distance of $h2$ and $h3$ acting on the original IMSS-R4S2 linkage module in Fig. 2(A), $\{\mathrm{D}_{h2}, \mathrm{D}_{h3}\} = \{-0.3, -0.6\}, \{0.0, 0.0\}, \{0.3, 0.6\}$, the increased stiffness and the increased projected area of the module are characterized by the variation of the other hinge sliding distance. There is a trade-off in the stiffness and consistency in the projected area between the stable state $S_1$ and stable state $S_2$ as the hinge $h_1$ and hinge $h_2$ slide. The effective sliding is enclosed by the rounded rectangles to indicate the sliding results with no self-interference. It is noticed that the positive sliding to the hinge $h_2$ and hinge $h_3$ brings a wider range of results without self-interference. All cases (in blue) and the cases without self-interference (in red) are compared in Fig. 17(E).

The range of the cases without self-interference shrinks comparably. The achievable stiffness is at a slump, and the Pareto front of the increasing area domain is retreated, while the Pareto front of the decreasing area domain remains the same. It suggests the appropriate sliding distance improves the original structure but the blind increase in the sliding makes no contribution to the self-interference avoidance, yet conversely causes the deterioration of the stiffness property. It is noted that the self-interference avoidance strategy is not included in the constrained optimization solver for the inverse design of IMSS linkage modules, out of the consideration of computing efficiency. Empirically, it works well if the two stable states of IMSS-R4S2 linkages are examined to be self-interfered, but it needs to be improved for more complicated cases.

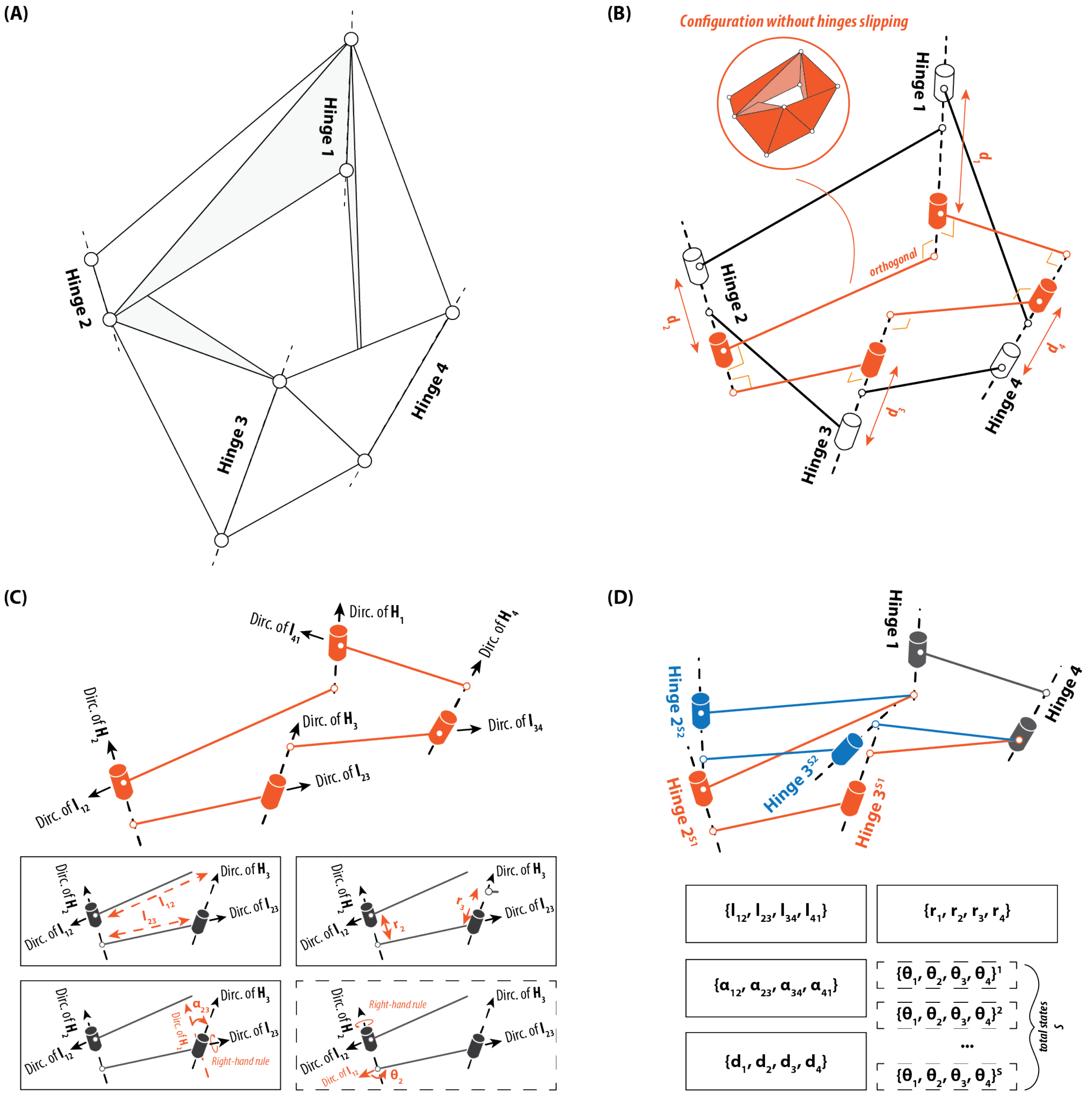


**Fig. S1. Parameterization block with D-H notation.** (**A**∼**B**) Parameters of hinges sliding in IMSS-R4 linkages. The original position of its hinges is presented as the dark colors while the axes are fixed naturally. The line that is vertical to the adjacent original hinges is indicated as the orange solid line with the intersection marked as the updated hinges in orange. The interval is the sliding distance. (**C**) Illustration of the structural parameters and the kinematic parameters. The positive direction of the hinge axis and the connection links is prescribed, and is subject to the right-hand rule. (**D**) Parametrization of IMSS linkages. It is based on the hypothesis that the structural parameters stay identical yet the kinematic parameters are changeable at the different states, which suggests it is sufficient to express a IMSS linkage with merely one group of structural parameters and S (the quantity of the prescribed state) groups of kinematic parameters.

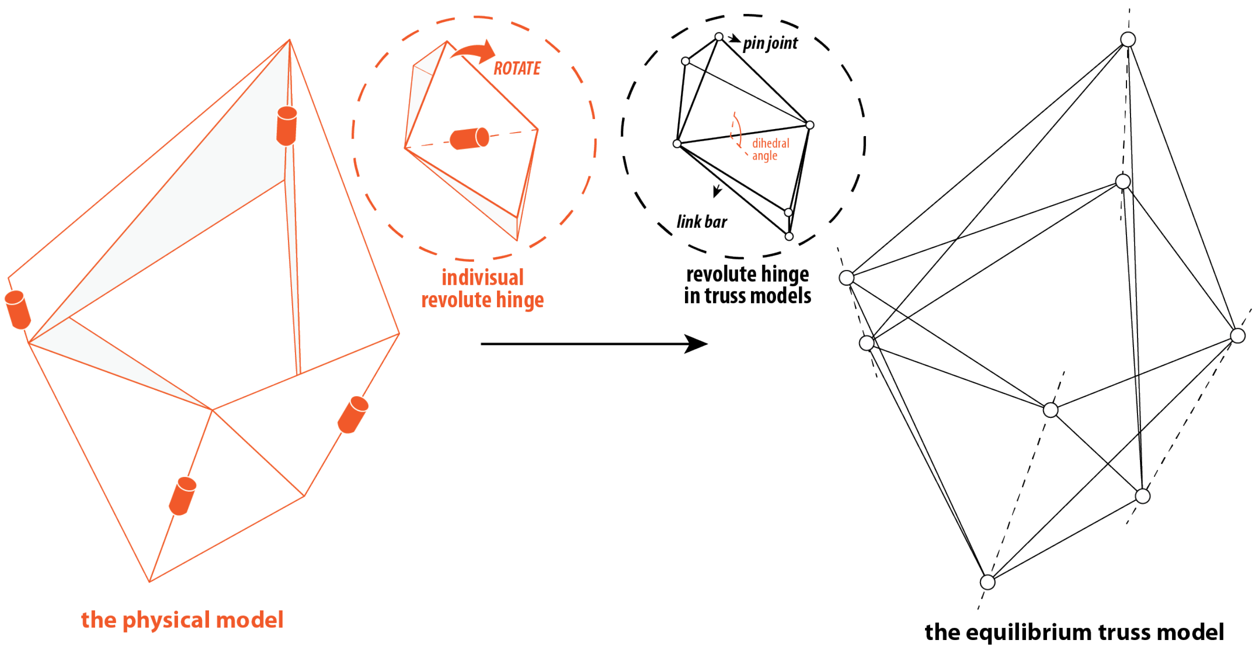


**Fig. S2. Truss model for IMSS-R4S2 linkages.** The original revolute hinges in IMSS-R4S2 linkages are treats as a truss model of pin joints and link bars. The compatibility matrix can thus be established to analyze its kinematic property.

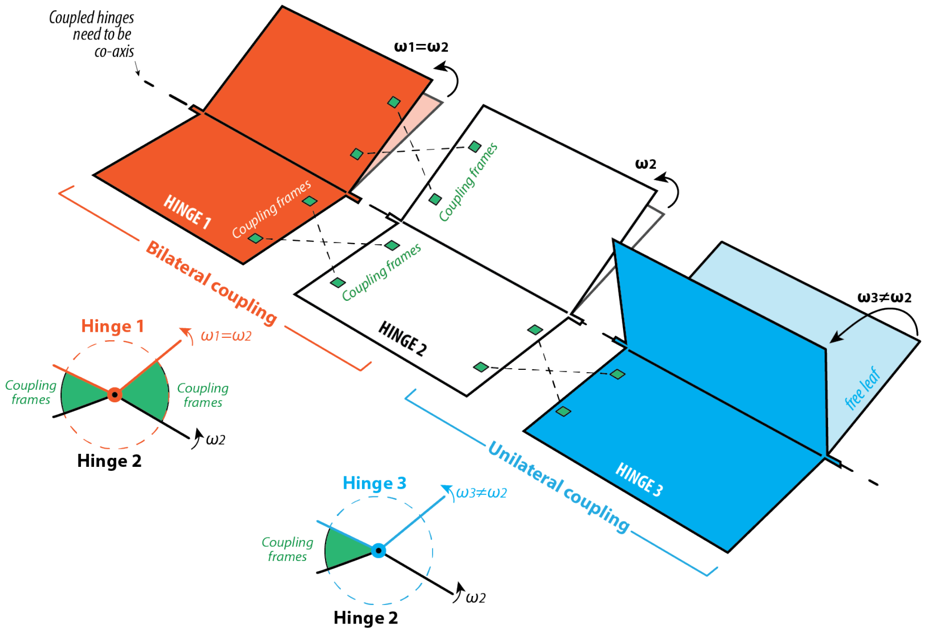


**Fig. S3. Coupling strategies for revolute hinges.** There are two parts of the strategy: be co-axis and be bilateral coupled, where the former one is a general requirement to make the coupling hinges physically feasible, and the last one makes the coupling hinges rotate simultaneously. If the last requirement is violated as shown in the blue hinge and the original pale hinge, the additional degree of freedom is introduced as the rotation of two hinges is separated and synchronous.

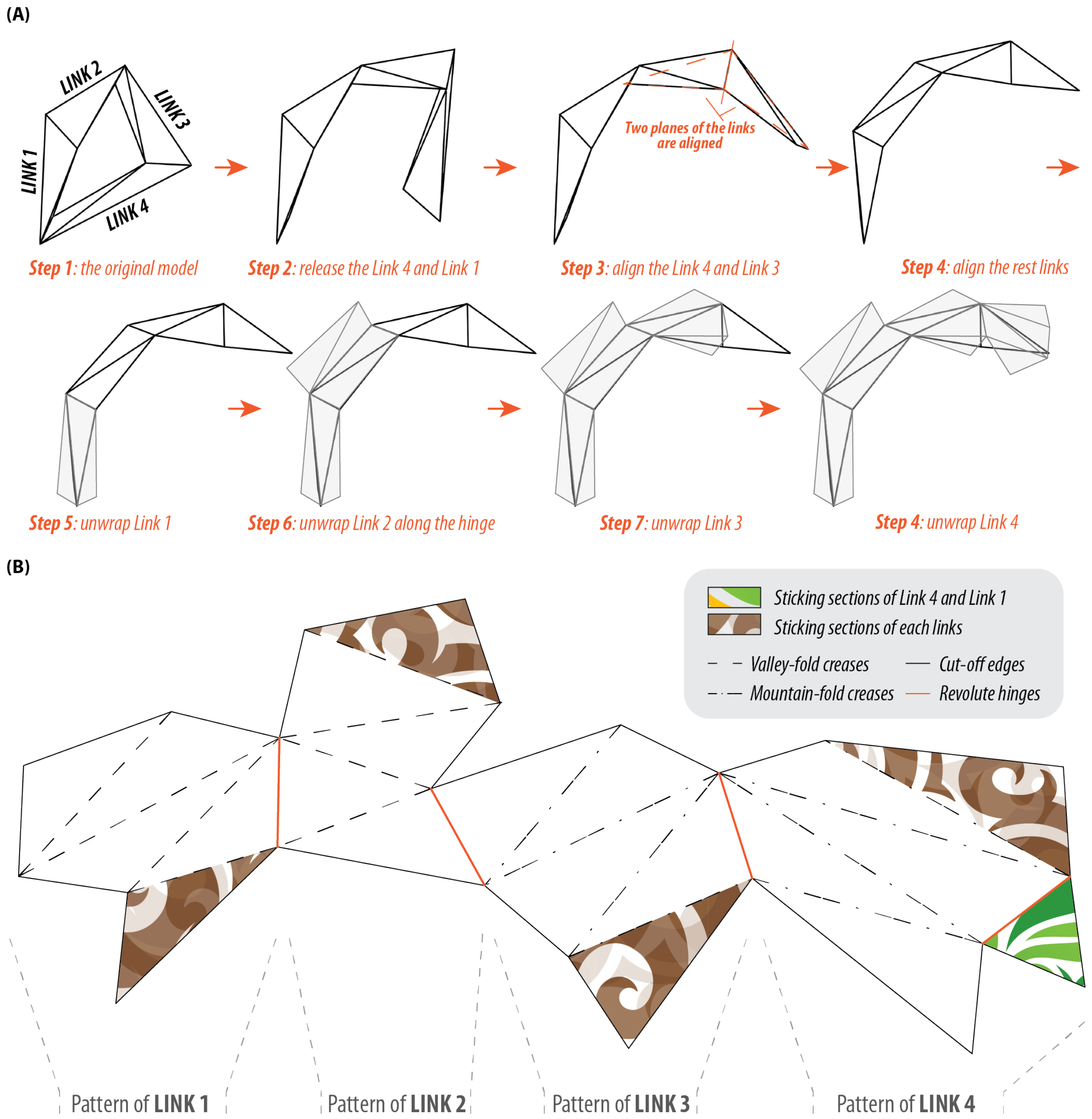


**Fig. S4. Unwrapping the IMSS-R4S2 linkage.** (**A**) Procedure of unwrapping. Release the closed loop and align them, and then unwrap each links. (**B**) The flat pattern of the 4R IMSS linkage in the main content case.

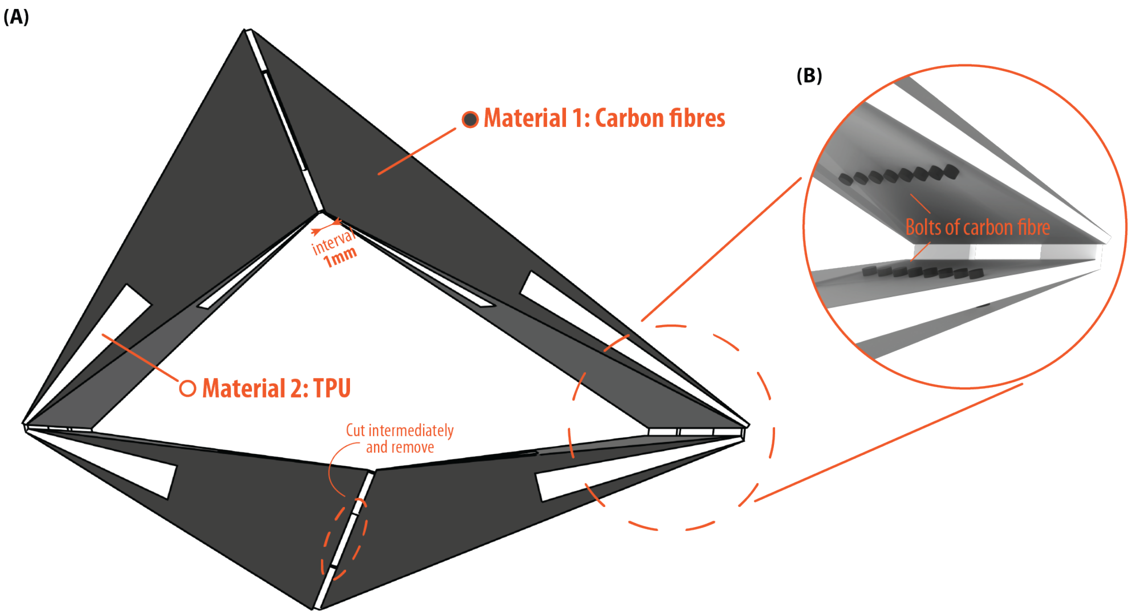


**Fig. S5. Detailed physical model design for bi-materials printing.** (**A**) Visual models are designed in advance with a CAD software. Two materials are appointed to the different parts of the model, as the dark parts to that of carbon fibers and the light parts to that of TPUs. To make hinges rotate shaper and smooth, the narrow interval (which is 1 mm in this case) and the section in hinges is reserved on purposes. (**B**) Bolts made of carbon fibers are derived from link and insert through the hinges of TPU. They are able to fix two different materials.

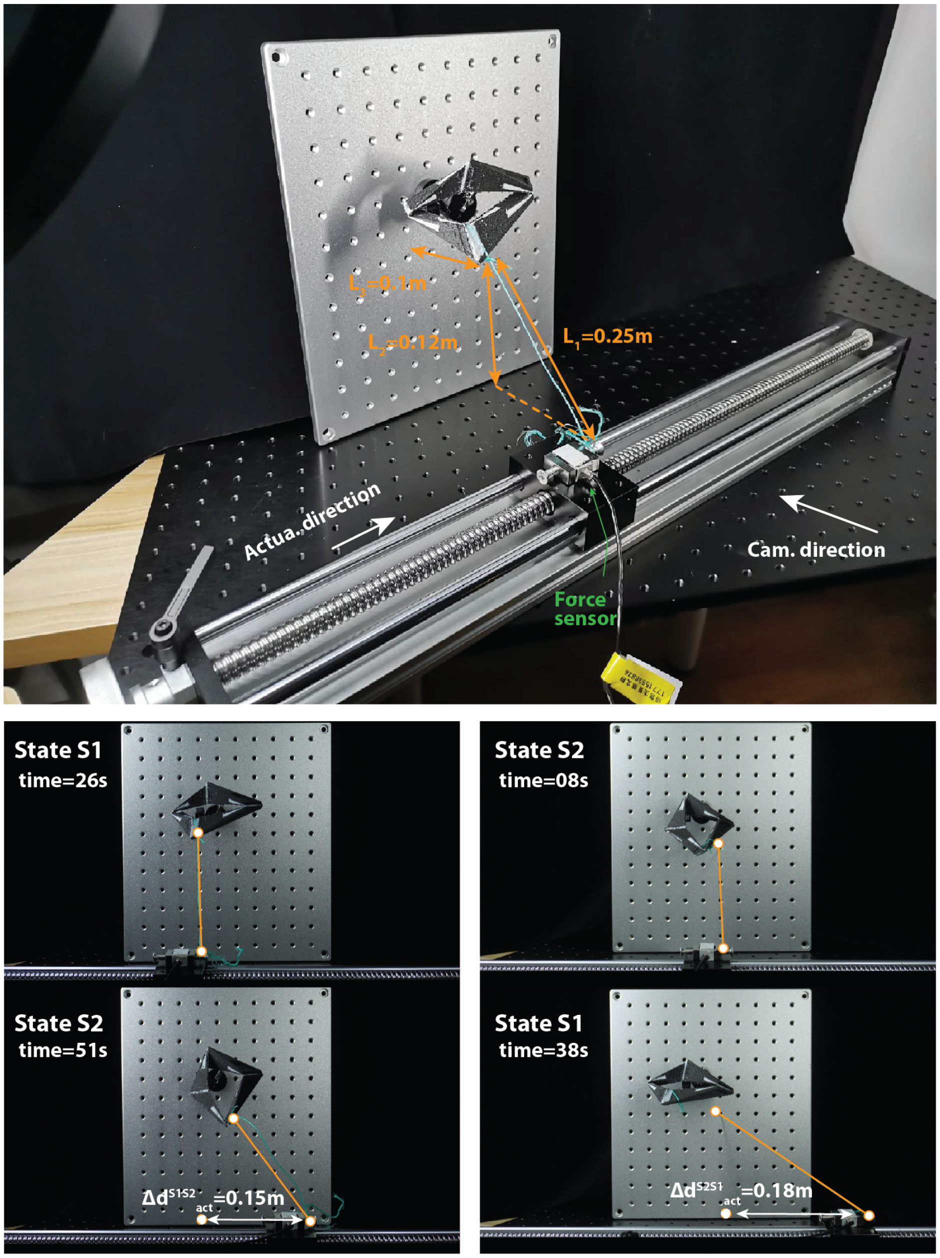


**Fig. S6. Experiments preparation.** The geometric relation is appointed in the figure, thus the distance of actuation on the trail and on the structure can be calculated.

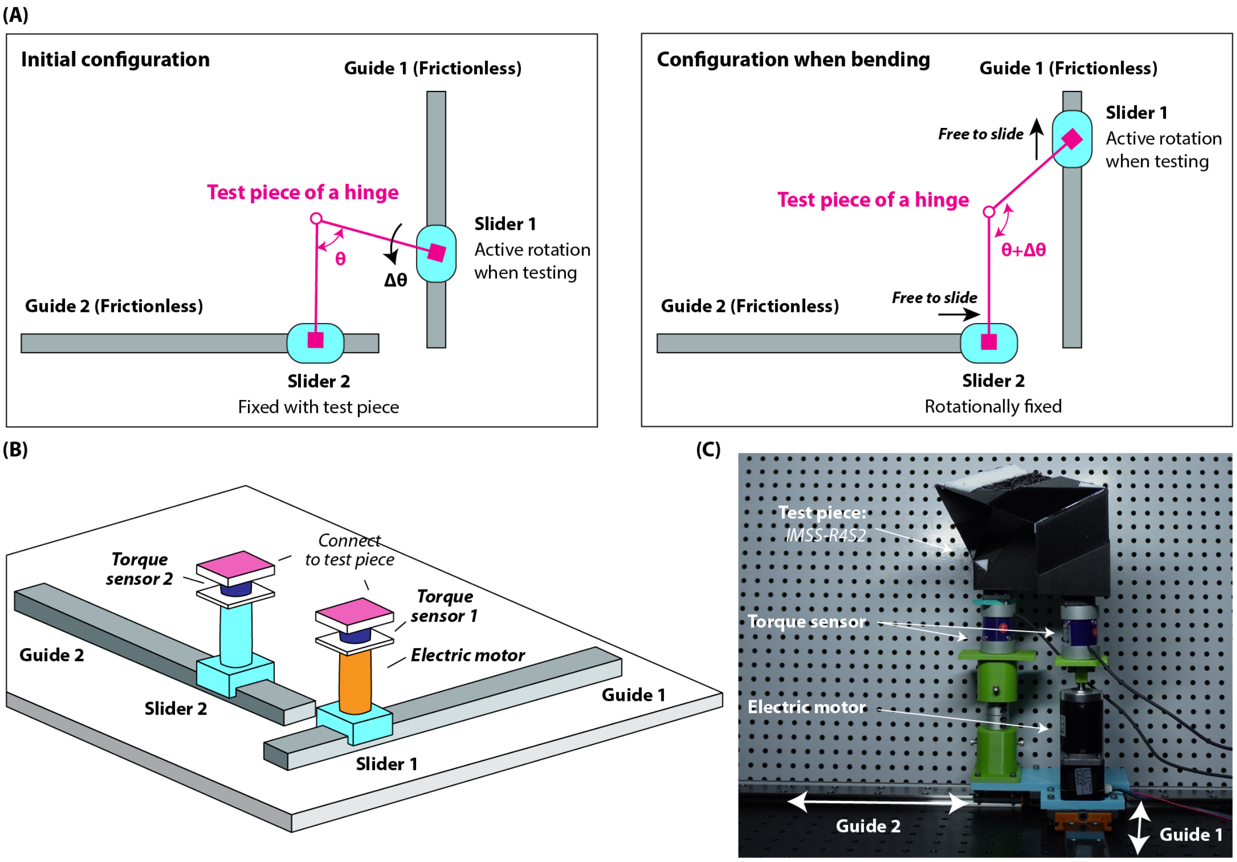


**Fig. S7. Pure bending machine[39].** (**A**) Conceptual illustration of the pure bending machine where two sliders can slide freely on two perpendicular guides. As the electric motor on Slider 1 rotates active at $\Delta\theta$, the testing hinge could be changed at the same angle. (**B**) Layout of the pure bending machine. (**C**) Physical model of the pure bending machine, connecting to a IMSS-R4S2 linkage to test. The testing result is given in Fig. 2(I).

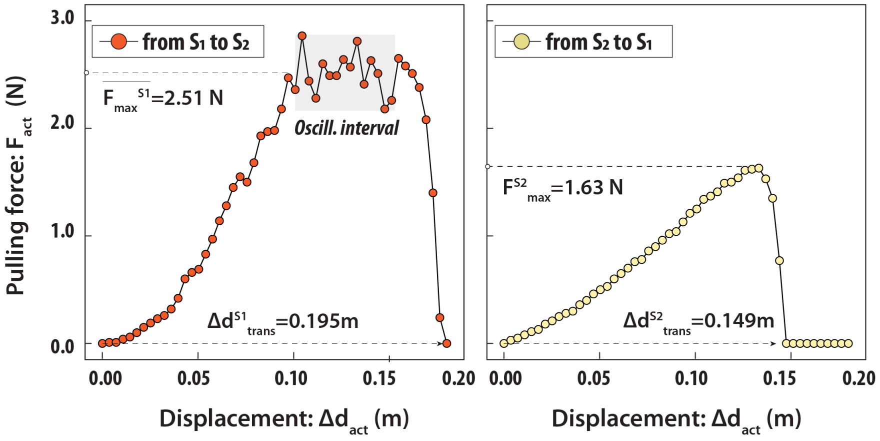


**Fig. S8. Force-displacement curve of the IMSS-R4S2 linkage.** The highest actuating force of the process from Stable state $S_1$ to Stable state $S_2$ is $F_{\max}^{\mathrm{S}_1} = 2.51$ N, while $F_{\max}^{\mathrm{S}_2} = 1.63$ N. The testing video is shown in Supplementary movie S2.

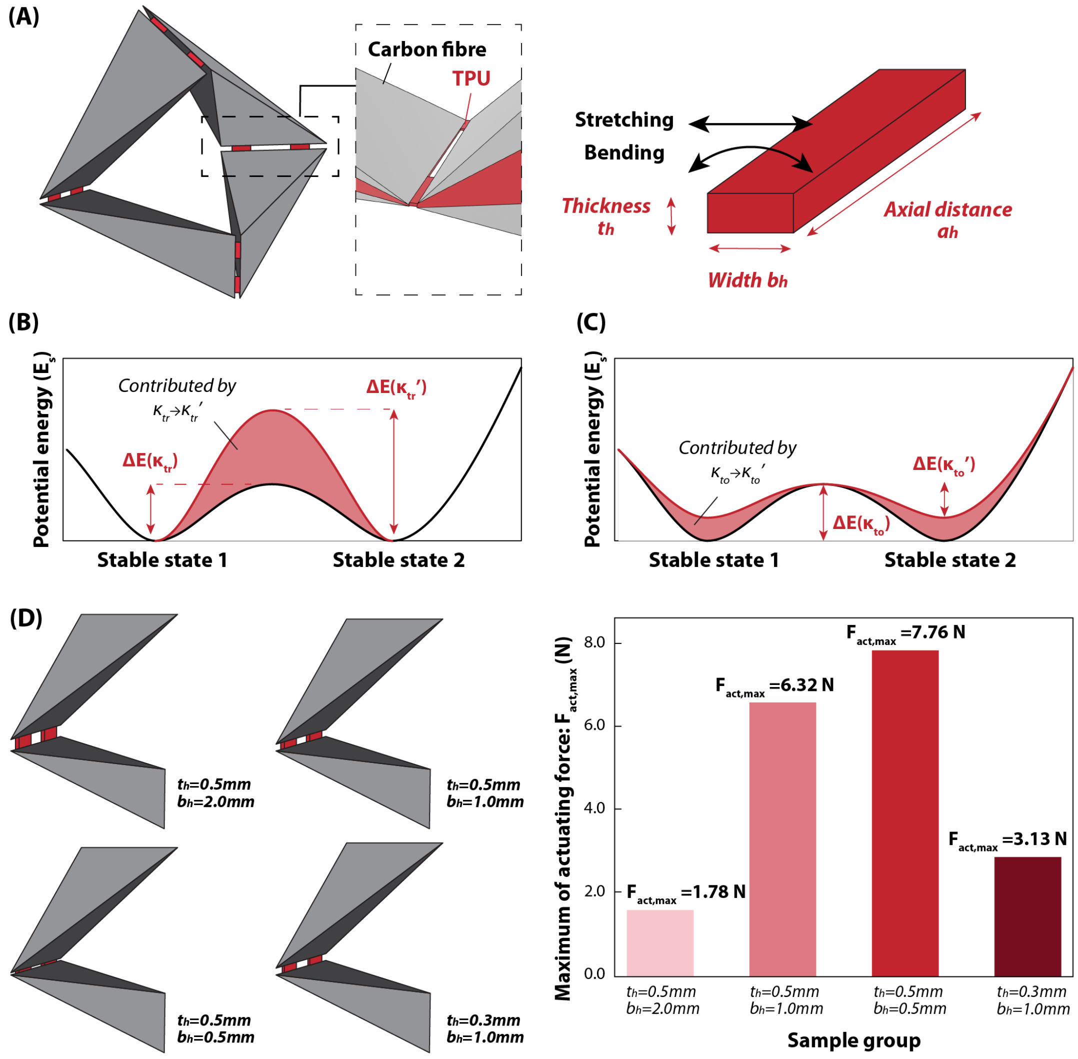


**Fig. S9. Stiffness analysis of a single TPU hinge.** (**A**) Equivalent model of a single TPU hinge. (**B**) Change of the energy landscape due to the increased stiffness ($\kappa'_{tr} > \kappa_{tr}$) of the equivalent translational spring. (**C**) Change of the energy landscape due to the increased stiffness ($\kappa'_{to} > \kappa_{to}$) of the equivalent torsional spring. (**D**) Maximum actuating force of different properties of hinges. The experimental set-up is presented in Fig. S6. The video of testing is included in Supplementary movie S2. It suggests the positive correlation between the stiffness of the IMSS-R4S2 linkage and the connection strength of hinges. Though there is an additional friction and angular offset of pulling force as the actuation, this positive correlation is still valid, because they are two common effect to all samples.

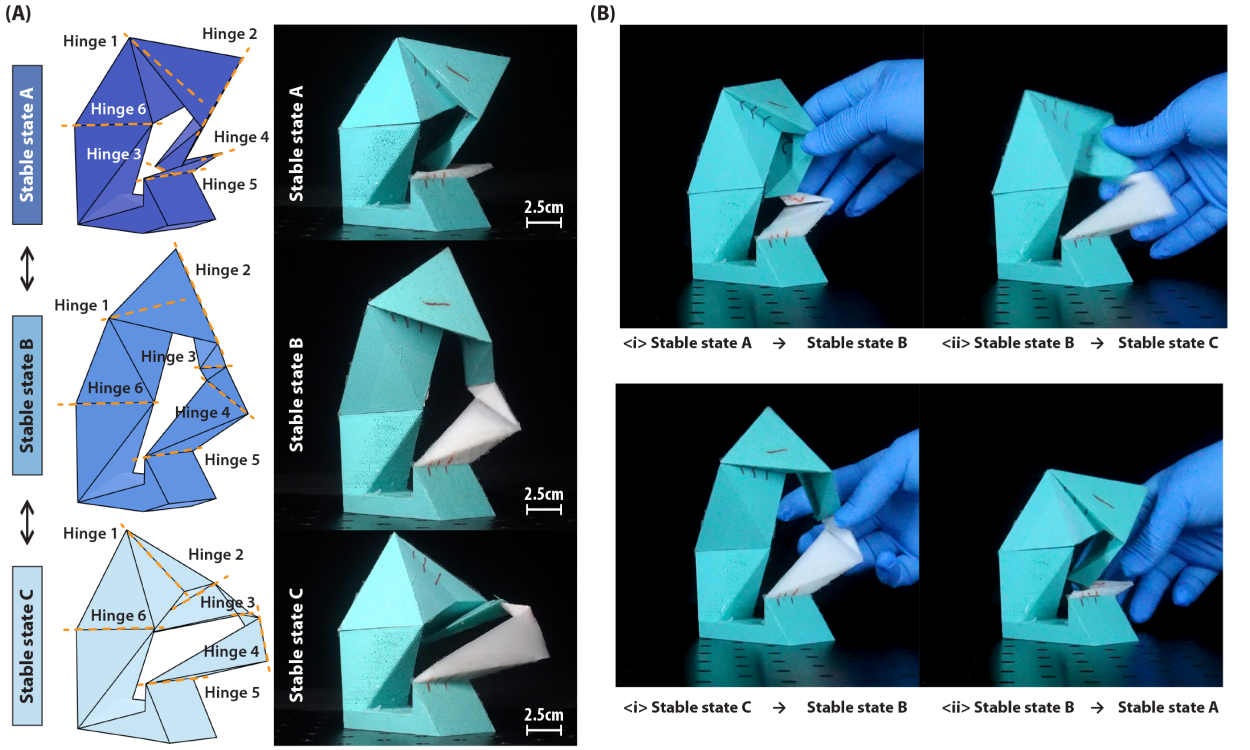


**Fig. S10. Design of a IMSS-R6S3 linkage.** (**A**) Schematic and physical models at three stable states are exhibited respectively. (**B**) The snapshots of the transformation are presented. The whole process is shown in Supplementary movie S6.

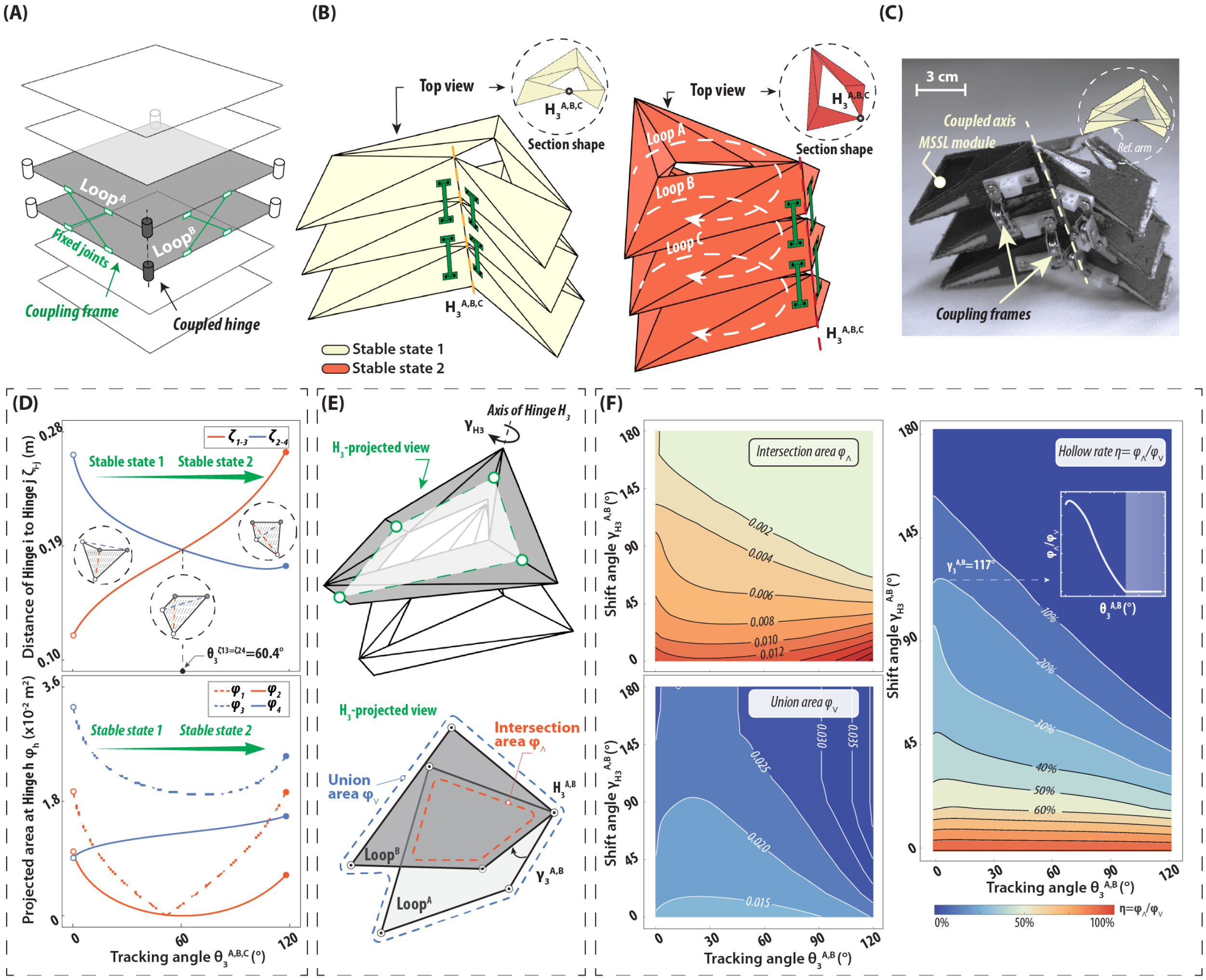


**Fig. S11. Demonstration of the deployable tube with tunable spatial configurations.** (**A**) Stacking coupling strategy. (**B**) Schematic of a 3-module stacking IMSS linkage: bi-stable reconfigurable tube. Three identical IMSS linkage modules are placed co-axis with the zero-deflection angle. (**C**) The physical models of the tube. The components of IMSS linkage modules and coupling frames are indicated. (**D**) Projected shape analysis of the consistent stacking IMSS linkage. The arc length $\zeta_{1-3}$, $\zeta_{2-4}$ and the projected area $\phi_h$ to hinge $\mathrm{H}_h$ is variated with rotation angle $\theta_3^{\mathrm{A,B,C}}$ . (**E**) Definition of the intersection area $\phi_\wedge$ and the union area $\phi_\vee$ of the tube section. The hollow rate $\eta(\gamma_3) = \phi_\wedge/\phi_\vee$, is defined to characterize the ability to transmit the fluid through. (**F**) Analysis of the hollow rate to the deflection angle $\gamma_3$. When $\gamma_3 = 117°$, the hollow rate rises slightly before the reduction at the reconfiguration, as shown in the minor panel.

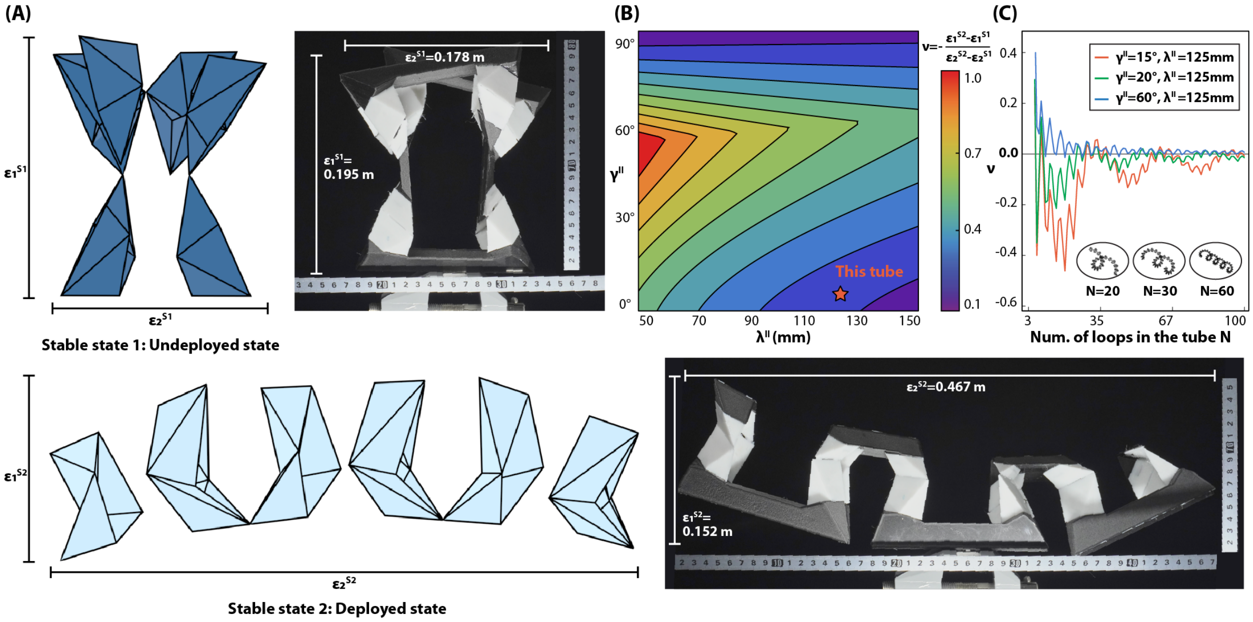


**Fig. S12. Demonstration of stacking coupling of IMSS linkage modules: a deployable tube with tunable spatial configurations.** (**A**) Measurement of the transverse contraction distance and the longitudinal extension distance of the deployable tube. The physical deployment ratio of the tube is 0.1488, while the theoretical result is 0.2687. The deviation mainly comes from the fabrication error. (**B**) Effect to the deployment ratio regarding the deflection angle $\gamma^{\mathrm{II}}$ and deflection interval $\lambda^{\mathrm{II}}$ of Type II frames. A wide range of deployment ratio could be designable by regulating $\gamma^{\mathrm{II}}$, $\lambda^{\mathrm{II}}$. (**C**) Effect on the deployment ratio regarding the quantity of coupling modules. There is some sort of periodicity regarding the deployment ratio. The simulation of the construction with a large number of loops is in Data S2 of Supplementary materials.

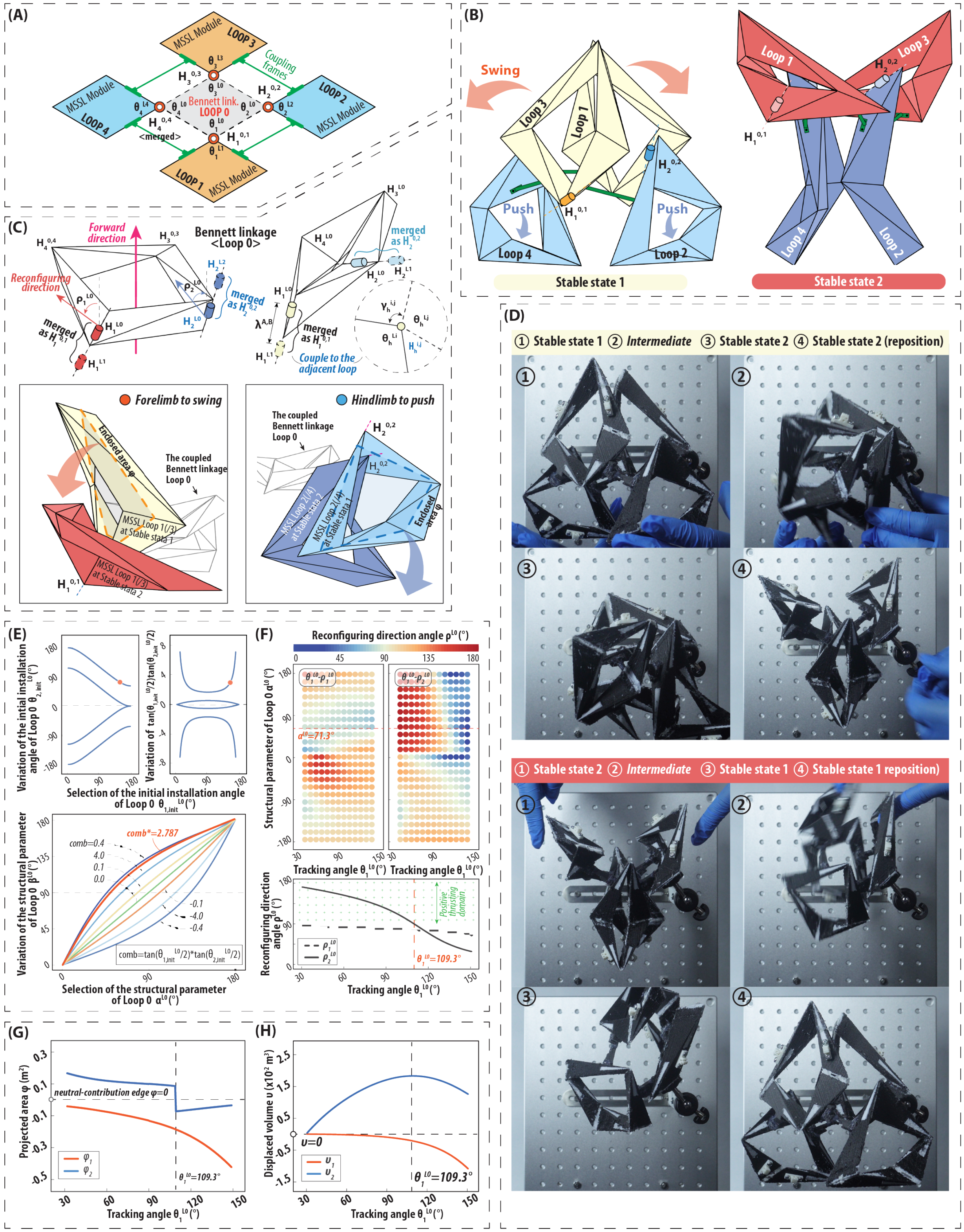


**Fig. S13. Demonstration of circular coupling of IMSS linkage modules: a swimming robot.** (**A**) Four loops $l = 1, 2, 3, 4$ are coupled circularly by the center loop at coupled hinges $\mathrm{H}_l^0$, and merged as $\mathrm{H}_l^{0,l}$. (**B**) Model of the integrated swimming robot. Four identical IMSS linkage modules are coupled with a Bennett linkage loop. (**C**) Schematic of the motion of the robot. The direction angles $\rho_1^{\mathrm{L0}}$ and $\rho_2^{\mathrm{L0}}$ of the Bennett linkage indicate the spatial transformation of the IMSS linkage modules. (**D**) Experimental snapshots. (**E**) Design space of the Bennett linkage. (**F**) Analysis of reconfiguring direction angles $\rho_i^{\mathrm{L0}} (i = 1, 2)$ to the structural parameters $\alpha^{\mathrm{L0}}$. The variation of the direction angle when $\alpha^{\mathrm{L0}} = 71.3°$ is shown below. (**G**$\sim$**H**) Analysis of the projected area $\phi_h$ and the displaced volume $\nu_h$.

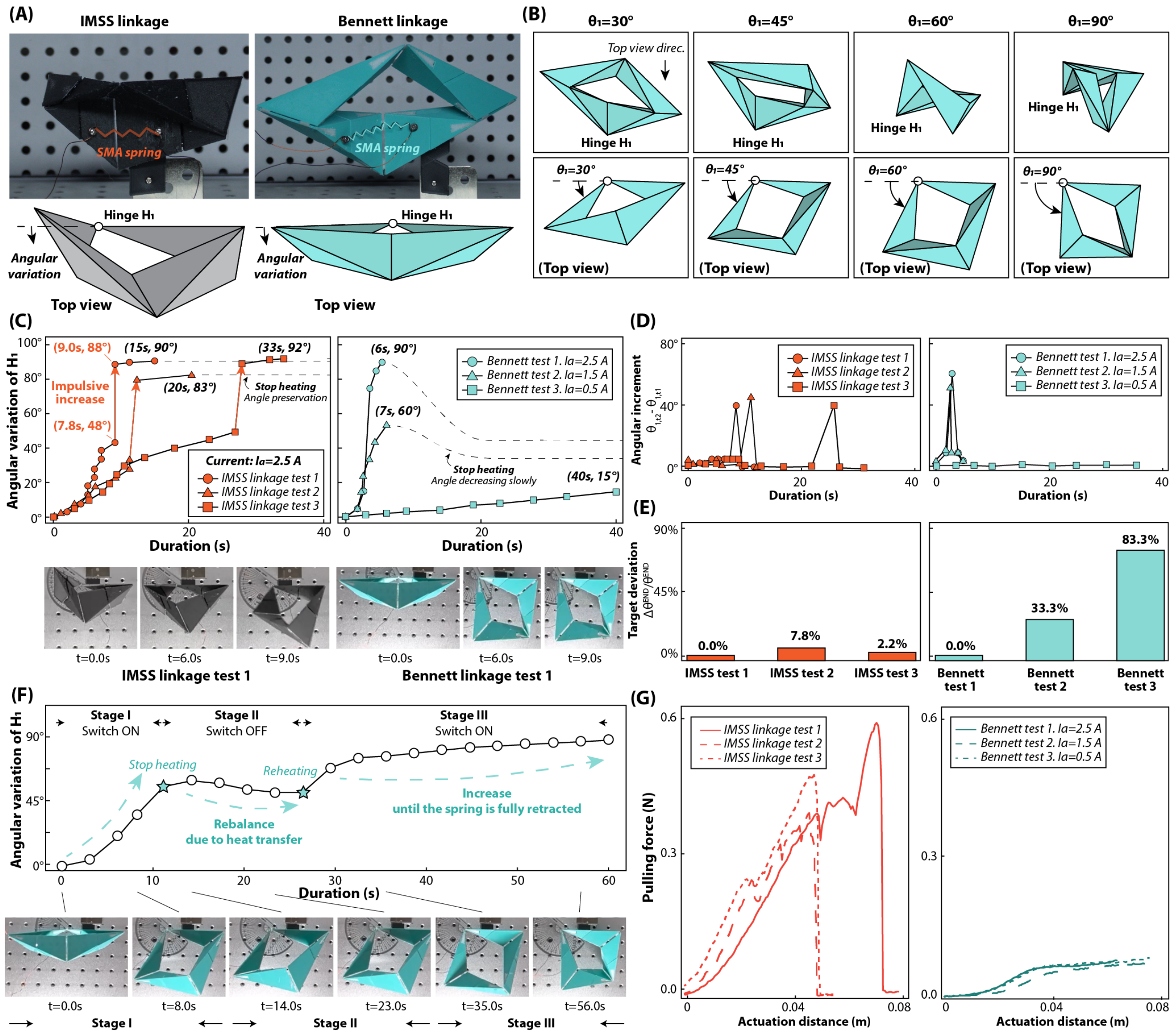


**Fig. S14. Quantitative comparison of an IMSS linkage and a Bennett linkage** (**A**) Physical model of an IMSS linkage and a Bennett linkage. SMA springs are mounted to actuate. The configuration of two linkages is characterized by the angular variation of Hinge $H_1$, $\theta_1$ (**B**) Snapshots of reconfiguration of the Bennett linkage. The angular variation of $H_1$ is indicated in the figure. (**C**) Angular variation of acutating tests. Different amounts of current, $I_a = 1.0\text{A}, 1.5\text{A}, 2.5\text{A}$, are imposed in the SMA spring in the Bennett linkage, where the current in the IMSS linkage stays same, $I_a = 2.5\text{A}$. When heating stops, the angle of the IMSS linkage is unchanged due to stability, while the angle decreases slowly due to the downward temperature. Experimental snapshots are listed below. (**D**) Analysis of shape accuracy of the IMSS linkage and the Bennett linkage. The shape accuracy is characterized by the target deviation of the rotation angle at the end state, $\delta_t = \Delta\theta_1^{\text{END}}/\theta_1^{\text{END}} = \|\theta_1^{\text{END}\prime} - \theta_1^{\text{END}}\|/\theta_1^{\text{END}}$, where the target $\theta_1^{\text{END}} = 90°$ in this case, and $\theta_1^{\text{END}\prime}$ is the real value of the angle. The result suggests a higher shape error in the Bennett linkage after actuation. (**E**) Analysis of impulsivity of the IMSS linkage and the Bennett linkage. The angular increment at each time step indicates that the impulse motion in the Bennett linkage would be more obvious if the current is increased. However, the impulsivity would be unchanged in the IMSS linkage, because it comes from structural instability, instead of actuators. (**F**) Shape accuracy enhancement at the cost of the complexity of controlling strategy. At Stage I, the circuit is connected and the SMA spring is heating. Heating stops when the Bennett linkage arrives at the desired state, $\theta_1 = 55°$. However, a slight decrease is observed after the heating is off, because the heat transfer between SMA and air would lower its temperature, and make the spring recover slightly and slowly. Reheating process would increase the rotation angle at Stage III, and there would be no close-loop controlling strategies in this stage. Therefore, the reconfiguration would continue until the spring is fully retracted. (**G**) Analysis of stiffness of the IMSS linkage and the Bennett linkage. The pulling tests are conducted, where a higher force is observed in the IMSS linkage for its reconfiguration.

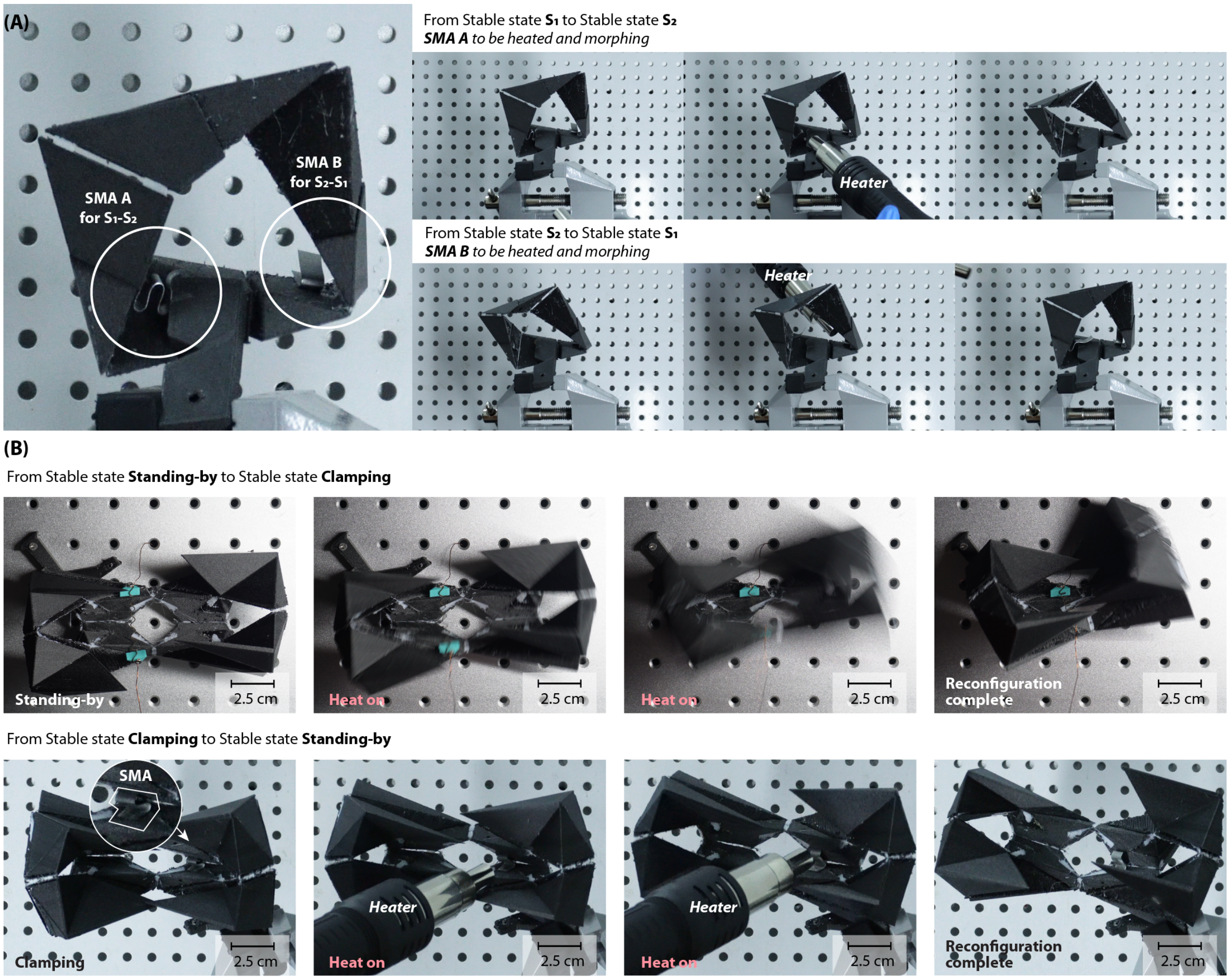


**Fig. S15. Demonstrations of the reversible reconfiguration of bi-stable linkages.** (**A**) Reconfiguration of the IMSS-R4S2 linkage with 2 SMA actuators. Heating SMA A actuator and keeping SMA B inactive could make the reconfiguration from Stable state $S_1$ to Stable state $S_2$, while SMA B for the reverse reconfiguration. The complete process could be found in Supplementary Movie S2. (**B**) Reconfiguration of the gripper with two SMA actuators. The complete process could be found in Supplementary Movie S5.

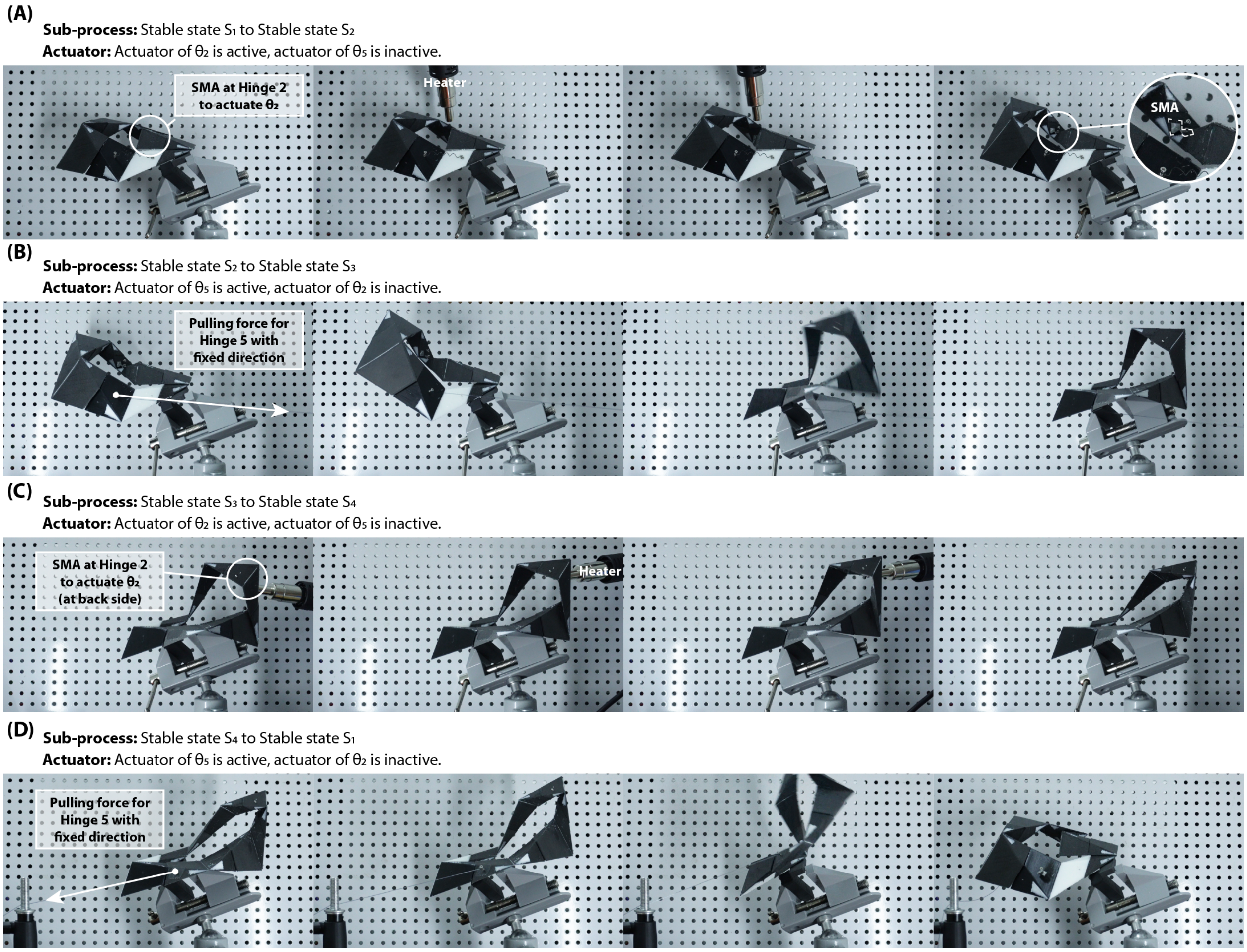


**Fig. S16. Demonstrations of the reversible reconfiguration of the IMSS-R6S4 linkage.** The actuating strategy follows the order described in "2.2 Quadra-stable single-loop spatial 6R linkages" and Fig. 3 of the manuscript. (**A**) Sub-process: Stable state $S_1$ to Stable state $S_2$. One SMA actuator is implemented at Hinge 2 and heated to actuate $\theta_2$, while keeping $\theta_5$ unchanged by inactivating the actuator on Hinge 5. (**B**) Sub-process: Stable state $S_2$ to Stable state $S_3$. Actuating $\theta_5$ by connecting a cable on Hinge 5 and engaging a force with a fixed direction. (**C**) Sub-process: Stable state $S_3$ to Stable state $S_4$. Another SMA actuator is implemented at Hinge 2 and heated to actuate $\theta_2$. (**D**) Sub-process: Stable state $S_4$ to Stable state $S_1$. Actuating $\theta_5$ by connecting a cable on Hinge 5 as well, yet engaging a force with an another fixed direction. In total, the quantities of actuators are four. The complete process could be found in Supplementary Movie S3.

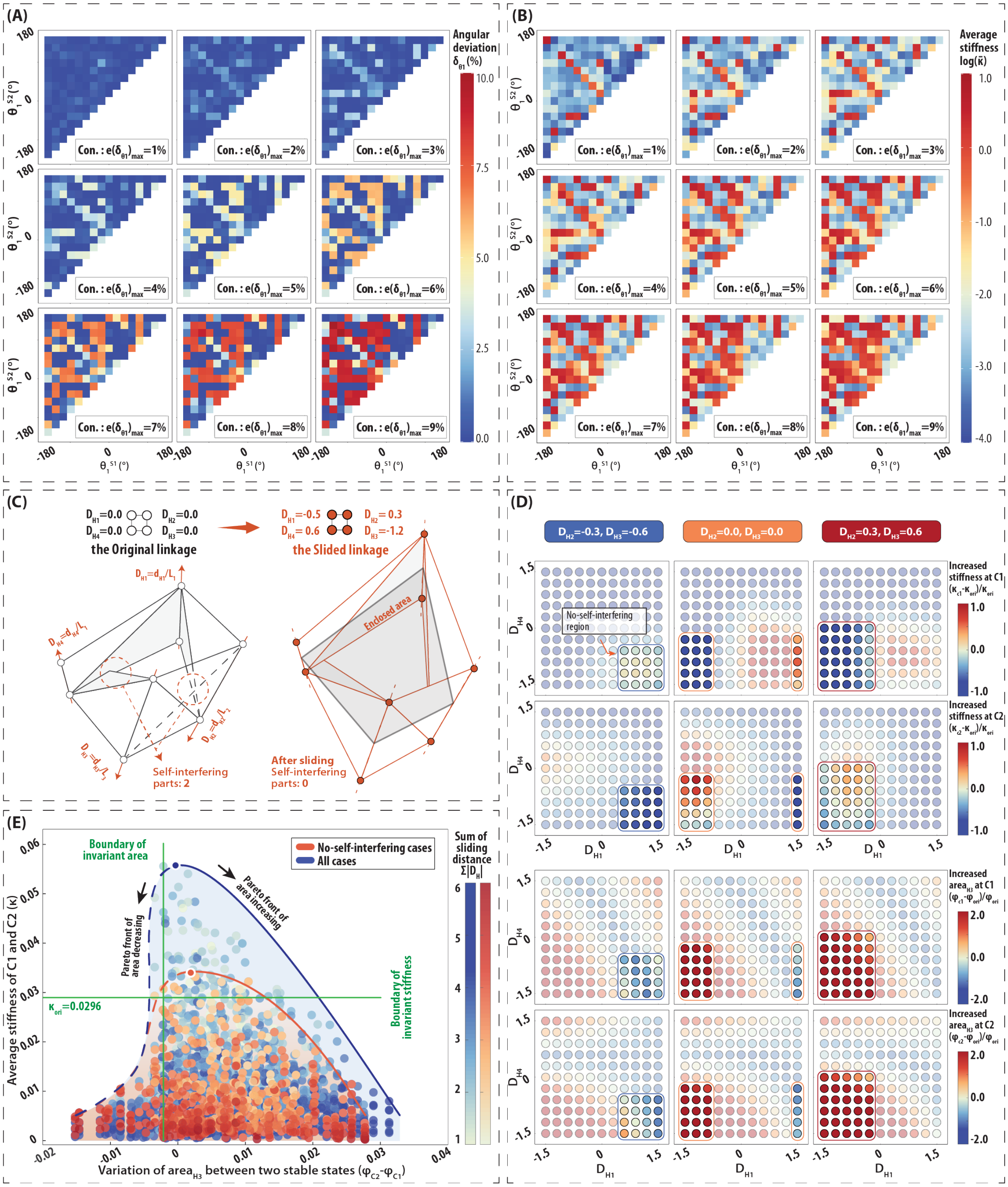


**Fig. S17. Parametric analysis for IMSS-R4S2 linkages and their alternative designs.** (**A**∼**B**) The analysis of the deviation from target stable geometries and stiffness evaluation of bi-stable 4R linkages. Deviation distribution across the combination of different target stable geometries with different deviation upper bound is given in (A), the corresponding structural stiffness is given in (B), and a positive correlation of the two can be observed. (**C**) Alternative designs of single-loop IMSS linkages by introducing hinge sliding. (**D**) Analysis of the variation of the stiffness and the hinge $H_h$-projected area $\phi_h$ when the hinge sliding is applied. As there are four hinges, the sliding distances of hinges $H_2$ and $H_3$ are given first (as shown above the plots), and the influence of the stiffness and the projected area by the sliding distances of hinges $H_1$ and $H_4$ are shown in the plots. (**E**) Pareto analysis of hinges sliding. The range of the cases with no self-interference (orange) according to its Pareto fronts is narrower, which suggests that the hinge sliding for self-interference avoidance contributes to the less projected area and the lower average stiffness.

**Table. S1.** Essential properties of the materials using in the 3D printing

| No. | Property | PolyMide PA12-CF | PolyFlex TPU95 | Testing method |
|---|---|---|---|---|
| 1 | Density | 1.06 $g/cm^3$ | 1.20 1.24 $g/cm^3$ | ISO 1183 |
| 2 | Young's modules (X-Y) | 3304.39 ± 145 MPa (Dry status) | 9.4 ± 0.3 MPa | ISO 527 |
| 3 | Tensile strength (X-Y) | 71.6 ± 1.7 MPa (Dry status) | 29.0 ± 2.8 MPa | ISO 527 |
| 4 | Elongation at break (X-Y) | 3.57 ± 0.3 (Dry status) | 330.1 ± 14.9 % | ISO 527 |

**Table. S2.** Stiffness of the equivalent spring of each sample of IMSS-R4S2 linkage

| Sample | $t_h$ (mm) | $b_h$ (mm) | $\kappa_{to}$ | $\kappa_{tr}$ |
|---|---|---|---|---|
| 1 | 0.5 | 2.0 | 0.000245 | 11750 |
| 2 | 0.5 | 1.0 | 0.000490 | 23500 |
| 3 | 0.5 | 0.5 | 0.000979 | 47000 |
| 4 | 0.3 | 1.0 | 0.000106 | 14100 |
| 5 | 2.0 | 2.0 | 0.015700 | 47000 |

**Description of Additional Supplementary Files**

1. Movies S1 to S7

   **S1** : Introductory video for the basic idea of this work.

   **S2** : Video for the experiments of the intrinsically bi-stable 4R spatial (IMSS-R4S2) linkages.

   **S3** : Video for the experiments of the intrinsically quadra-stable 6R spatial (IMSS-R6S4) linkages.

   **S4** : Video for the reconfiguration of the multi-loop assembly: the deployable tube.

   **S5** : Video for the reconfiguration of the multi-loop assembly: the impulsive gripper.

   **S6** : Video for the experiments of the intrinsically tri-stable 6R spatial (IMSS-R6S3) linkages.

   **S7** : Video for the reconfiguration of the multi-loop assembly: the swimming robot.

2. Data S1 to S9

   **S1** : Codes for designing IMSS-R4S2, IMSS-R6S4, IMSS-R6S3 linkages.

   **S2** : Codes for analyzing IMSS-R4S2, IMSS-R6S4, IMSS-R6S3 linkages, and the reconfigurable tube, the impulsive gripper.

   **S3** : Codes for local stiffness calculation.

   **S4** : Codes for derivation of the closure condition.

   **S5** : Codes for self-interfering detection based on G-J-K algorithm.

   **S6** : Codes for for unwrapping IMSS linkages.

   **S7** : Pattern files for unwrapped IMSS linkages.

   **S8** : Technical sheet for materials used in 3D printing.

   **S9** : Computation results needed for the paper.
