# Supplementary material for: Intrinsically Multi‐Stable Spatial Linkages
Source: Adv Sci (Weinh). 2024 Sep 16;11(41):2402127. doi: 10.1002/advs.202402127 (PMC11538711; doi:10.1002/advs.202402127)
Supplement: Supplementary file 9 — Supporting Information [file ADVS-11-2402127-s002.zip › SM_Data/Data_S8_TechnicalDataSheet/Data_S8_TechnicalDataSheet_Carbonfibre.pdf]

polymaker

Innovators in 3D printing

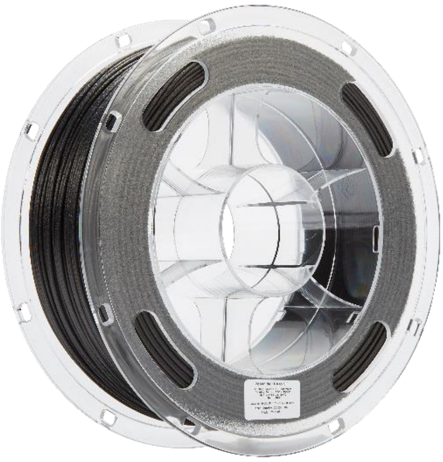

Technical Data Sheet

PolyMide™ PA12-CF

www.polymaker.com

V5.1

PolyMide™ PA12-CF

PolyMide™ PA12-CF is a carbon fiber reinforced PA12 (Nylon 12) filament. Thanks to the low moisture sensitivity of PA12, this product features outstanding mechanical and thermal properties even after the moisture conditioning process. Combined with its ease of print with Warp-Free™ technology, this product is ideal to create manufacturing tools.

PHYSICAL PROPERTIES

Property	Testing Method	Typical Value
Density	ISO1183, GB/T1033	1.06 g/cm^3 at 23°C
Melt index	280°C, 2.16 kg	25 g/10min

CHEMICAL RESISTANCE DATA

Property	Testing Method
Effect of weak acids	Not resistant
Effect of strong acids	Not resistant
Effect of weak alkalis	Slight resistant
Effect of strong alkalis	Not resistant
Effect of organic solvent	Not resistant
Effect of oils and grease	Resistant

MOISTURE ABSORPTION CURVE

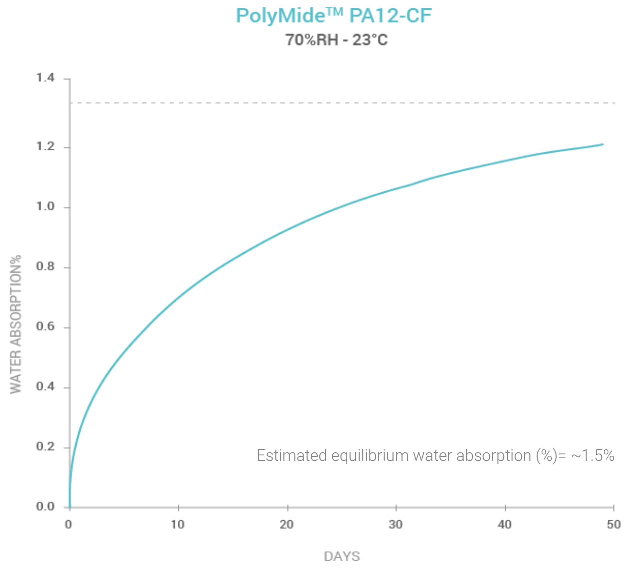

Note:

PolyMide™ PA12-CF absorbs moisture slowly over the time. The estimated equilibrium water absorption is around 1.5%.

THERMAL PROPERTIES

Property	Testing Method	Typical Value
Glass transition temperature	DSC, 10°C/min	54°C
Melting temperature	DSC, 10°C/min	165°C
Crystallization temperature	DSC, 10°C/min	130°C
Decomposition temperature	TGA, 20°C/min	N/A
Vicat softening temperature	ISO 306, GB/T 1633	N/A
Heat deflection temperature	ISO 75 1.8MPa	105°C
Heat deflection temperature	ISO 75 0.45MPa	131°C
Thermal conductivity	N/A	N/A
Heat shrinkage rate	N/A	N/A

HDT CURVE

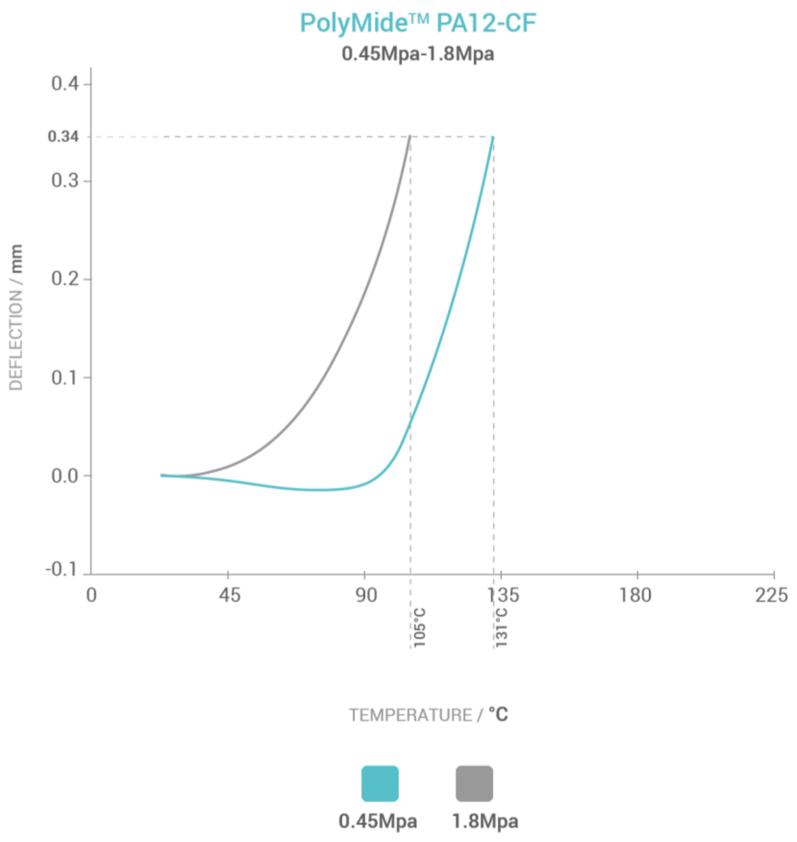

MECHANICAL PROPERTIES (Dry status)

Property	Testing Method	Typical Value
Young’s modulus (X-Y)	ISO 527, GB/T 1040	3304.39 ± 145 MPa
Young’s modulus (Z)		1801.09 ± 80 MPa
Tensile strength (X-Y)	ISO 527, GB/T 1040	71.6 ± 1.7 MPa
Tensile strength (Z)		43.3 ± 3.3 MPa
Elongation at break (X-Y)	ISO 527, GB/T 1040	3.57 ± 0.3 %
Elongation at break (Z)		3.29 ± 0.5%
Bending modulus (X-Y)	ISO 178, GB/T 9341	3535 ± 239 MPa
Bending modulus (Z)		N/A
Bending strength (X-Y)	ISO 178, GB/T 9341	109.9 ± 1.4 MPa
Bending strength (Z)		N/A
Charpy impact strength (X-Y)	ISO 179, GB/T 1043	12.5 ± 0.7 kJ/m^2
Charpy impact strength (Z)		N/A

Note:
All specimens were annealed at 80˚C for 24h and dried for 48h prior to testing

MECHANICAL PROPERTIES (Wet Status)

Property	Testing Method	Typical Value
Young’s modulus (X-Y)	ISO 527, GB/T 1040	3054 ± 149 MPa
Young’s modulus (Z)		1520 ± 84 MPa
Tensile strength (X-Y)	ISO 527, GB/T 1040	73.4 ± 0.6 MPa
Tensile strength (Z)		42.0 ± 1.5 MPa
Elongation at break (X-Y)	ISO 527, GB/T 1040	6.06 ± 0.6%
Elongation at break (Z)		3.51 ± 0.3%
Bending modulus (X-Y)	ISO 178, GB/T 9341	3336 ± 292 MPa
Bending modulus (Z)		N/A
Bending strength (X-Y)	ISO 178, GB/T 9341	100.9 ± 3.5 MPa
Bending strength (Z)		N/A
Charpy impact strength (X-Y)	ISO 179, GB/T 1043	9.2 ± 0.6 kJ/m^2
Charpy impact strength (Z)		N/A

Note:
All specimens were annealed at 80 °C for 24h, and immerged in ambient temperature water for 3 days prior to testing

RECOMMENDED PRINTING CONDITIONS

* Based on 0.4 mm nozzle and Simplify 3D v.4.0. Printing conditions may vary with different nozzle diameters

Parameter	
Nozzle temperature	260 – 300 (℃)
Build surface material	BuildTak®, Glass, Blue Tape
Build surface treatment	PVP Glue
Build plate temperature	25 - 50 (˚C)
Cooling fan	OFF
Printing speed	30-60 (mm/s)
Raft separation distance	0.2 (mm)
Retraction distance	3 (mm)
Retraction speed	40 (mm/s)
Environmental temperature	Room temperature
Threshold overhang angle	60 (˚)
Recommended support material	N/A

Note:

- Abrasion of the brass nozzle happens frequently when printing PolyMide™ PA12-CF. Normally, the life of a brass nozzle would be approximately 9h. A wear-resistance nozzle, such as hardened steel and ruby nozzle, is highly recommended to be used with PolyMide™ PA12-CF.
- If PolyMide™ PA12-CF is used as the support material for itself, please remove the support structure before excessive moisture absorption. Otherwise the support structure can be permanently bonded to the model.
- After the printing process, it is recommended to anneal the model in the oven at 80°C for 6 hours.

TENSILE TESTING SPECIMEN

ISO 527, GB/T 1040

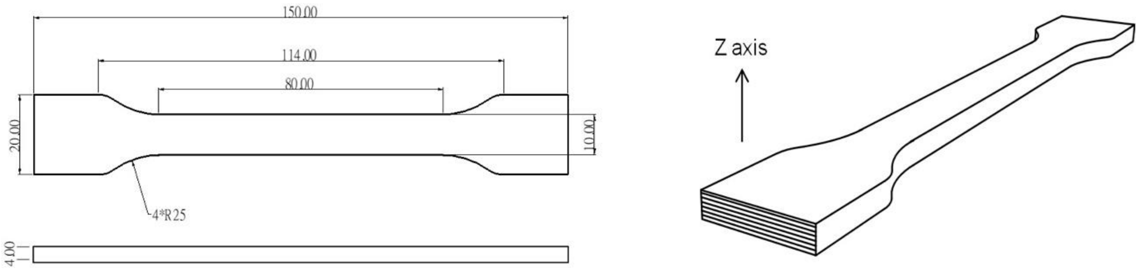

FLEXURAL TESTING SPECIMEN

ISO 178, GB/T 9341

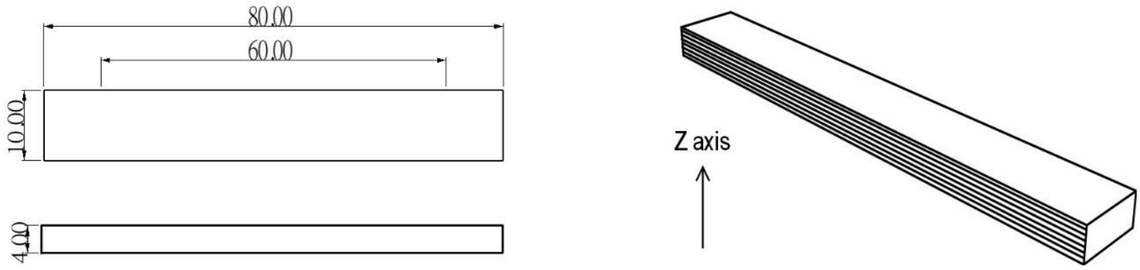

IMPACT TESTING SPECIMEN

ISO 179, GB/T 1043

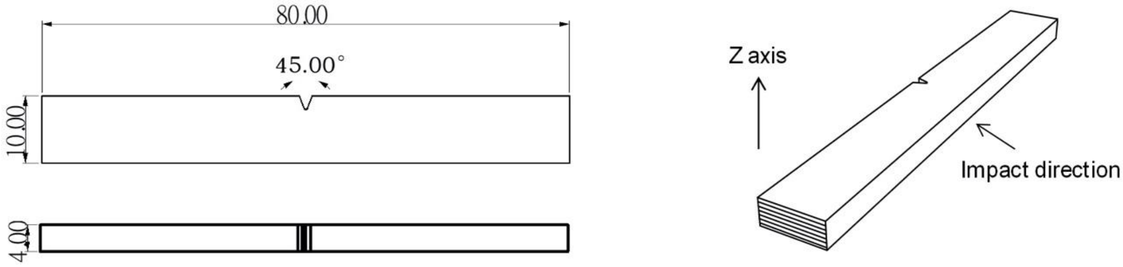

HOW TO MAKE SPECIMENS

Printing temperature	300 °C
Bed temperature	50 °C
Shell	2
Top & bottom layer	4
Infill	100%
Environmental temperature	50 ˚C
Cooling fan	OFF

DISCLAIMER:

The typical values presented in this data sheet are intended for reference and comparison purposes only. They should not be used for design specifications or quality control purposes. Actual values may vary significantly with printing conditions. End- use performance of printed parts depends not only on materials, but also on part design, environmental conditions, printing conditions, etc. Product specifications are subject to change without notice.

Each user is responsible for determining the safety, lawfulness, technical suitability, and disposal/ recycling practices of Polymaker materials for the intended application. Polymaker makes no warranty of any kind, unless announced separately, to the fitness for any use or application. Polymaker shall not be made liable for any damage, injury or loss induced from the use of Polymaker materials in any application.
