# Supplementary material for: Intrinsically Multi‐Stable Spatial Linkages
Source: Adv Sci (Weinh). 2024 Sep 16;11(41):2402127. doi: 10.1002/advs.202402127 (PMC11538711; doi:10.1002/advs.202402127)
Supplement: Supplementary file 9 — Supporting Information [file ADVS-11-2402127-s002.zip › SM_Data/Data_S8_TechnicalDataSheet/Data_S8_TechnicalDataSheet_TPU.pdf]

PolyFlex™ TPU95

PolyFlex™ TPU95 is a thermoplastic polyurethane (TPU) based filament specifically engineered to work on most desktop 3D printers. It has a shore hardness of 95A and can stretch more than 3 times its original length.

Physical Properties

Property	Testing method	Typical value
Density	ASTM D792 (ISO 1183, GB/T 1033)	1.20 – 1.24 (g/cm3 at 21.5˚C)
Melt index	210 °C, 1.2 kg	3-6 (g/10 min)

Tested with 3D printed specimen of 100% infill

Mechanical Properties

Property	Testing method	Typical value
100% modulus (X-Y)	ASTM D638 (ISO 527, GB/T 1040)	9.4 ± 0.3 (MPa)
Tensile strength (X-Y)	ASTM D638 (ISO 527, GB/T 1040)	29.0 ± 2.8 (MPa)
Elongation at break (X-Y)	ASTM D638 (ISO 527, GB/T 1040)	330.1 ± 14.9 (%)
Shore hardness	ASTM D2240 (ISO 7619, GB/T 31)	~95A

All testing specimens were printed under the following conditions:
nozzle temperature = 225 °C, printing speed = 30 mm/s, build plate temperature = 30 °C, infill = 100%
All specimens were conditioned at room temperature for 24h prior to testing

Recommended printing conditions

Parameter	
Nozzle temperature	210 - 230 (˚C)
Build Surface material	BuildTak®, Glass, Blue Tape
Build surface treatment	None, Applying PVA glue to the build surface
Build plate temperature	25 - 60 (˚C)
Cooling fan	Turned on
Printing speed	20-40 (mm/s)
Raft separation distance	0.2 (mm)
Retraction distance	1 (mm)
Retraction speed	20 (mm/s)
Recommended environmental temperature	Room temperature - 45 (˚C)
Threshold overhang angle	35 (˚)
Recommended support material	PolySupport™ and PolyDissolve™ S1

Based on 0.4 mm nozzle and Simplify 3D v.3.1. Printing conditions may vary with different nozzle diameters

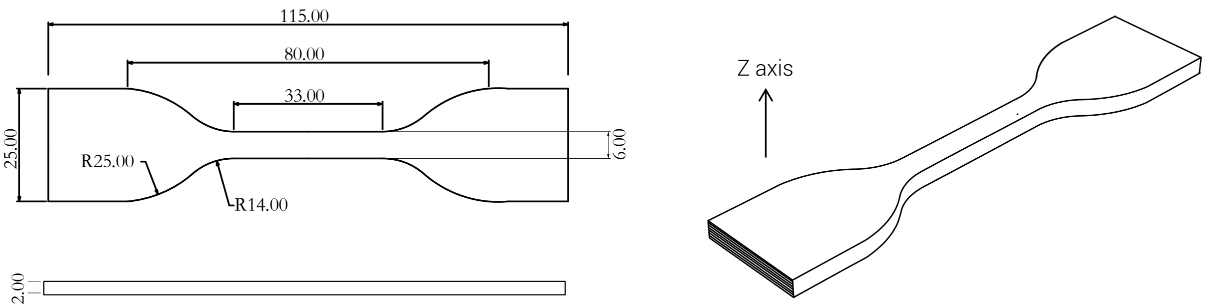

Tensile testing specimen; ASTM D638 (ISO 527, GB/T 1040)

Disclaimer:

The typical values presented in this data sheet are intended for reference and comparison purposes only. They should not be used for design specifications or quality control purposes. Actual values may vary significantly with printing conditions. End- use performance of printed parts depends not only on materials, but also on part design, environmental conditions, printing conditions, etc. Product specifications are subject to change without notice.

Each user is responsible for determining the safety, lawfulness, technical suitability, and disposal/recycling practices of Polymaker materials for the intended application. Polymaker makes no warranty of any kind, unless announced separately, to the fitness for any use or application. Polymaker shall not be made liable for any damage, injury or loss induced from the use of Polymaker materials in any application.
